# Supplementary material for: Nonporous amorphous superadsorbents for highly effective and selective adsorption of iodine in water
Source: Nat Commun. 2023 Sep 4;14:5388. doi: 10.1038/s41467-023-41056-5 (PMC10477329; doi:10.1038/s41467-023-41056-5)
Supplement: Supplementary file 1 — Supplementary Information [file 41467_2023_41056_MOESM1_ESM.pdf]

Supplementary Information

Nonporous Amorphous Superadsorbents for Highly Effective and Selective Adsorption of Iodine in Water

Wei Zhou,[1] Aimin Li,[1] Min Zhou,[1,2] Yiyao Xu,[1] Yi Zhang,[1] and Qing He*,[1]

[1]State Key Laboratory of Chemo/Biosensing and Chemometrics, College of Chemistry and Chemical Engineering, Hunan University, Changsha 410082, P.R. China

[2]College of Chemistry and Chemical Engineering, Hunan Normal University, Changsha, P. R. China.

*Corresponding Author: Qing He: heqing85@hnu.edu.cn

Contents

Supplementary Note 1. Synthesis

Supplementary Note 2. Iodine vapor adsorption

Supplementary Note 3. Static iodine adsorption from contaminated water

Supplementary Note 4. Dynamic flow–through adsorption from aqueous iodine media

Supplementary Note 5. Recyclability and reusability

Supplementary Note 6. Mechanistic Study

Supplementary Note 7. X–ray experimental details

Supplementary Note 8. HRMS spectra and NMR spectra

Supplementary Note 9. Energies and geometrical coordinates of the optimized models in the gas phase

Supplementary References

Supplementary Note 1. Synthesis

Synthesis of S1

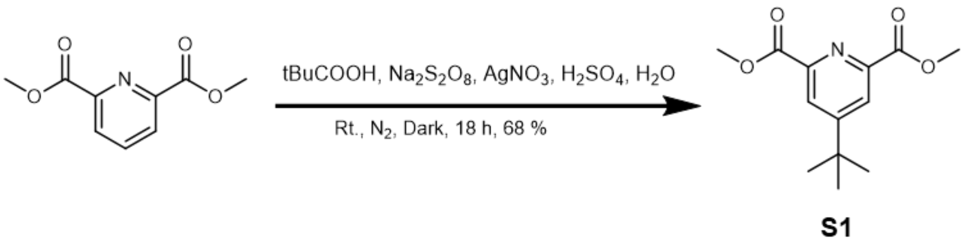

Supplementary Fig. 1 | Synthesis of **S1**.

S1 was synthesized according to the literature.[1] Dimethylpyridine-2,6-dicarboxylate (100 g, 0.52 mol) and 1500 mL of deionized water were charged to a 2000 mL round bottom flask under N_2 atmosphere. Concentrated sulfuric acid (46 mL) was slowly added, and stirred at room temperature for 12 h. Then the trimethylacetic acid (157.65 g, 1.53 mol), silver nitrate (8.80 g, 0.052 mol) and sodium peroxydisulfate (367.75 g, 1.53 mol) were sequentially added to the flask. The mixture was stirred at room temperature for 18 h in the dark under the N_2 atmosphere. The pH value of the aqueous phase was alkalinized with powdered NaOH. The reaction miture was extracted with 3000 mL ethyl acetate, then was filtered through a pad of Celite bed, and all organic layers were combined, washed with water (3 × 500 mL) and brine (3× 500 mL), respectively. and dried over Na_2SO_4. After removing the solvent by reduced pressure, the mixture was recrystallizated from approximately 500 mL EA to afforde **S1** as a white crystalline solid. (87.0 g, 68 %). ^{1}H NMR (400 MHz, $CDCl_3$) δ 8.29 (s, 2H), 4.00 (s, 6H), 1.37 (s, 9H). ^{1}H NMR (400 MHz, $CDCl_3$) δ 8.29 (s, 2H), 4.00 (s, 6H), 1.37 (s, 9H), which is in accord with the reported data.[1]

Synthesis of S2

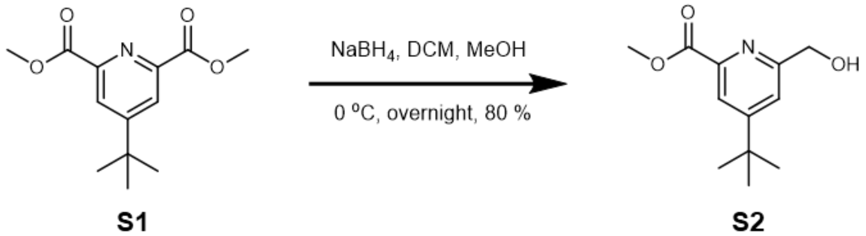

Supplementary Fig. 2 | Synthesis of **S2**.

S2 was prepared according to the reported method.[2] Into a 1000 mL round-bottom flask was placed a solution of **S1** (50 g, 0.20 mol) in a solvent mixture of methanol (350 mL) and dichloromethane (350 mL). $NaBH_4$ (19 g, 0.50 mol) was added to the reaction mixture in batches at 0 °C, then the resulting solution was stirred overnight. After removing the solvent by reduced pressure, to the resulting residue was added 1000 mL of water, followed by extraction with EA (2 × 300 mL). The organic phases were combined and dried over anhydrous Na_2SO_4. After concentration under vacuum, the residue was purified by flash chromatography on silica gel using a mixture of ethyl acetate/petroleum ether (1:1→2:1, v/v) as eluent to yield of **S2** as a white solid (35.7 g, 80 %). ^{1}H NMR (400 MHz, $CDCl_3$) δ 8.04 (s, 1H), 7.48 (s, 1H), 4.83

(s, 2H), 3.99 (s, 3H), 1.35 (s, 9H), which is consistent with the reported data.[2]

Synthesis of 1

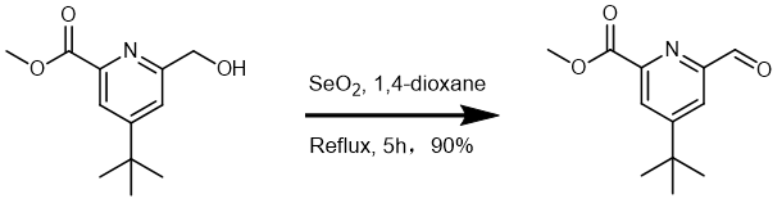

S2 **1**

Supplementary Fig. 3 | Synthesis of **1**.

Compound **1** was obtained according to the reported synthetic approach.[2] To a round-bottom flask with a stirred solution of compound **S2** (35 4.50 g, 0.209 mol) in 1,4-dioxane (50 ml.) was added SeO_2 (11.67 g, 0.105 mmol). The mixture was then refluxed for 3 h. After the reaction completed, the hot mixture was clarified by filtering through a Celite bed and the filtrate was concentrated under vacuo. The crude mixture was purified by chromatography to yield of **1** as a yellow oil (41.6 g, 90 %). ^{1}H NMR (400 MHz, $CDCl_3$) δ 10.18 (s, 1H), 8.35 (s, 1H), 8.14 (s, 1H), 4.06 (s, 3H), 1.39 (s, 9H).[2]

Synthesis of 2

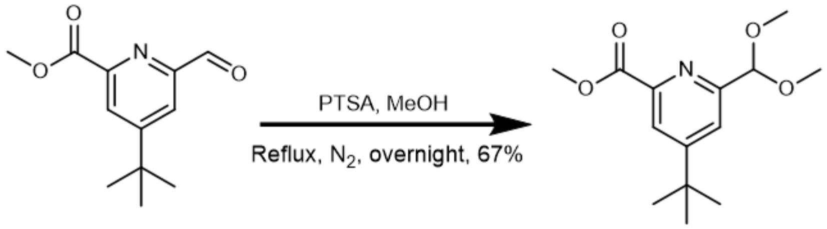

1 **2**

Supplementary Fig. 4 | Synthesis of **2**.

To a solution of **1** (20 g, 90.45 mmol) in methanol (400 mL), *p*–toluenesulfonic acid monohydrate (30 mol %, 5.17 g) was added. The reaction mixture was then refluxed overnight under N_2 atmosphere. After cooling to room temperature, the resulting solution was concentrated and the residues were dissolved in ethyl acetate (500 mL). Subsequently, the organic phase was consecutively washed with brine (2 × 200 mL) and water (2 × 200 mL). The organic phases were separated and combined, then dried over with anhydrous Na_2SO_4. After removing the solvent by reduced pressure, the residue was subjected to silica–gel column chromatography using a mixture of petroleum ether / ethyl acetate (7:1→1:1, v/v) as eluent to obtain compound **2** as yellow oil (16.2 g, 67%). ^{1}H NMR (400 MHz, $CDCl_3$) δ 8.10 (s, 1H), 7.71 (s, 1H), 5.41 (s, 1H), 3.98 (s, 3H), 3.42 (s, 6H), 1.34 (s, 9H). ^{13}C NMR (101 MHz, $CDCl_3$) δ 166.3, 162.5, 158.0, 147.4, 122.6, 121.2, 105.0, 54.5, 53.0, 35.3, 30.6. HRMS (ESI) *m/z*: $[M+H]^+$ calcd for $C_{14}H_{22}NO_4^+$ 268.1543, found 268.1537.

Synthesis of 3

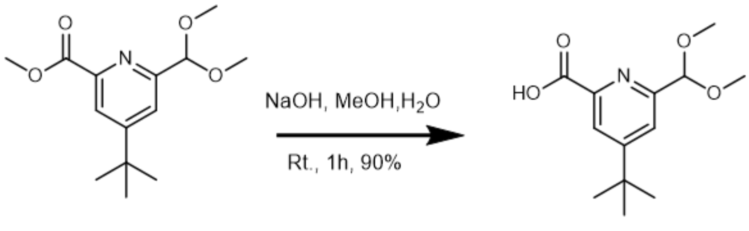

2 3

Supplementary Fig. 5 | Synthesis of **3**.

To a solution of compound **2** (10 g, 37.43 mmol) in MeOH (100 mL) at 0 °C, a solution of NaOH (4.5 g, 112.29 mmol) in water (100 mL) was added dropwise via an addition funnel. After completion of the addition, the reaction mixture was warmed to room temperature and stirred for 12 h. MeOH was then evaporated under reduced pressure and the remaining solution was acidified with diluted HCl (1 M) to ~ pH 3. Next, the mixture solution was extracted with ethyl acetate (2 × 200 mL). The organic phases were combined and extensively washed with brine (2 × 100 mL). Finally, the organic layer was separated and dried by anhydrous Na_2SO_4. Solvent was totally removed to give compound **3** as yellow oil (8.5 g, 90 %). ^{1}H NMR (400 MHz, $CDCl_3$) δ 10.11 (s, 1H), 8.18 (s, 1H), 7.78 (s, 1H), 5.37 (s, 1H), 3.40 (s, 6H), 1.34 (s, 9H). ^{13}C NMR (101 MHz, $CDCl_3$) δ164.8, 164.3, 156.4, 145.6, 122.5, 121.0, 103.5, 54.0, 35.5, 30.5. HRMS (ESI) *m/z*: [M+H] $^+$ calcd for $C_{13}H_{20}NO_4^+$ 254.1387, found 254.1382.

Synthesis of 5

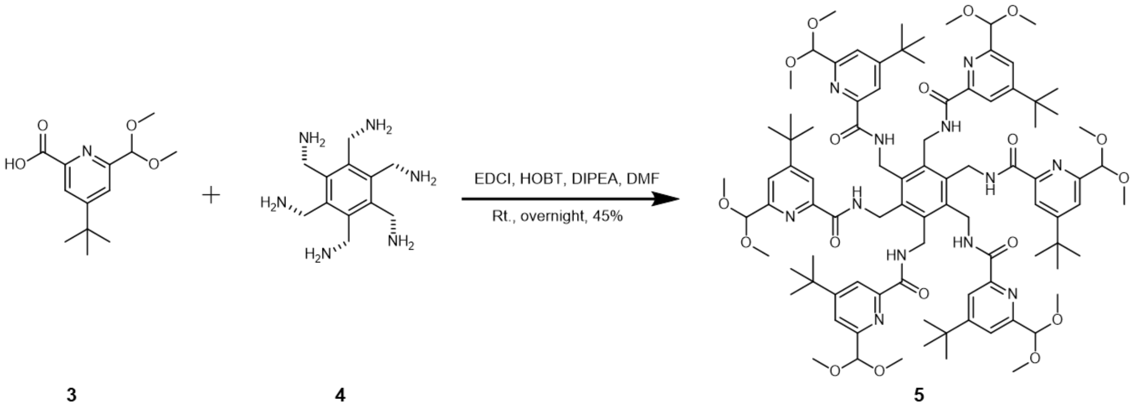

3 4 5

Supplementary Fig. 6 | Synthesis of **5**.

To a mixture of hexakis–amine **4** (1.38 g, 5.5 mmol), **3** (10 g, 39.6 mmol), EDCI (1–ethyl–3–(3–dimethylaminopropyl)–carbodiimide) (7.6 g, 39.6 mmol) and HOBT (hydroxybenzotriazole) (5.35 g, 39.6 mmol) in dry DMF (200 mL) was added DIPEA (N, N–diisopropyethylamine) (7.5 mL, 39.6mmol) via syringe. The reaction mixture was stirred at room temperature under an atmosphere of nitrogen for 24 h. After completion of the reaction, the mixture was diluted with a large amount of H_2O and extracted with EtOAc (3 × 200 mL). The organic layers were combined and washed with brine (2 × 100 mL) and dried

by anhydrous Na_2SO_4. After removal of the solvent, the residue was further purified by silica–gel column chromatography using a mixture of DCM / MeOH (100:1→20:1, v/v) as eluent to give compound **5** as brownish solid (4.1 g, 45%). ^1H NMR (400 MHz, $CDCl_3$) δ 8.71 (s, 6H), 8.15 (s, 6H), 7.58 (s, 6H), 5.22 (s, 6H), 5.02 (s, 12H), 3.30 (d, J = 2.3 Hz, 36H), 1.29 (d, J = 2.6 Hz, 54H). ^{13}C NMR (100 MHz, $CDCl_3$) δ 164.8, 162.4, 156.2, 149.0 138.1, 120.2, 119.7, 104.4, 54.0, 38.3, 35.3, 30.6. HRMS (ESI) *m/z*: $[M+H]^+$ calcd for $C_{90}H_{127}N_{12}O_{18}^+$ 1663.9386, found 1663.9374.

Synthesis of 6

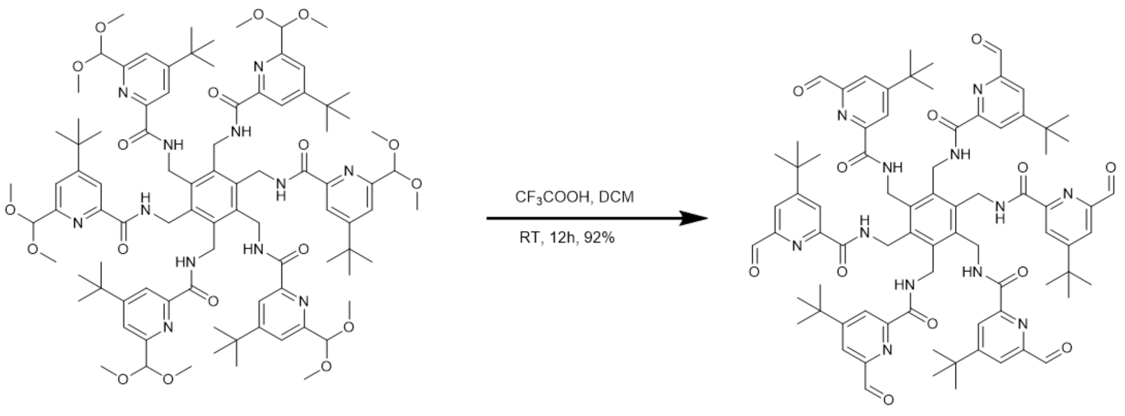

5

6

Supplementary Fig. 7 | Synthesis of **6**.

Compound **5** (4 g, 2.4 mmol) was dissolved in 80 mL of CH_2Cl_2/TFA (4:1, v/v) and stirred for 24 h at room temperature. After completion of the reaction, the solvent was removed under vacuum and the crude product was washed with a large excess of ethyl acetate to yield **6** as white solid (3.1 g, 92%). ^1H NMR (400 MHz, $CDCl_3$) δ 9.68 (s, 6H), 8.98 (s, 6H), 8.33 (d, J = 1.9 Hz, 6H), 7.85 (d, J = 1.9 Hz, 6H), 5.11 (d, J = 5.5 Hz, 12H), 1.31 (s, 54H). ^{13}C NMR (101 MHz, $CDCl_3$) δ 192.3, 164.1, 164.0, 151.2, 150.1, 138.1, 123.5, 120.8, 39.0, 35.6, 30.4. HRMS (ESI) *m/z*: $[M-H]^-$ calcd for $C_{78}H_{90}O_{12}N_{12}^-$ 1385.6728, found 1385.6736.

Synthesis of 7

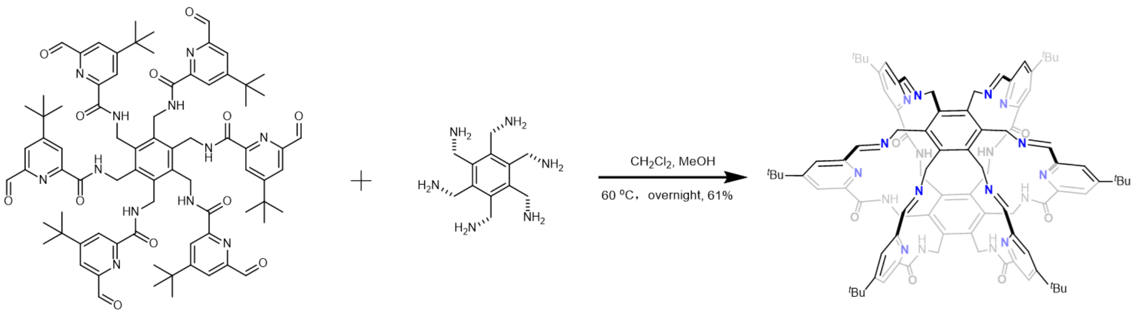

6

4

7

Supplementary Fig. 8 | Synthesis of **7**.

Synthesis of 8

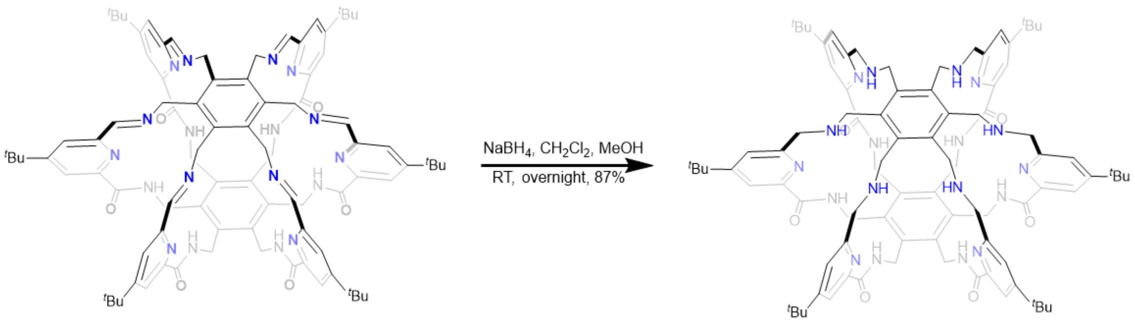

7 8

Supplementary Fig. 9 | Synthesis of **8**.

Supplementary Note 2. Iodine vapor adsorption

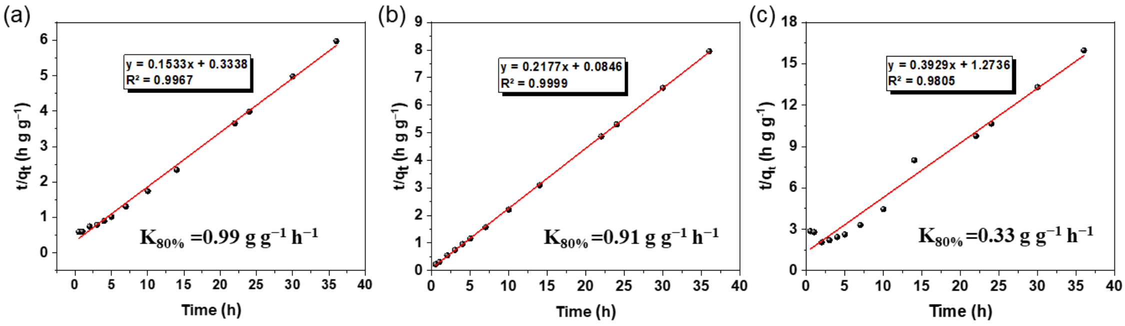

Supplementary Fig. 10 | Pseudo–second order kinetic model of iodine vapor uptake at 348 K and ambient pressure for (a) **SUPE–py–Imine–Cage** , (b) **SUPE–py–Amine–Cage** and (c) Activated carbons (ACs). Note：$K_{80\%}$: In order to quantify the adsorption kinetics of **SUPE–py–Imine–Cage**, **SUPE–py–Amine–Cage** and ACs with higher I_2 uptakes, their average I_2 adsorption rates were calculated through the division of the 80% saturated adsorption capacity by time.[3]

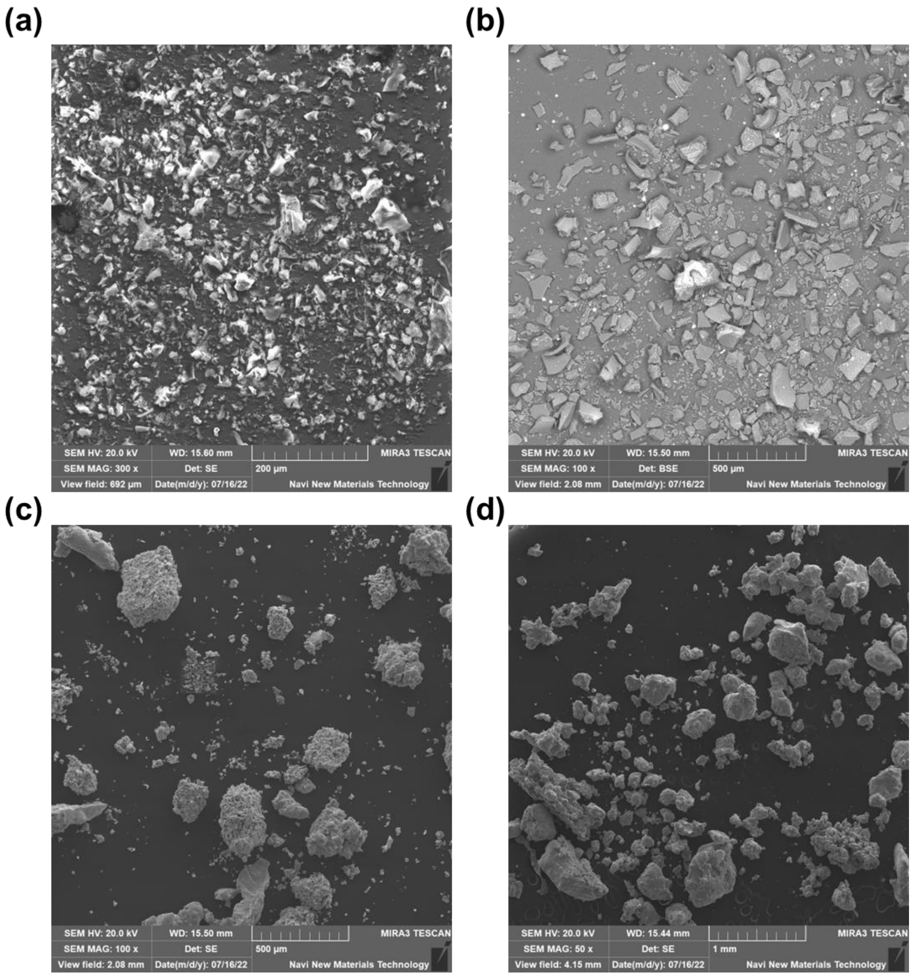

Supplementary Fig. 11 | The SEM images of (a) **SUPE–py–Amine–Cage**, (b) **SUPE–py–Imine–Cage**, (c) I_2@**SUPE–py–Amine–Cage** and (d) I_2@**SUPE–py–Imine–Cage**.

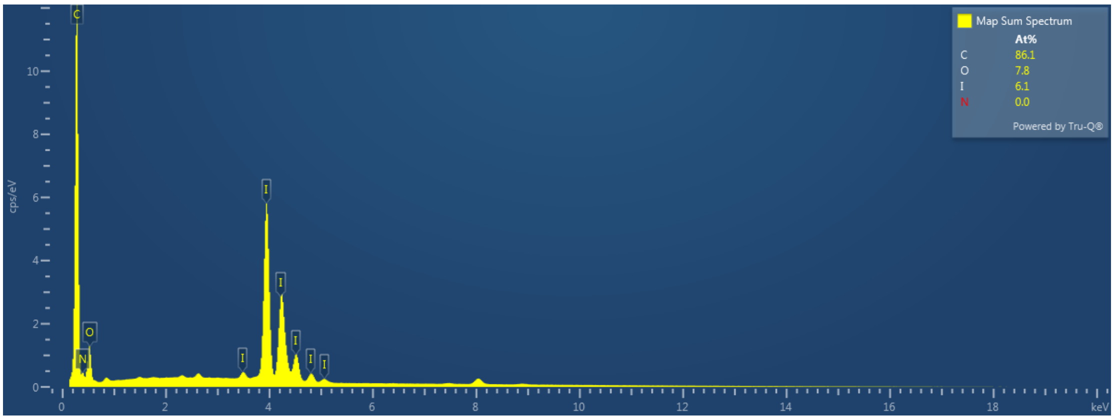

Supplementary Fig. 12 | SEM–EDS results of I_2@**SUPE–py–Amine–Cage**.

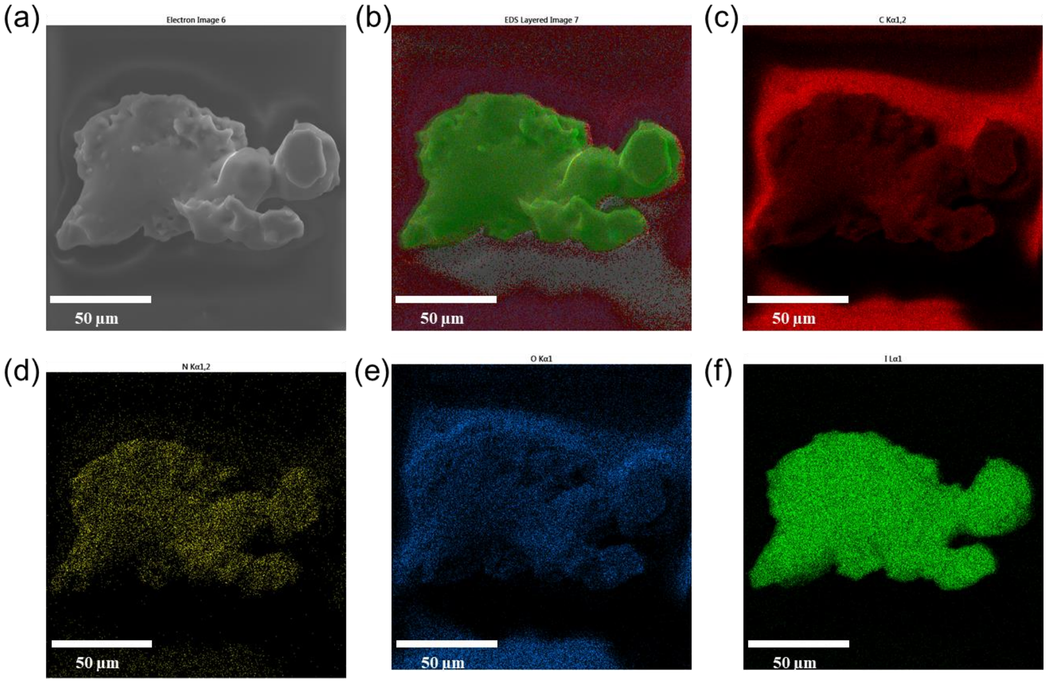

Supplementary Fig. 13 | SEM and EDS mapping results. (a) SEM image of I_2@**SUPE–py–Imine–Cage**, (b) EDS layered image and their SEM/EDS mapping for (c) C, (d) N, (e) O and (f) I.

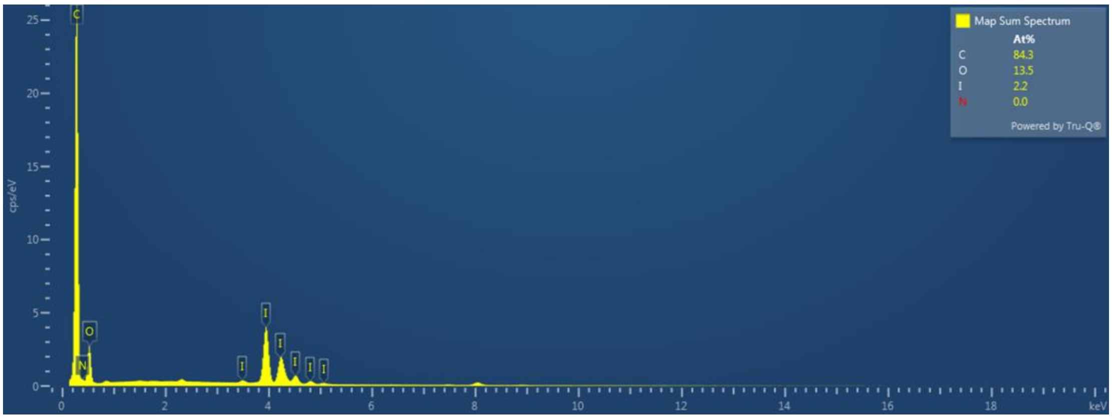

Supplementary Fig. 14 | SEM–EDS results of I_2@**SUPE–py–Imine–Cage**.

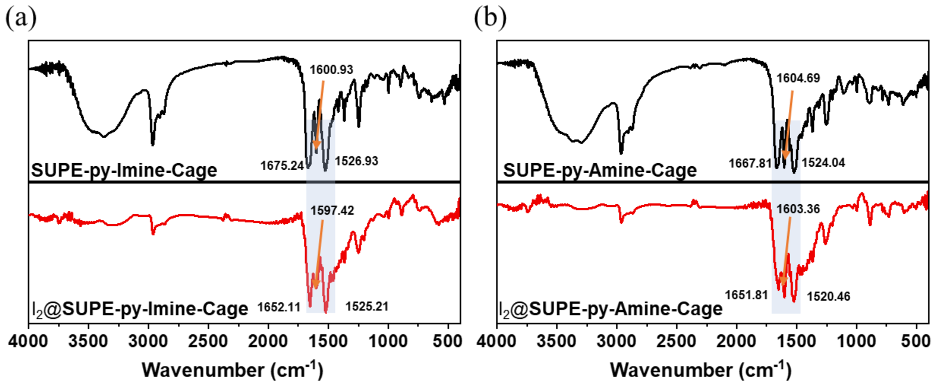

Supplementary Fig. 15 | FTIR spectra of (a) **SUPE–py–Imine–Cage** and (b) **SUPE–py–Amine–Cage** before and after exposure to iodine vapor for 36 h.

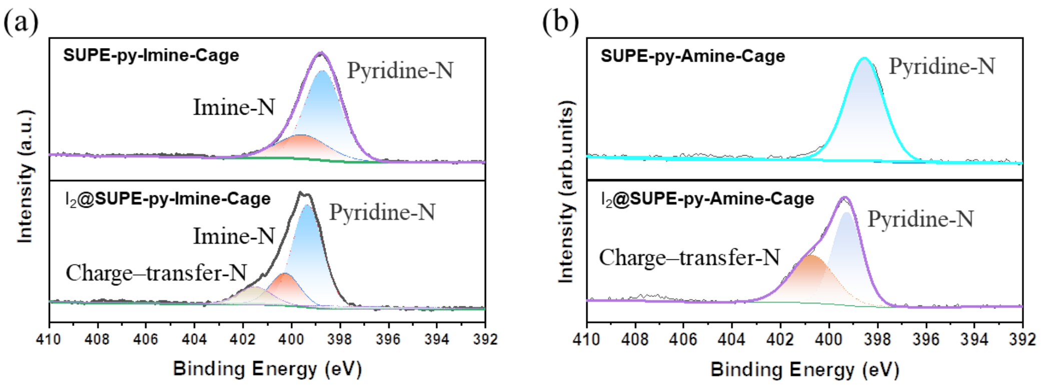

Supplementary Fig. 16 | XPS spectra of *N 1s* for (a) **SUPE–py–Imine–Cage** and (b) **SUPE–py–Amine–Cage** before and after exposure to iodine vapor for 36 h.

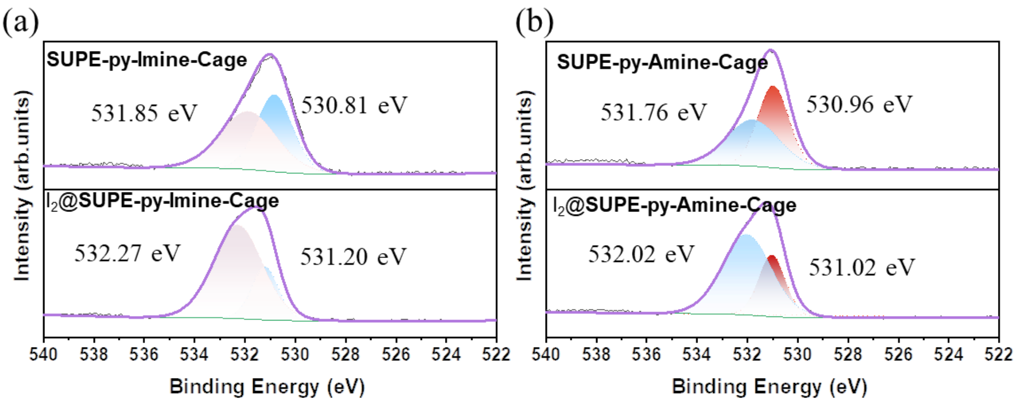

Supplementary Fig. 17 | XPS spectra of *O 1s* for (a) **SUPE–py–Imine–Cage** and (b) **SUPE–py–Amine–Cage** before and after exposure to iodine vapor for 36 h.

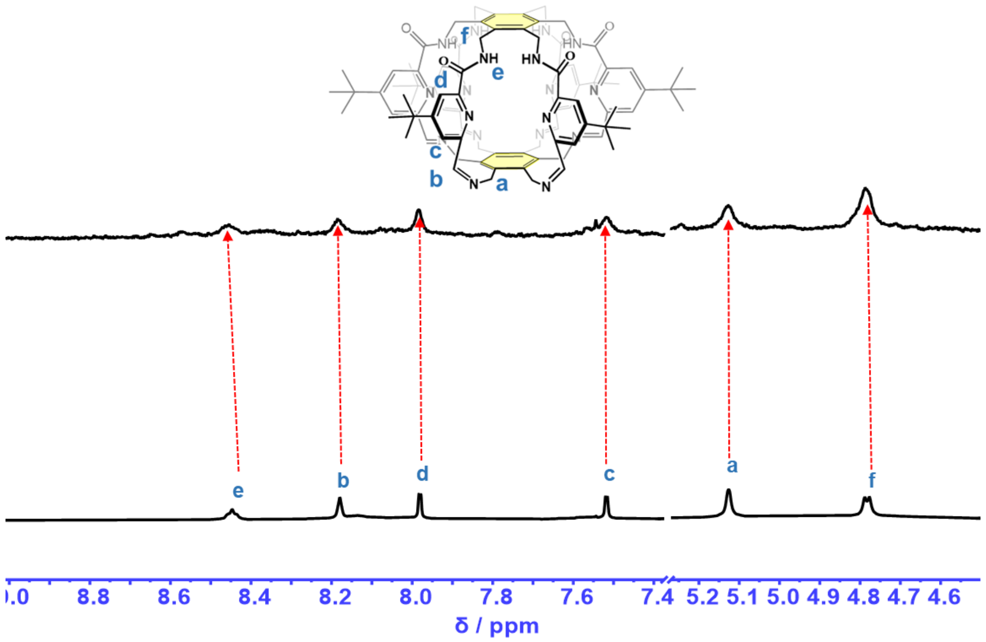

Supplementary Fig. 18 | Partial ^{1}H NMR spectra of a 2.0 mM solution of **SUPE–py–Imine–Cage** in the presence of 0 equiv. (bottom) and 10 equiv. (top) of I_2 in $CDCl_3$.

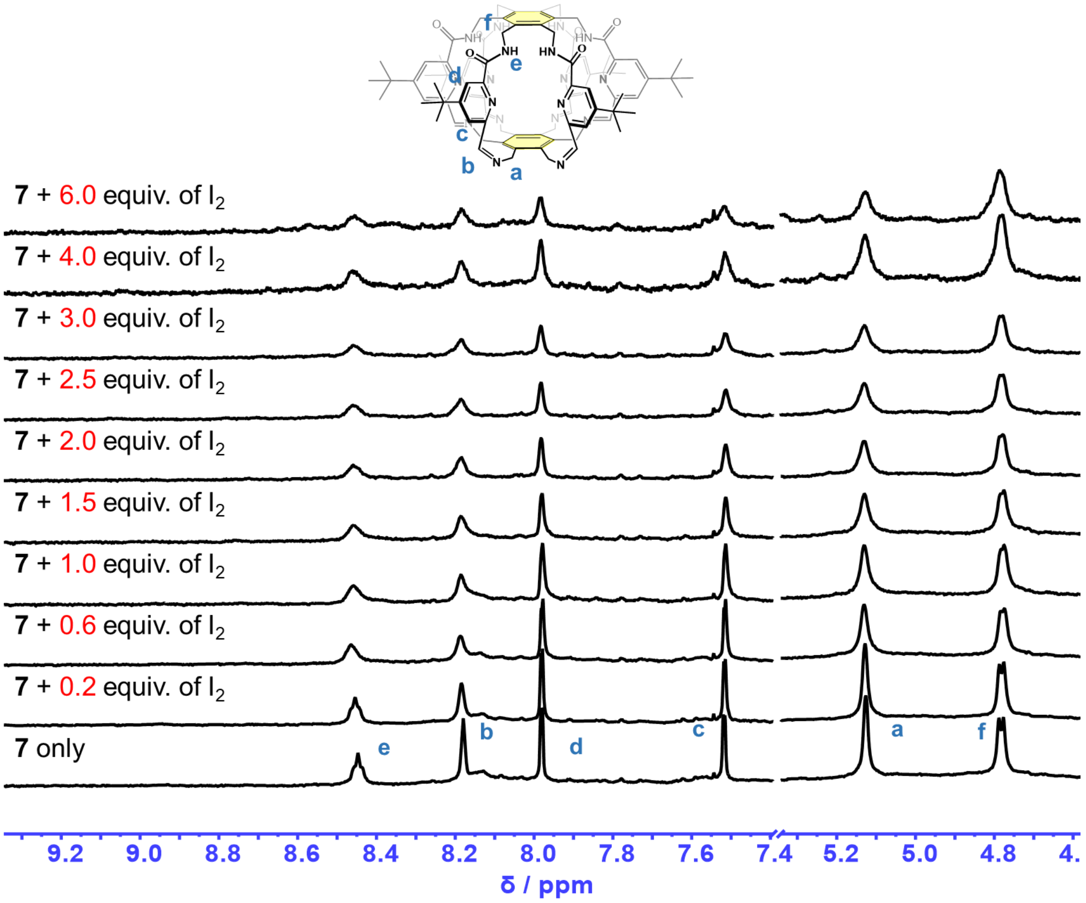

Supplementary Fig. 19 | ^{1}H NMR spectroscopic titrations of **SUPE–py–Imine–Cage (7)** (2.0 mM, 500 μL) with I_2 carried out by adding small aliquots of a solution of I_2 in $CDCl_3$ (200 mM) into a solution of the host in the same solvent using a microsampler.

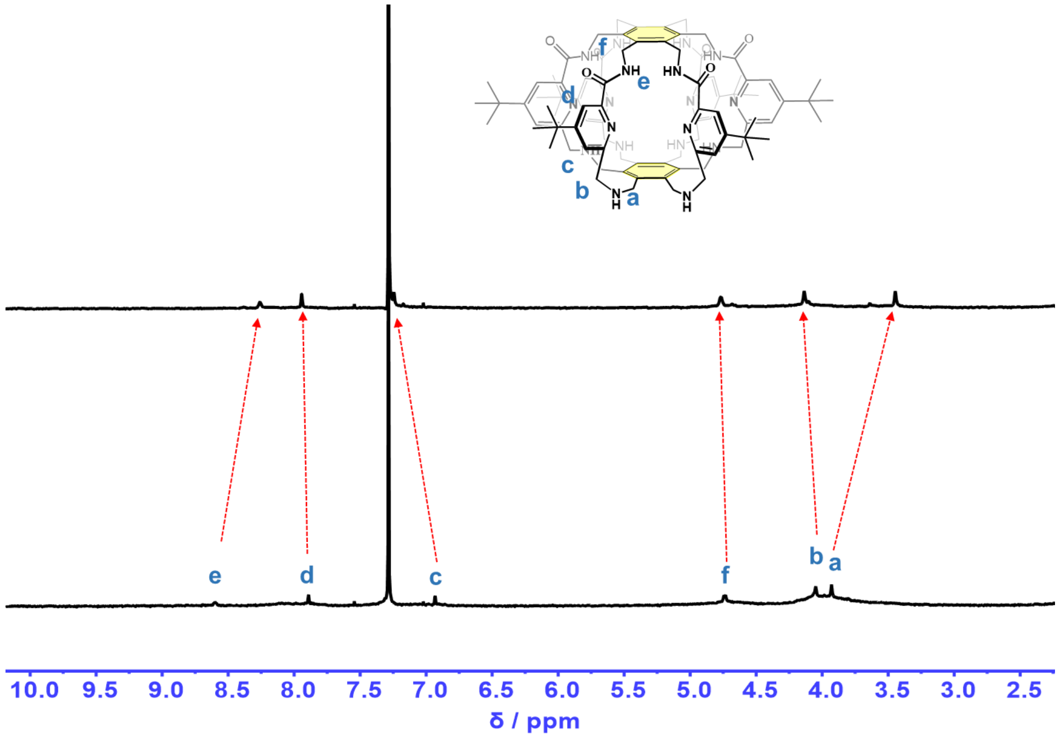

Supplementary Fig. 20 | Partial ^{1}H NMR spectra of a 2.0 mM solution of **SUPE–py–Amine–Cage** in the absence (bottom) and presence (top) of 10 equiv. of I_2 (top) in $CDCl_3$.

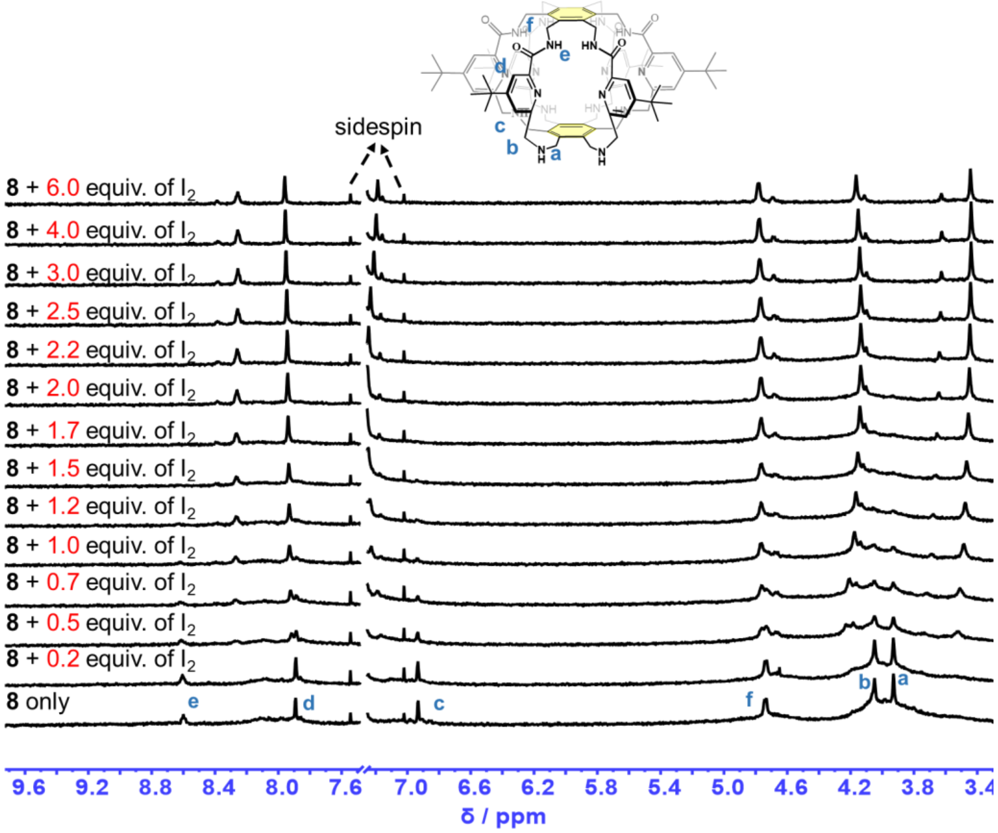

Supplementary Fig. 21 | ^{1}H NMR spectroscopic titrations of **SUPE–py–Amine–Cage (8)** (2.0 mM, 500 μL) with I_2 carried out by adding small aliquots of a solution of I_2 in $CDCl_3$ (200 mM) into a solution of the host in the same solvent using a microsampler.

Supplementary Note 3. Static iodine adsorption from contaminated water

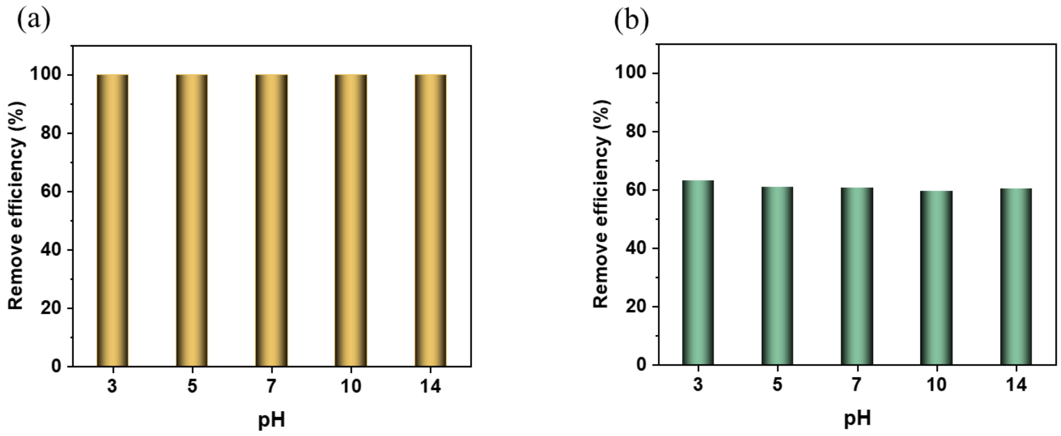

Supplementary Fig. 22 | The pH effect on aqueous iodine adsorption with (a) **SUPE–py–Amine–Cage** and (b) ACs.

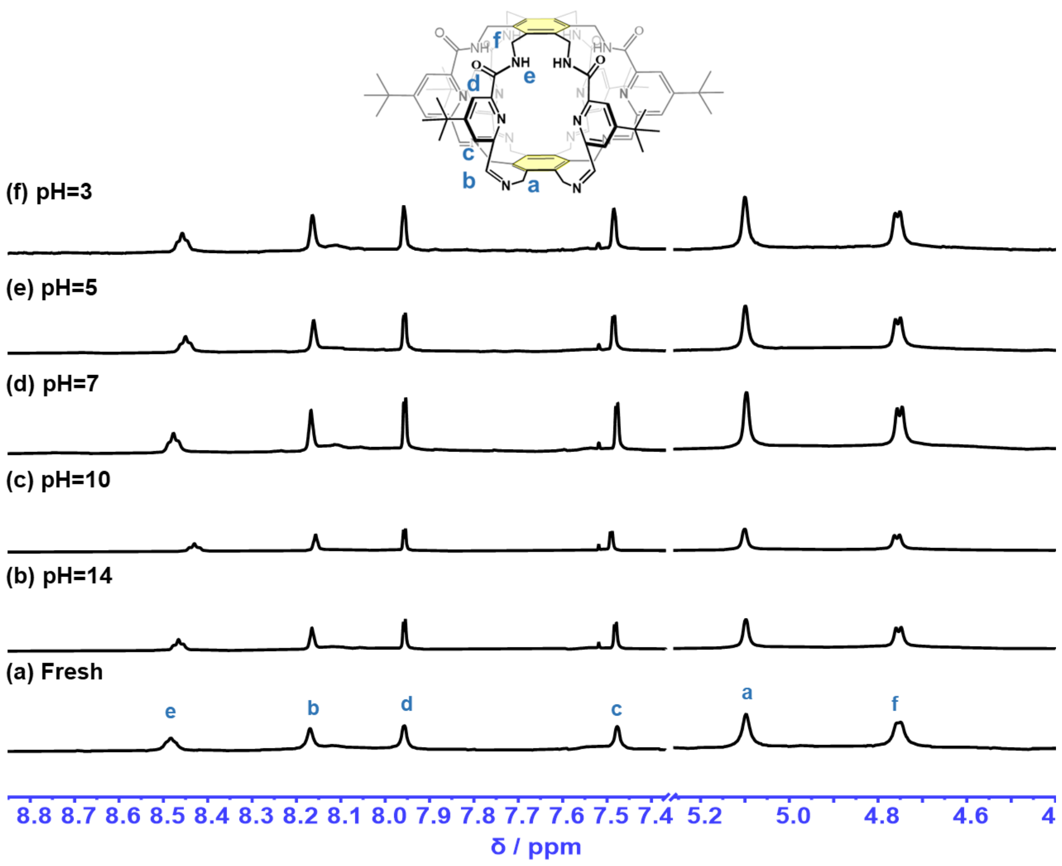

Supplementary Fig. 23 | ^{1}H NMR spectra of (a) fresh **SUPE–py–Imine–Cage** and recycled **SUPE–py–Imine–Cage** after immersing in differnt pH water for 24 h: (b) pH=14, (c) pH=10, (d) pH=7, (e) pH=5 and (f) pH=3 in $CDCl_3$.

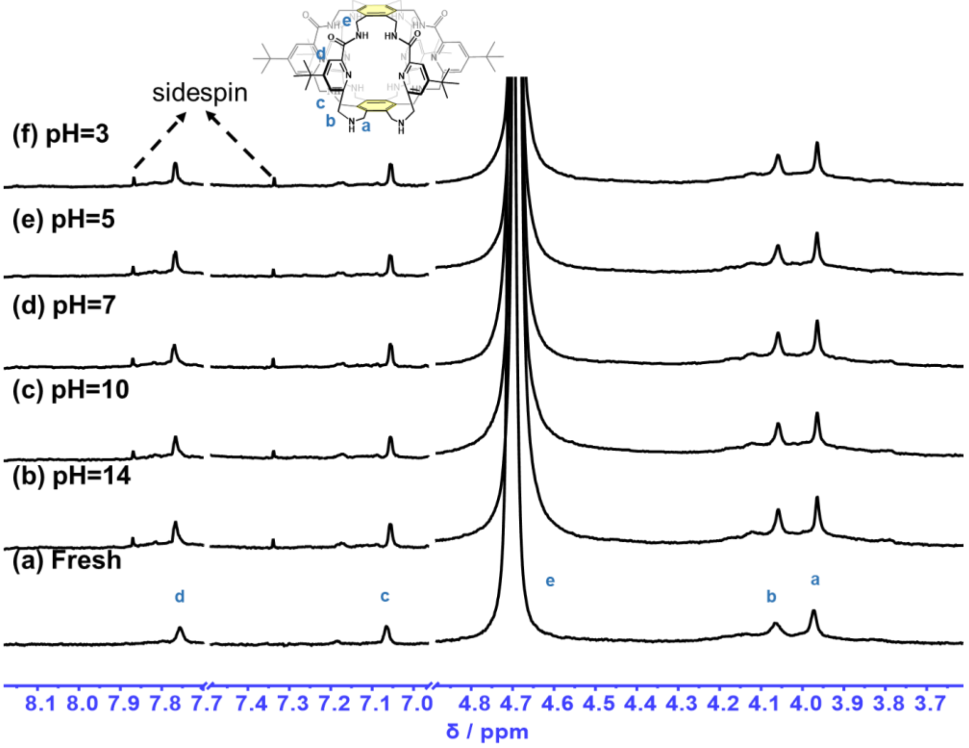

Supplementary Fig. 24 | ^{1}H NMR spectroscopy of a mixed solvent consisting of $CDCl_3$/CD_3OD (1/1, v/v) of (a) fresh **SUPE–py–Amine–Cage** and recycled **SUPE–py–Amine–Cage** after immersed in differnt pH water for 24 h, (b) pH=14, (c) pH=10, (d) pH=7, (e) pH=5 and (f) pH=3.

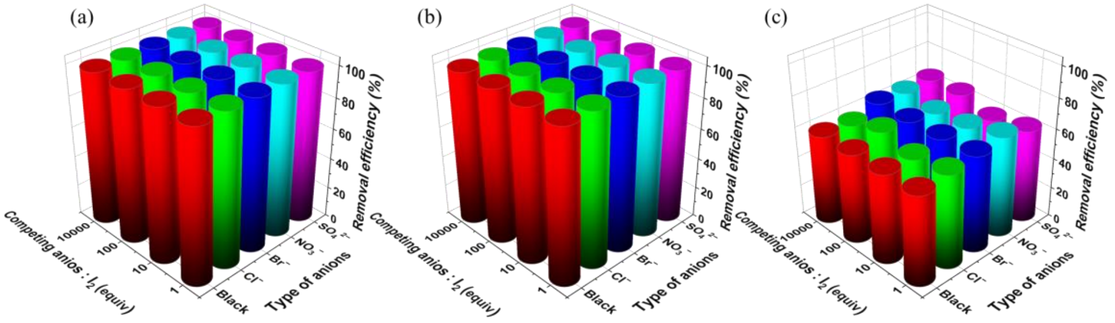

Supplementary Fig. 25 | Iodine adsorption from aqueous solutions containing an excess (1 to 10000 equivalents) of a single competing anion, viz. Cl^-, Br^-, NO_3^-, or SO_4^{2-}, with (a) **SUPE–py–Imine–Cage** , (b) **SUPE–py–Amine–Cage** and (c) ACs. Note: the I_2 concentration is 1.0 mM for the presence of 1–100 equivalents of competing anions and 0.5 mM for the presence of 10000 equivalents of competing anions.

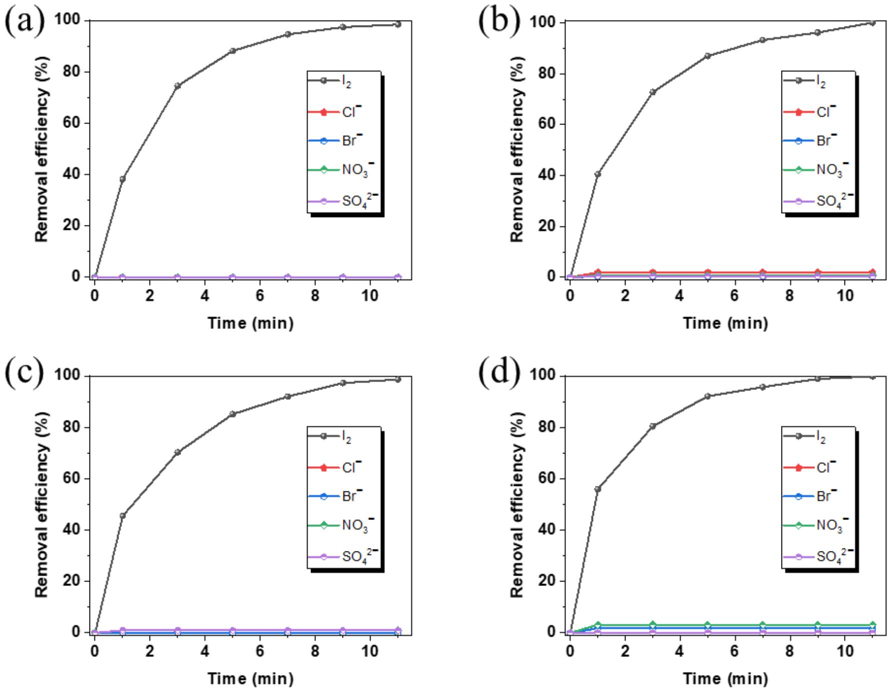

Supplementary Fig. 26 | Iodine adsorption from aqueous solutions containing 1 equivalent of equal–molar competing anions, viz. Cl^-, Br^-, NO_3^-, and SO_4^{2-}, with **SUPE–py–Imine–Cage** at different pH: (a) pH=3, (b) pH=5, (c) pH=7 and (d) pH=10.

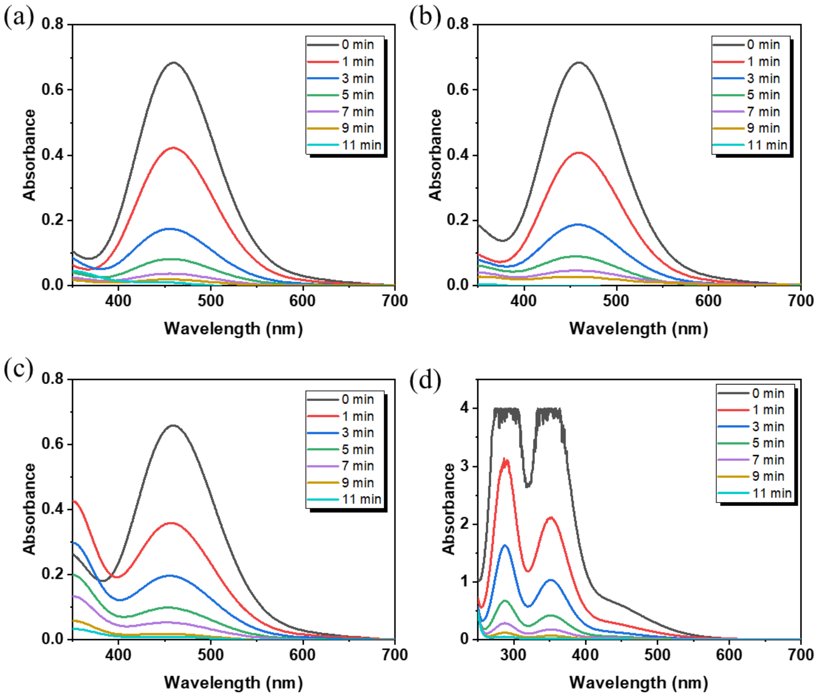

Supplementary Fig. 27 | UV/vis spectra of iodine adsorption from aqueous solutions containing 1 equivalent of equal–molar competing anions, viz. Cl^-, Br^-, NO_3^-, and SO_4^{2-}, with **SUPE–py–Imine–Cage** at different pH: (a) pH=3, (b) pH=5, (c) pH=7 and (d) pH=10.

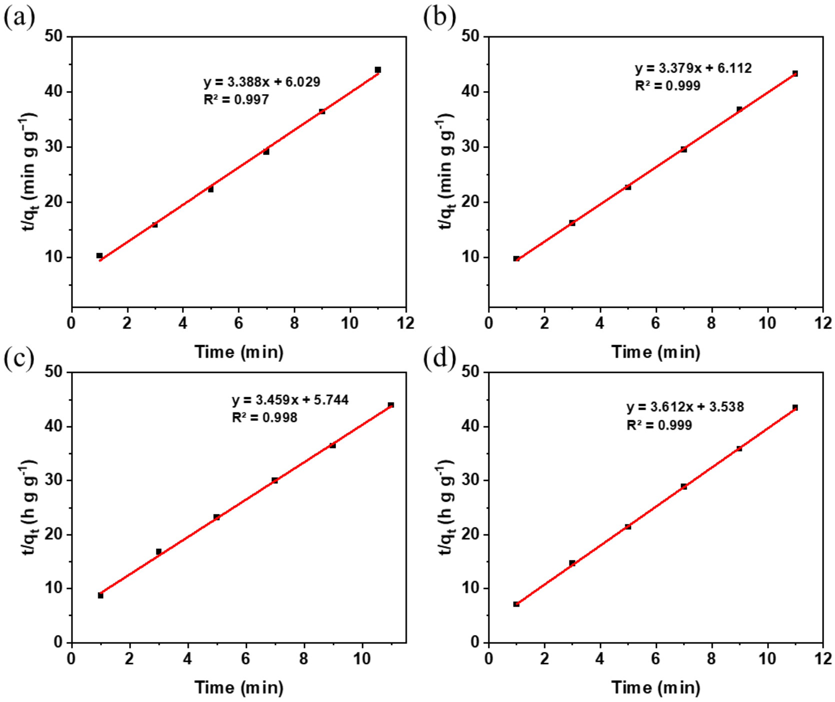

Supplementary Fig. 28 | Pseudo–second order kinetic model of iodine adsorption from aqueous solutions containing 1 equivalent of equal–molar competing anions, viz. Cl^-, Br^-, NO_3^-, and SO_4^{2-}, with **SUPE–py–Imine–Cage** at different pH: (a) pH=3, (b) pH=5, (c) pH=7 and (d) pH=10. These data were derived from Supplementary Fig. 27.

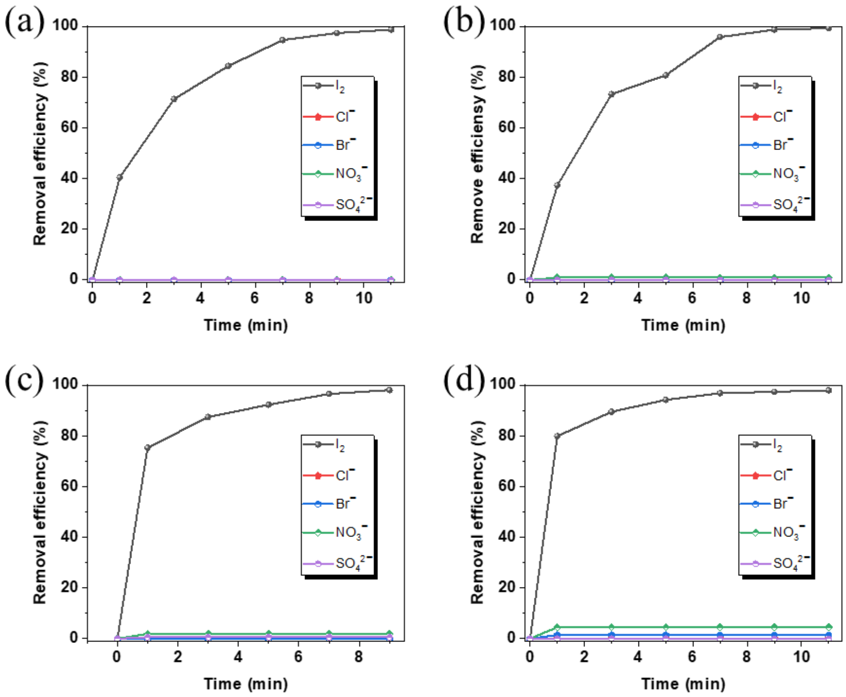

Supplementary Fig. 29 | Iodine adsorption from aqueous solutions containing 1 equivalent of equal–molar competing anions, viz. Cl^-, Br^-, NO_3^-, and SO_4^{2-}, with **SUPE–py–Amine–Cage** at different pH: (a) pH=3, (b) pH=5, (c) pH=7 and (d) pH=10.

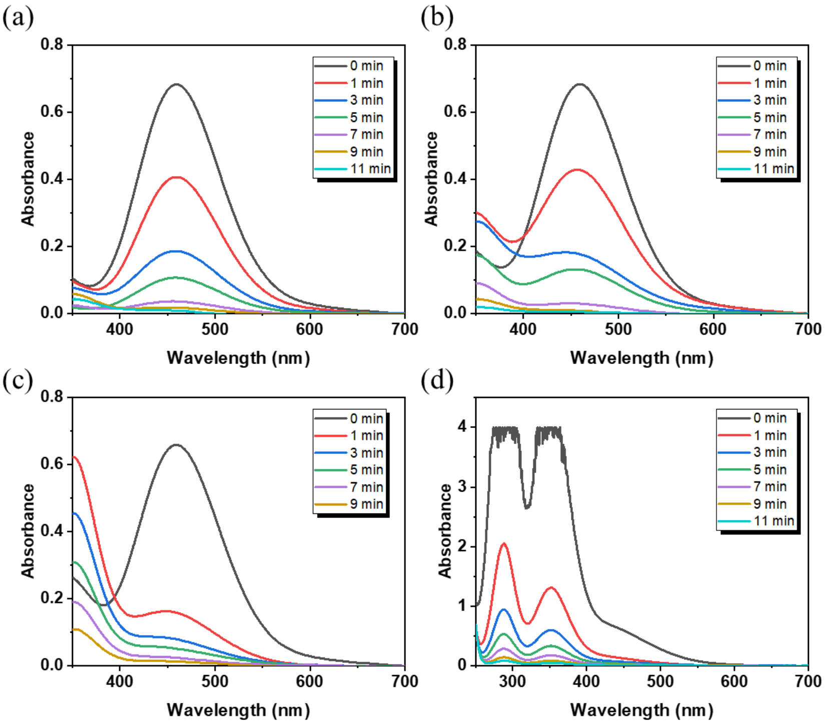

Supplementary Fig. 30 | UV/vis spectra of iodine adsorption from aqueous solutions containing 1 equivalent of equal–molar competing anions, viz. Cl^-, Br^-, NO_3^-, and SO_4^{2-}, with **SUPE–py–Amine–Cage** at different pH: (a) pH=3, (b) pH=5, (c) pH=7 and (d) pH=10.

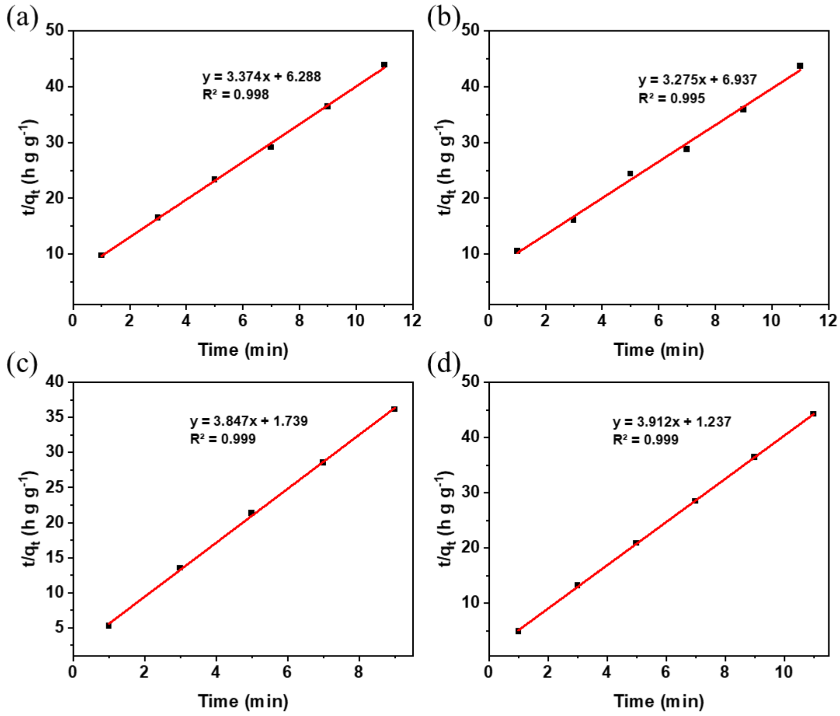

Supplementary Fig. 31 | Pseudo–second order kinetic model of iodine adsorption from aqueous solutions containing 1 equivalent of equal–molar competing anions, viz. Cl^-, Br^-, NO_3^-, and SO_4^{2-}, with **SUPE–py–Amine–Cage** at different pH: (a) pH=3, (b) pH=5, (c) pH=7 and (d) pH=10. These data were derived from Supplementary Fig. 30.

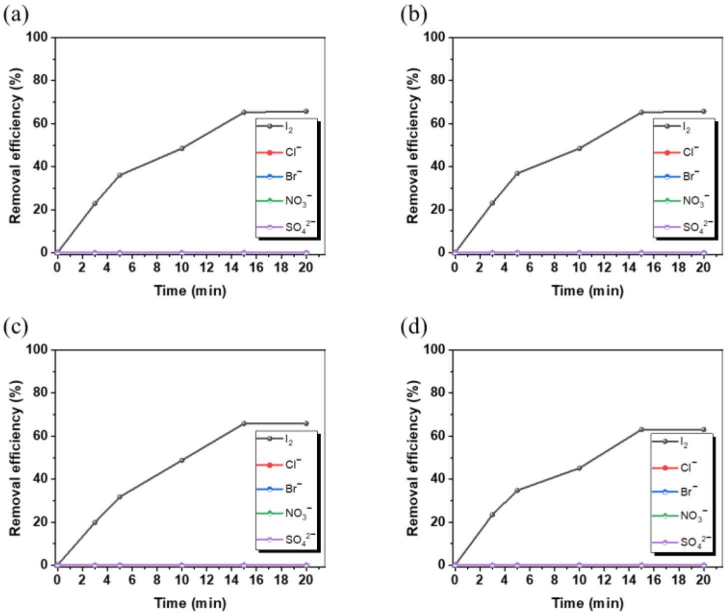

Supplementary Fig. 32 | Iodine adsorption from aqueous solutions containing 1 equivalent of equal–molar competing anions, viz. Cl^{-}, Br^{-}, NO_3^{-}, and SO_4^{2-}, with ACs at different pHs: (a) pH=3, (b) pH=5, (c) pH=7 and (d) pH=10.

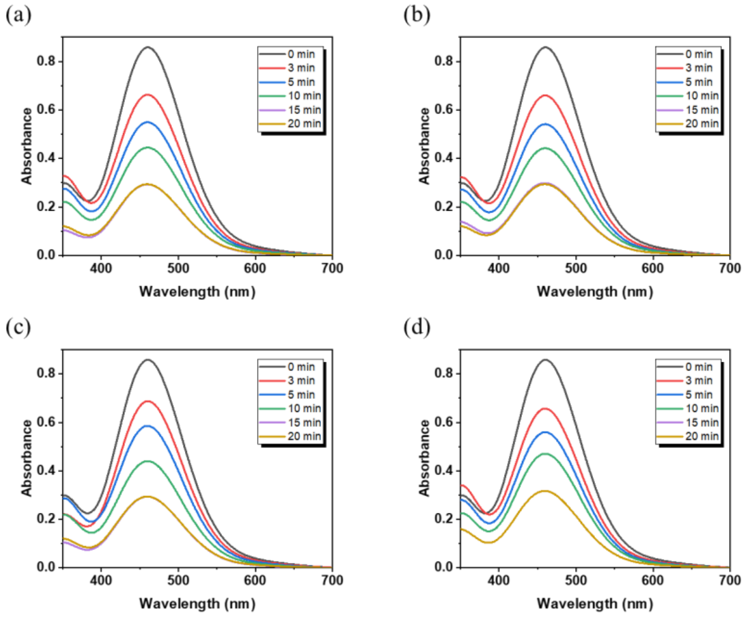

Supplementary Fig. 33 | UV/vis spectra of iodine adsorption from aqueous solutions containing 1 equivalent of equal–molar competing anions, viz. Cl^{-}, Br^{-}, NO_3^{-}, and SO_4^{2-}, with ACs at different pH: (a) pH=3, (b) pH=5, (c) pH=7 and (d) pH=10.

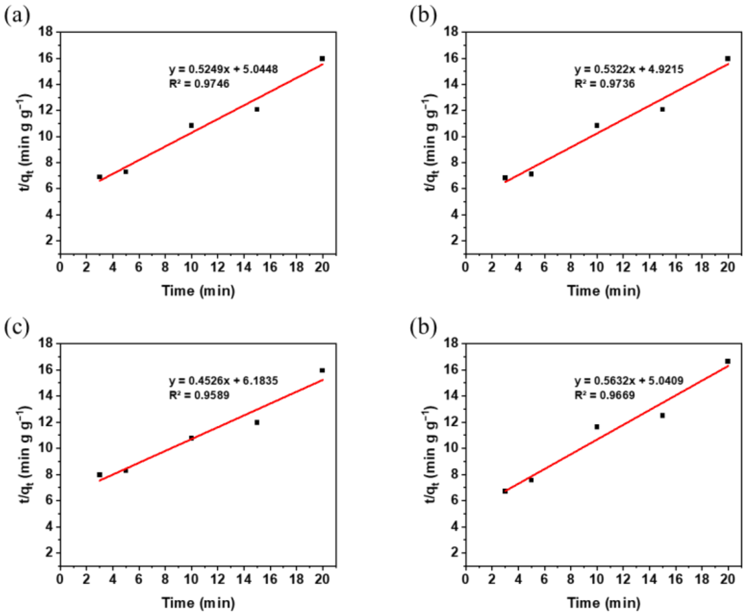

Supplementary Fig. 34| Pseudo–second order kinetic model of iodine adsorption from aqueous solutions containing 1 equivalent of equal–molar competing anions, viz. Cl^-, Br^-, NO_3^-, and SO_4^{2-}, with ACs at different pH: (a) pH=3, (b) pH=5, (c) pH=7 and (d) pH=10. These data were derived from Supplementary Fig. 33.

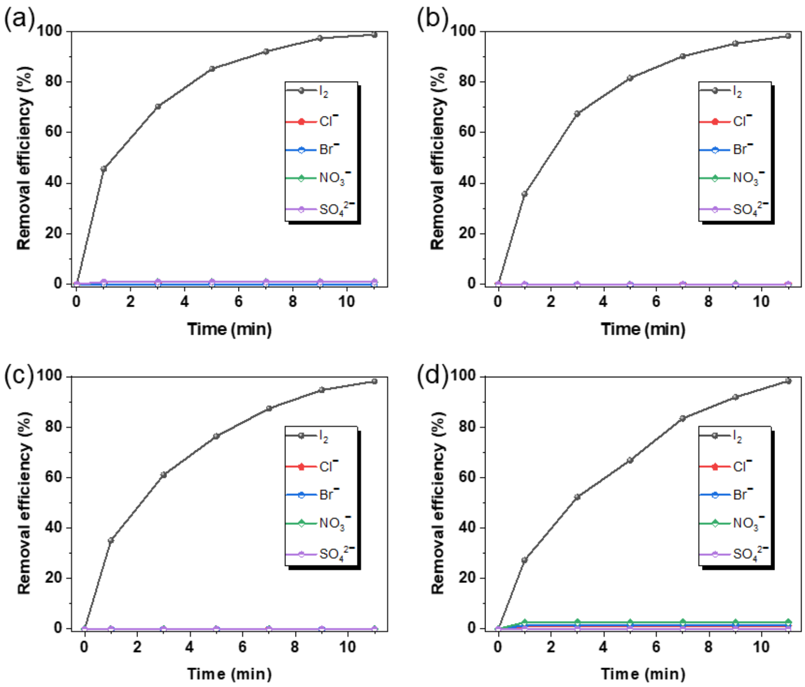

Supplementary Fig. 35 | Iodine adsorption from aqueous solutions containing an excess (1 to 1000 equivalents) of co–existing competing anions (equal–molar Cl^-, Br^-, NO_3^-, and SO_4^{2-}) with **SUPE–py–Imine–Cage**: (a) 1 equivalent, (b) 10 equivalents, (c) 100 equivalents, and (d) 1000 equivalents. Note: the I_2 concentration is 1.0 mM for the presence of 1–100 equivalents of competing anions and 0.5 mM for the presence of 1000 equivalents of competing anions.

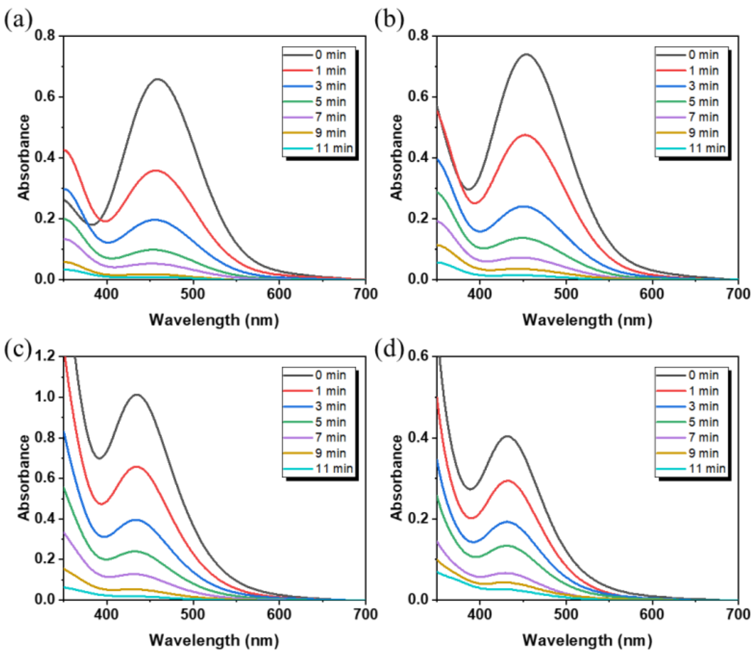

Supplementary Fig. 36 | UV/vis spectra of iodine adsorption from aqueous solutions containing an excess (1 to 1000 equivalents) of co–existing competing anions (equal–molar Cl^-, Br^-, NO_3^-, and SO_4^{2-}) with **SUPE–py–Imine–Cage**: (a) 1 equivalent, (b) 10 equivalents, (c) 100 equivalents, and (d) 1000 equivalents. Note: the I_2 concentration is 1.0 mM for the presence of 1–100 equivalents of competing anions and 0.5 mM for the presence of 1000 equivalents of competing anions.

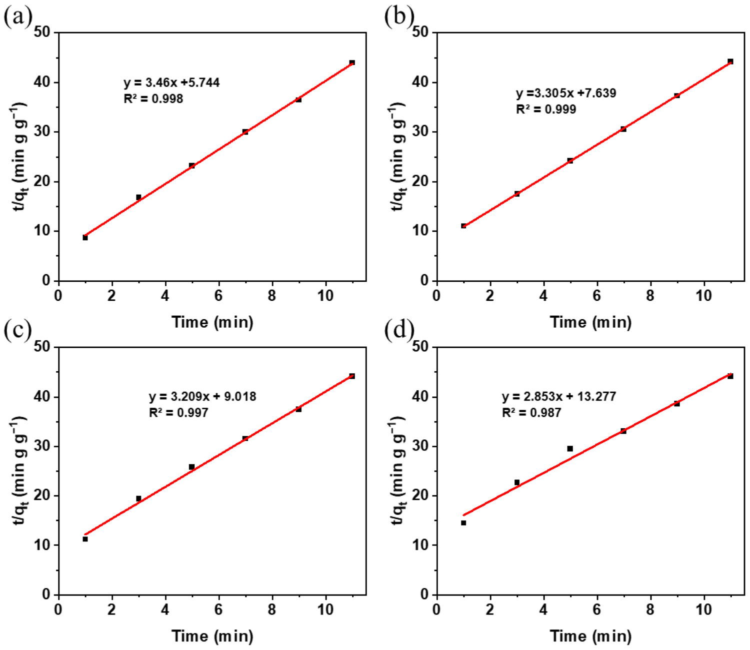

Supplementary Fig. 37 |Pseudo–second order kinetic model of iodine adsorption from aqueous solutions containing an excess (1 to 1000 equivalents) of co–existing competing anions (equal–molar Cl^-, Br^-, NO_3^-, and SO_4^{2-}) with **SUPE–py–Imine–Cage**: (a) 1 equivalent, (b) 10 equivalents, (c) 100 equivalents, and (d) 1000 equivalents. These data were derived from Supplementary Fig.36. Note: the I_2 concentration is 1.0 mM for the presence of 1–100 equivalents of competing anions and 0.5 mM for the presence of 1000 equivalents of competing anions.

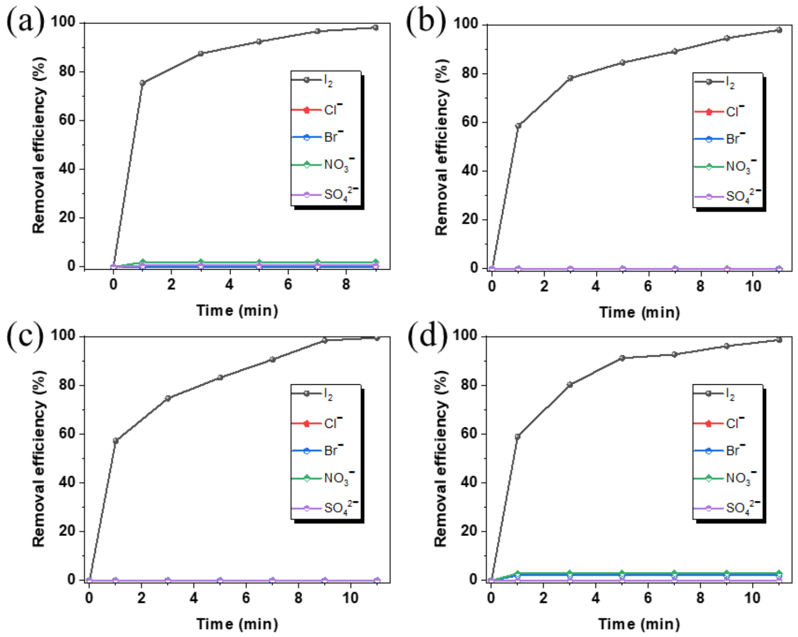

Supplementary Fig. 38 | Iodine adsorption from aqueous solutions containing an excess (1 to 1000 equivalents) of co–existing competing anions (equal–molar Cl^-, Br^-, NO_3^-, and SO_4^{2-}) with **SUPE–py–Amine–Cage**: (a) 1 equivalent, (b) 10 equivalents, (c) 100 equivalents, and (d) 1000 equivalents. Note: the I_2 concentration is 1.0 mM for the presence of 1–100 equivalents of competing anions and 0.5 mM for the presence of 1000 equivalents of competing anions.

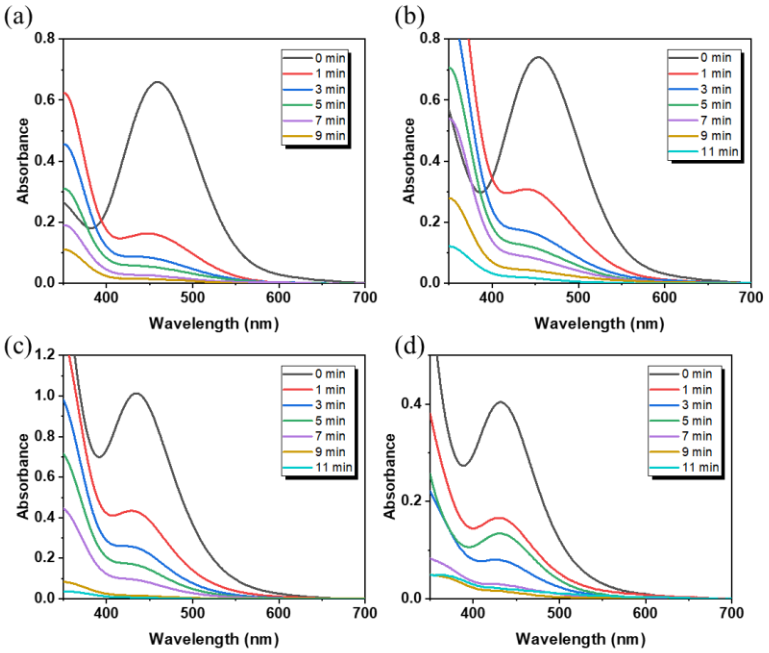

Supplementary Fig. 39 | UV/vis spectra of iodine adsorption from aqueous solutions an excess (1 to 1000 equivalents) of co–existing competing anions (equal–molar Cl^-, Br^-, NO_3^-, and SO_4^{2-}) with **SUPE–py–Amine–Cage**: (a) 1 equivalent, (b) 10 equivalents, (c) 100 equivalents, and (d) 1000 equivalents. Note: the I_2 concentration is 1.0 mM for the presence of 1–100 equivalents of competing anions and 0.5 mM for the presence of 1000 equivalents of competing anions.

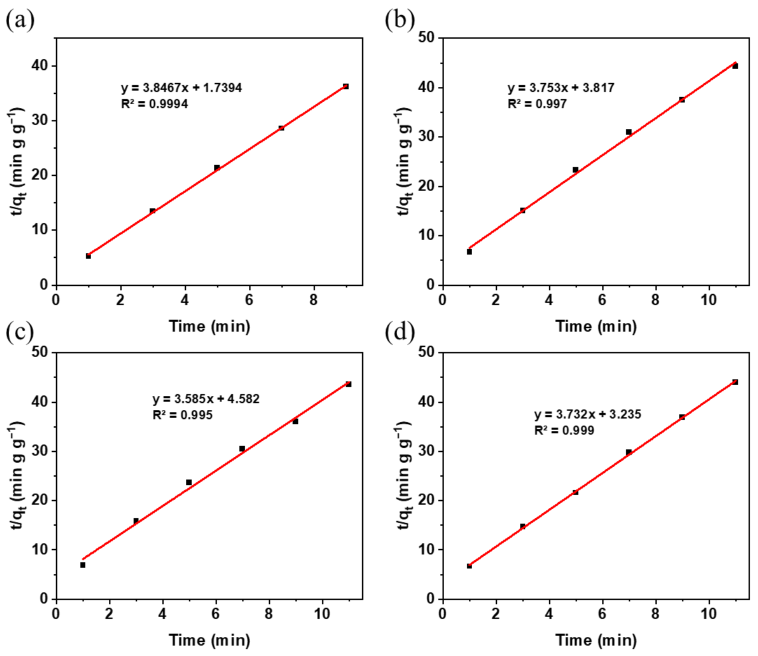

Supplementary Fig. 40 | Pseudo–second order kinetic model of iodine adsorption from aqueous solutions containing an excess (1 to 1000 equivalents) of co–existing competing anions (equal–molar Cl^-, Br^-, NO_3^-, and SO_4^{2-}) with **SUPE–py–Amine–Cage**: (a) 1 equivalent, (b) 10 equivalents, (c) 100 equivalents, and (d) 1000 equivalents. These data were derived from Supplementary Fig. 39. Note: the I_2 concentration is 1.0 mM for the presence of 1–100 equivalents of competing anions and 0.5 mM for the presence of 1000 equivalents of competing anions.

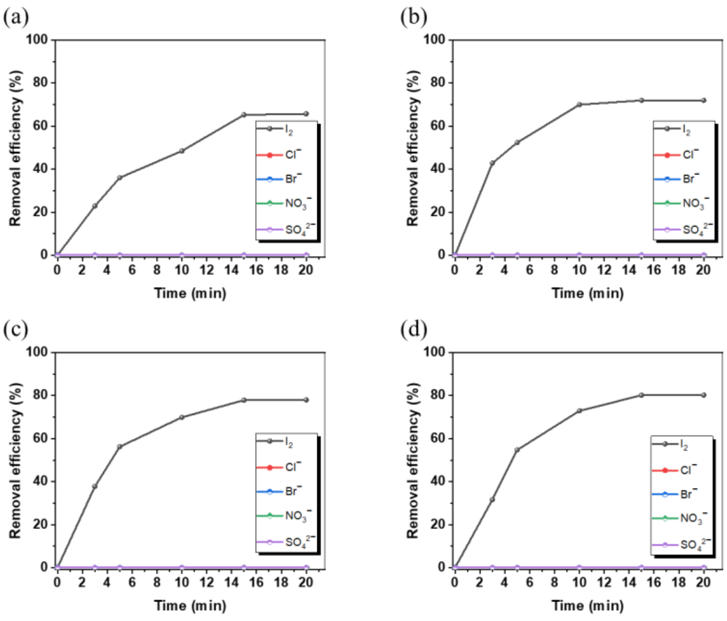

Supplementary Fig. 41 | Iodine adsorption from aqueous solutions containing an excess (1 to 1000 equivalents) of co–existing competing anions (equal–molar Cl^-, Br^-, NO_3^-, and SO_4^{2-}) with ACs: (a) 1 equivalent, (b) 10 equivalents, (c) 100 equivalents, and (d) 1000 equivalents. Note: the I_2 concentration is 1.0 mM for the presence of 1–100 equivalents of competing anions and 0.5 mM for the presence of 1000 equivalents of competing anions.

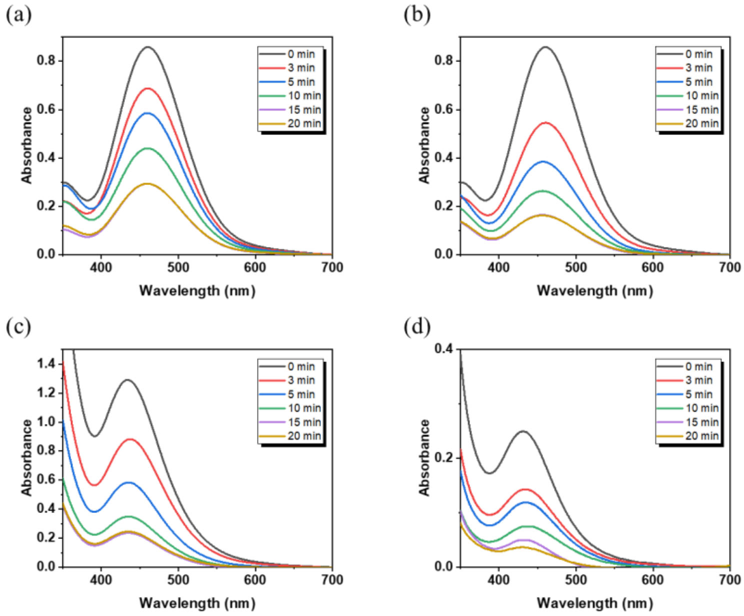

Supplementary Fig. 42 | UV/vis spectra of iodine adsorption from aqueous solutions an excess (1 to 1000 equivalents) of co–existing competing anions (equal–molar Cl^{-}, Br^{-}, NO_3^{-}, and SO_4^{2-}) with ACs: (a) 1 equivalent, (b) 10 equivalents, (c) 100 equivalents, and (d) 1000 equivalents. Note: the I_2 concentration is 1.0 mM for the presence of 1–100 equivalents of competing anions and 0.5 mM for the presence of 1000 equivalents of competing anions.

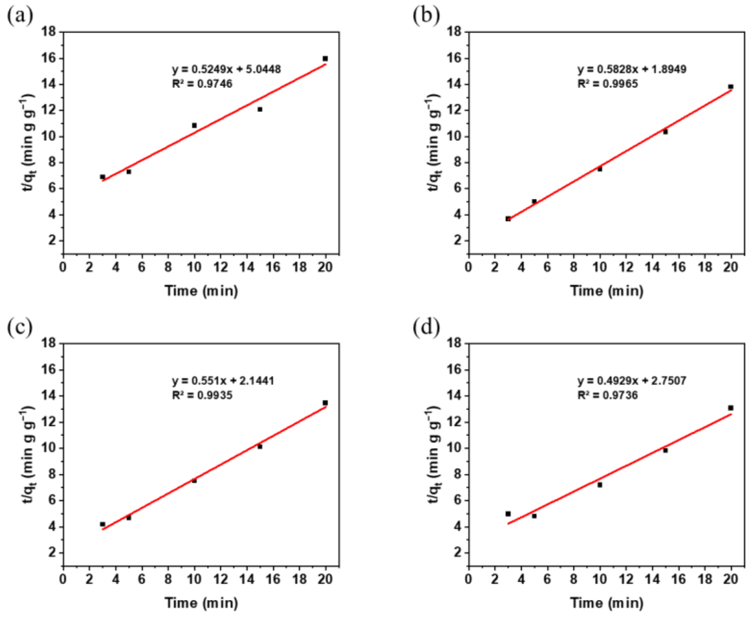

Supplementary Fig. 43 | Pseudo–second order kinetic model of iodine adsorption from aqueous solutions containing an excess (1 to 1000 equivalents) of co–existing competing anions (equal–molar Cl^{-}, Br^{-}, NO_3^{-}, and SO_4^{2-}) with ACs: (a) 1 equivalent, (b) 10 equivalents, (c) 100 equivalents, and (d) 1000 equivalents. These data were derived from Supplementary Fig. 42. Note: the I_2 concentration is 1.0 mM for the presence of 1–100 equivalents of competing anions and 0.5 mM for the presence of 1000 equivalents of competing anions.

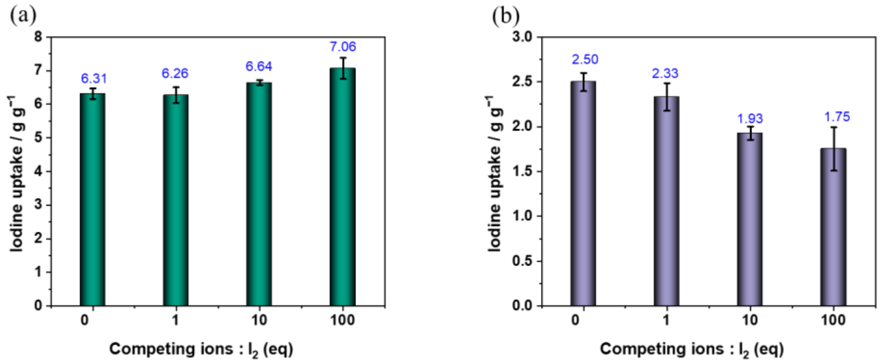

Supplementary Fig. 44 | Iodine adsorption capability from aqueous solutions containing an excess (0 to 100 equivalents) of co–existing competing anions (equal–molar Cl^-, Br^-, NO_3^-, and SO_4^{2-}) with (a) **SUPE–py–Amine–Cage** and (b) ACs. Concentration of I_2: 1.2 mM. Error bars represent SD. n = 3 independent experiments.

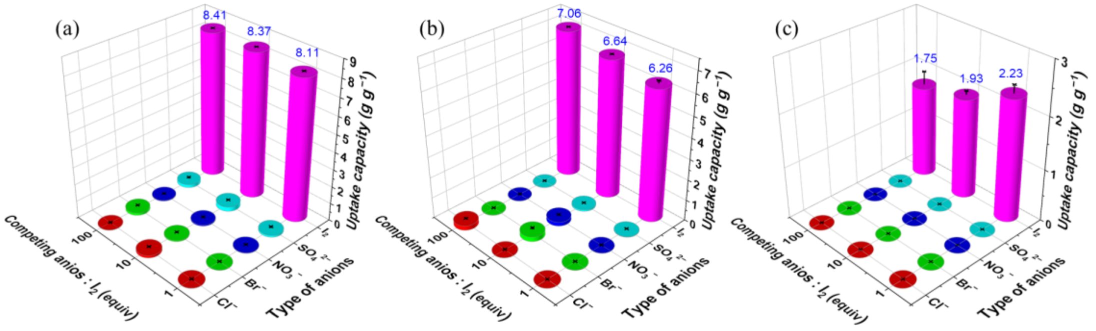

Supplementary Fig. 45 | Iodine adsorption capability and selectivity from aqueous solutions containing an excess (1 to 100 equivalents) of co–existing competing anions (equal–molar Cl^-, Br^-, NO_3^-, and SO_4^{2-}) with (a) **SUPE–py–Imine–Cage**, (b) **SUPE–py–Amine–Cage** and (c) ACs. Concentration of I_2: 1.2 mM. Error bars represent SD. n = 3 independent experiments.

Supplementary Table 1 | The maximum uptake capacity of I_2 in a various ratio of competing anions liquid phases.

Competing anions： I_2 （equiv）	Sorbents used	Uptake amount / g g^{-1}				
		I_2	Cl^-	Br^-	NO_3^-	SO_4^{2-}
1	7	8.11±0.04	0.01±0.01	0.008±0.007	0.02±0.01	0.1±0.07
	8	6.26 ±0.20	0.001±0.001	< 0.001	< 0.001	0.016±0.012
10	7	8.37 ±0.01	0.14±0.05	0.06±0.05	0.06±0.05	0.26±0.02
	8	6.64 ±0.02	< 0.001	0.23±0.08	0.19±0.02	0.07±0.005
100	7	8.41 ±0.01	0.013±0.012	0.14±0.05	0.004±0.001	0.18±0.07
	8	7.06 ±0.01	0.22±0.004	< 0.001	< 0.001	< 0.001

Note: 7: SUPE–py–Imine–Cage, and 8: SUPE–py–Amine–Cage.

Supplementary Table 2 | Comparison of maximum I_2 uptake capacity from liquid phases.

Adsorbents	Category	Hours (h)	Capacity (g g^{-1})	Ref.
SUPE–py–Imine–Cage	NAS	48	8.41	This work
SUPE–py–Amine–Cage	NAS	48	7.06	This work
COFA–1	COF	12	6.78	4
COFP–1	COF	12	5.05	4
C[4]P–BTP	POP	48	3.03	5
C[4]P–TPE	POP	48	2.80	5
ACs	ACM	48	2.5	This work
C[4]P–BP	POP	48	2.31	5
C[4]P–TTP	POP	48	2.22	5
Mg–Co–Al–LDH@g–C_3N_4	NM	0.5 (Sonication)	2.2	6
C[4]P–BT	POP	48	2.16	5
Fe_3O_4@PPy	NM	2	1.627	7
PTEP	COF	2	1.45	8
C[4]P–DPP	POP	48	1.35	5

Note: NM: Nanoscale materials; COF: covalent – organic framework; POP: porous organic polymers; ACM: active carbon materials; NAS: nonporous amorphous (superad)sorbents.

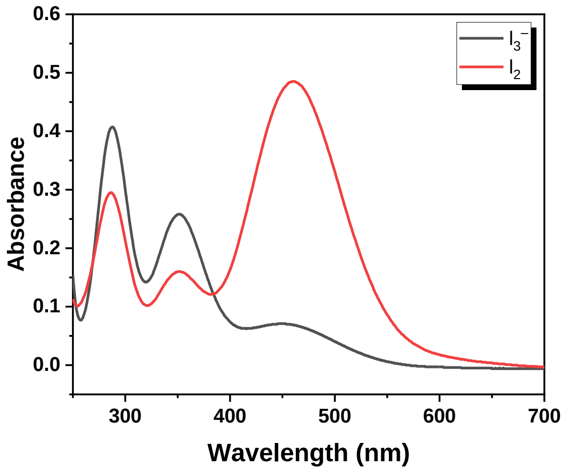

Supplementary Fig. 46 | UV–vis spectra of I_2 (in red, 0.6 mM) in water and I_3^- (in black, 1.5 mg I_2 + 3 mg KI in 100 mL of water).

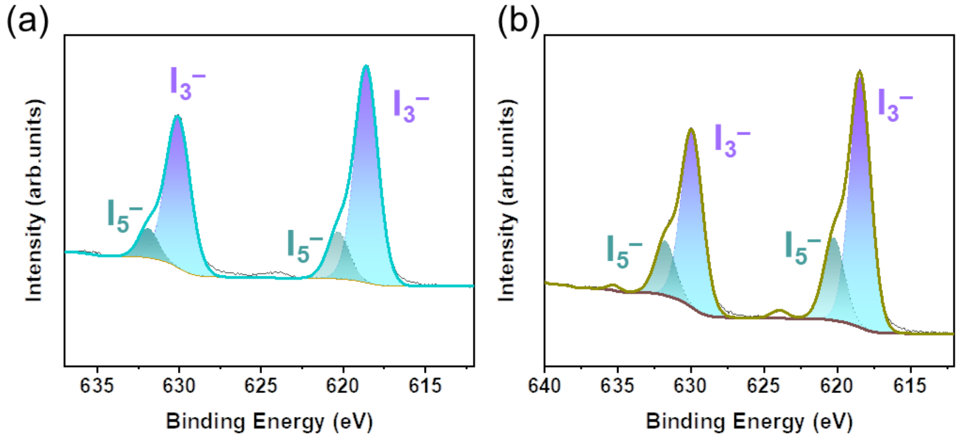

Supplementary Fig. 47 | XPS *I 3d* spectra of (a) I_3^-@**SUPE–py–Imine–Cage** and (b) I_3^-@**SUPE–py–Amine–Cage** derived signals.

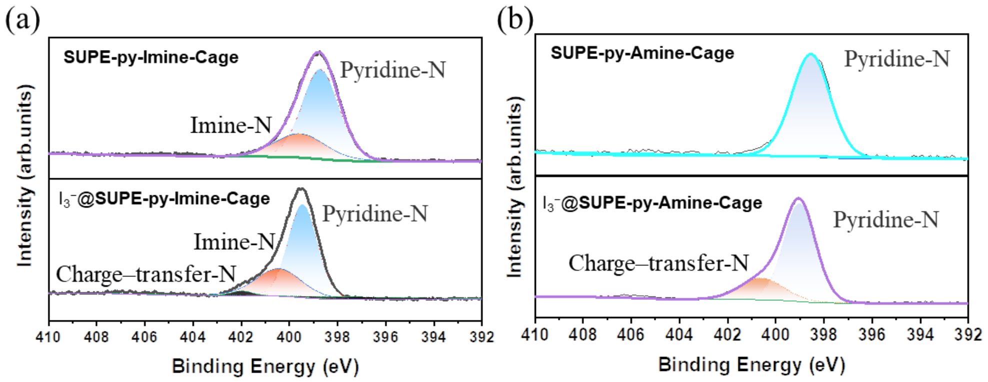

Supplementary Fig. 48 |XPS *N 1s* spectra of (a) **SUPE–py–Imine–Cage** and (b) **SUPE–py–Amine–Cage** before and after adsorption of iodine (I_2/KI).

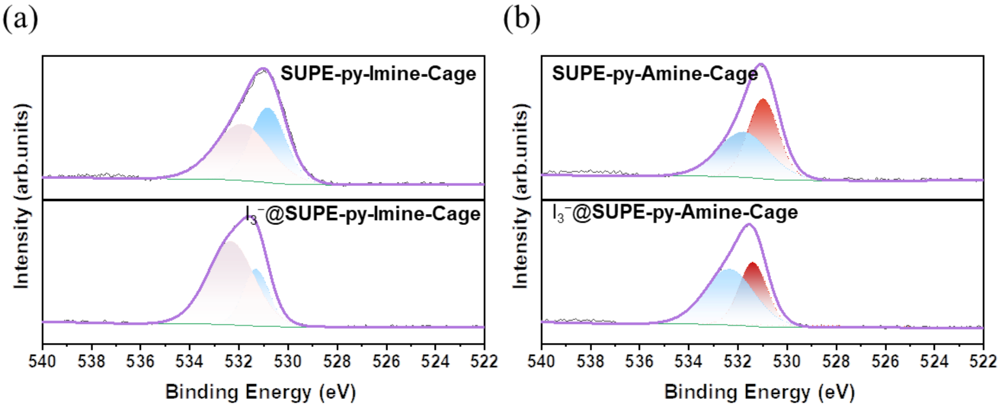

Supplementary Fig. 49 | XPS *O 1s* spectra of (a) **SUPE–py–Imine–Cage** and (b) **SUPE–py–Amine–Cage** before and after adsorption of iodine (I_2/KI).

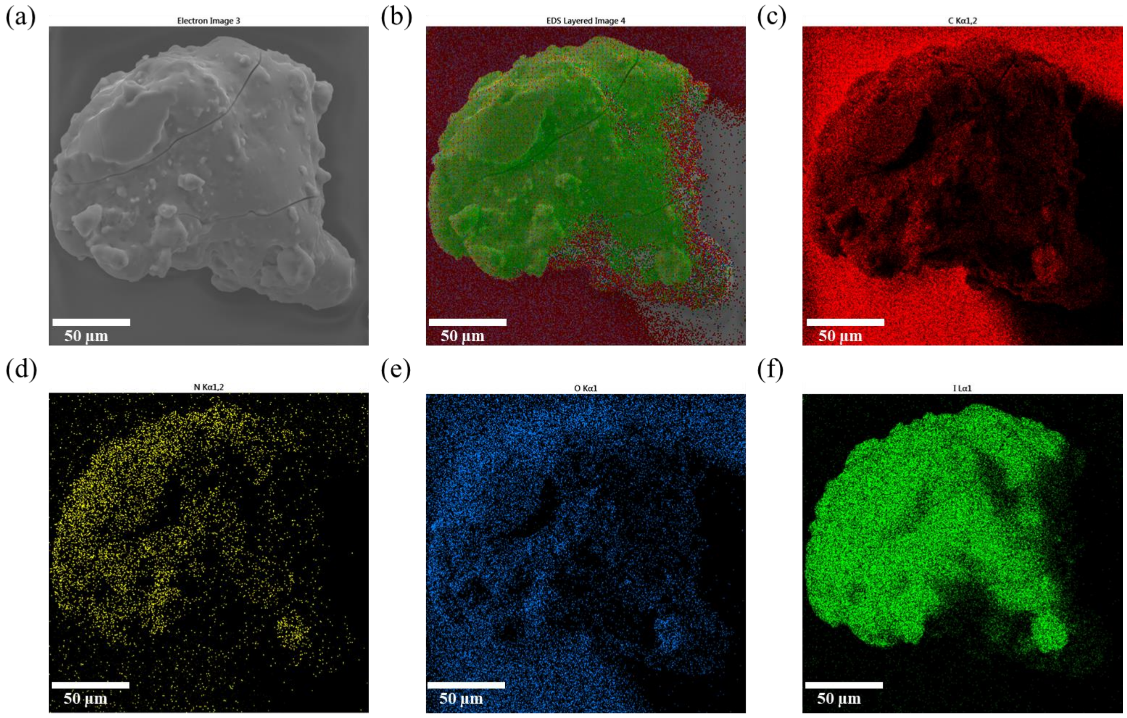

Supplementary Fig. 50 | SEM and EDS mapping results. (a) SEM image of I_3^-@**SUPE–py–Imine–Cage**, (b) EDS layered image and the SEM/EDS mapping for (c) C, (d) N, (e) O and (f) I.

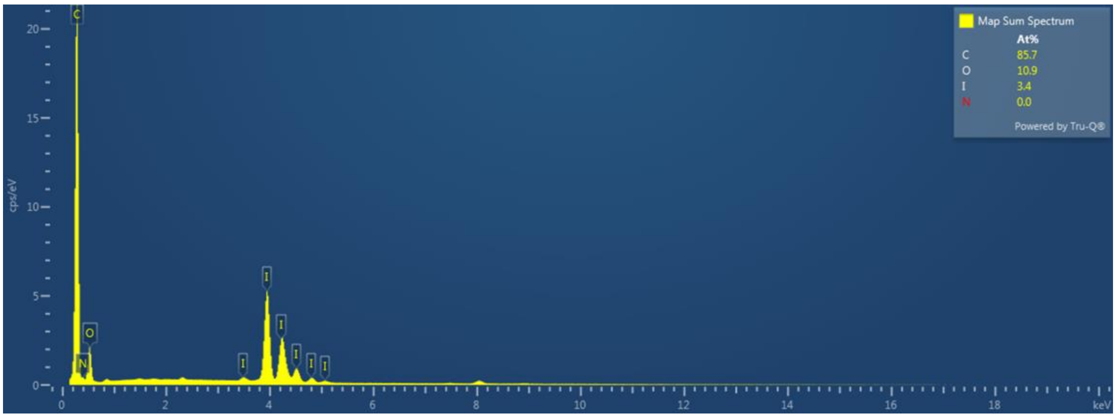

Supplementary Fig. 51 | SEM–EDS results of I_3^-@**SUPE–py–Imine–Cage**.

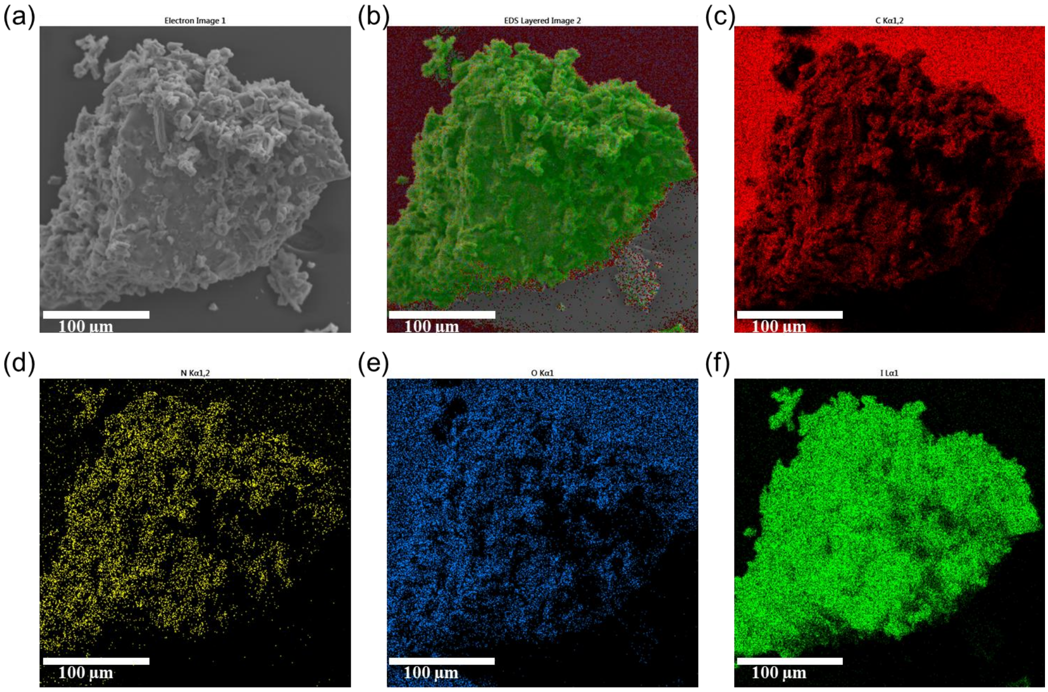

Supplementary Fig. 52 | SEM and EDS mapping results. (a) SEM image of I_3^-@**SUPE–py–Amine–Cage**, (b) EDS layered image and the SEM/EDS mapping for (c) C, (d) N, (e) O and (f) I.

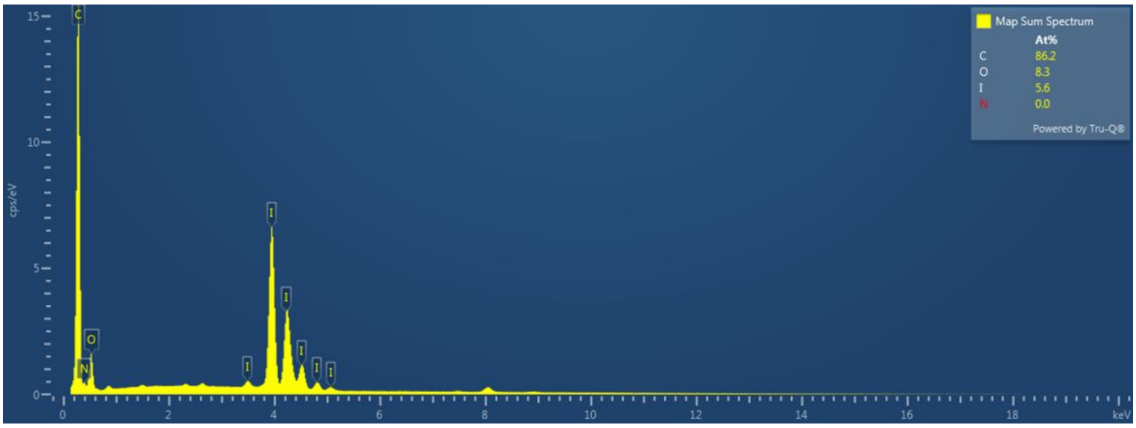

Supplementary Fig. 53 | SEM–EDS results of I_3^-@**SUPE–py–Amine–Cage**.

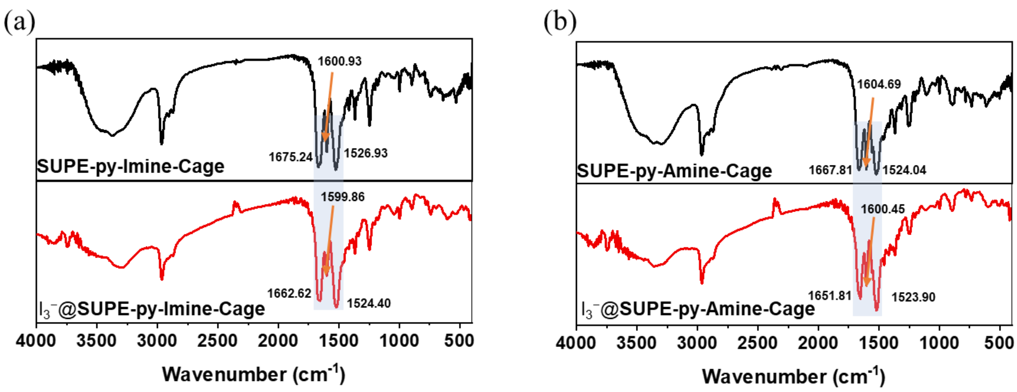

Supplementary Fig. 54 | FT–IR spectra of (a) **SUPE–py–Imine–Cage** and (b) **SUPE–py–Amine–Cage** before (in black) and after (in red) exposure to aqueous KI_3 (as KI/I_2) solutions for 24 h.

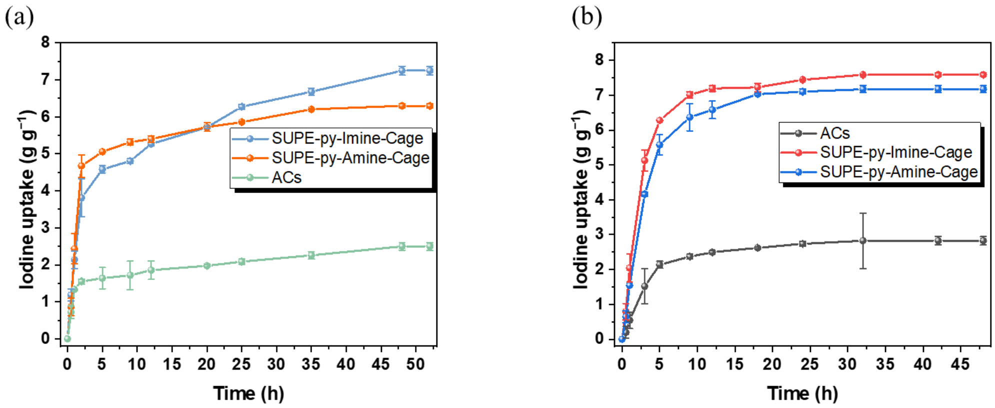

Supplementary Fig. 55 | Adsorption isotherm for static adsorption of (a) I_2 and (b) I_3^- (I_2/KI) in aqueous media with **SUPE–py–Imine–Cage**, **SUPE–py–Imine–Cage** and activated carbons (ACs).

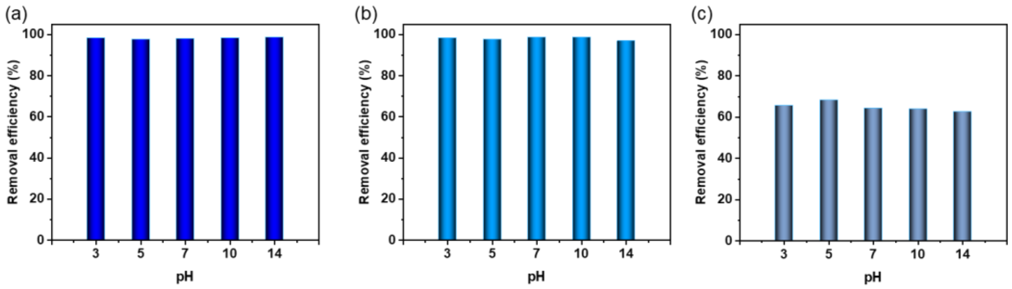

Supplementary Fig. 56 | The pH effect on aqueous I_3^- adsorption (iodine removal efficiency) with (a) **SUPE–py–Imine–Cage**, (b) **SUPE–py–Amine–Cage** and (c) ACs.

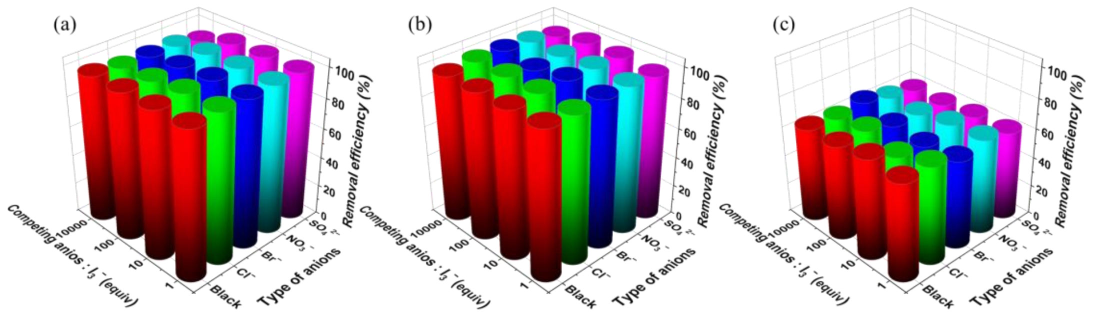

Supplementary Fig. 57 | Iodine adsorption from aqueous I_3^- solutions containing an excess (1 to 10000 equivalents) of a single competing anion, viz. Cl^-, Br^-, NO_3^-, or SO_4^{2-}, with (a) **SUPE–py–Imine–Cage** , (b) **SUPE–py–Amine–Cage** and (c) ACs. Note: 30 mg of I_2 and 60 mg of KI in 10 mL of H_2O for the presence of 1–100 equivalents of competing anions and 15 mg of I_2 + 30 mg of KI in 10 mL of H_2O for the presence of 10000 equivalents of competing anions.

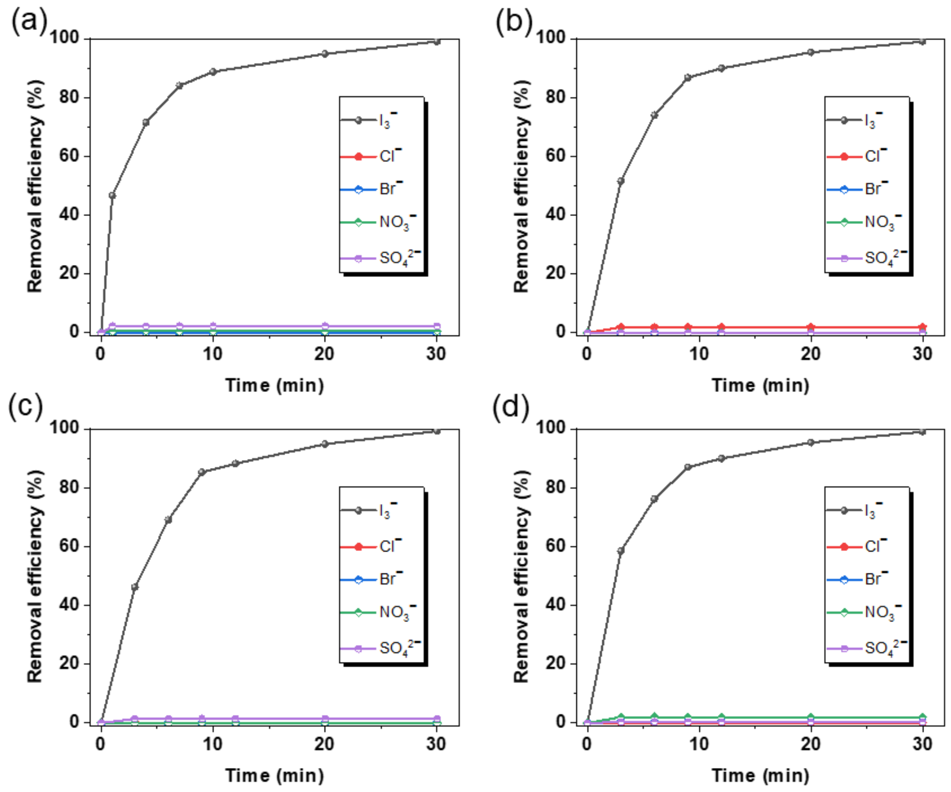

Supplementary Fig. 58 | Iodine adsorption from aqueous I_3^- solutions containing 1 equivalent of equal–molar competing anions, viz. Cl^-, Br^-, NO_3^-, and SO_4^{2-}, with **SUPE–py–Imine–Cage** at different pH: (a) pH=3, (b) pH=5, (c) pH=7 and (d) pH=10.

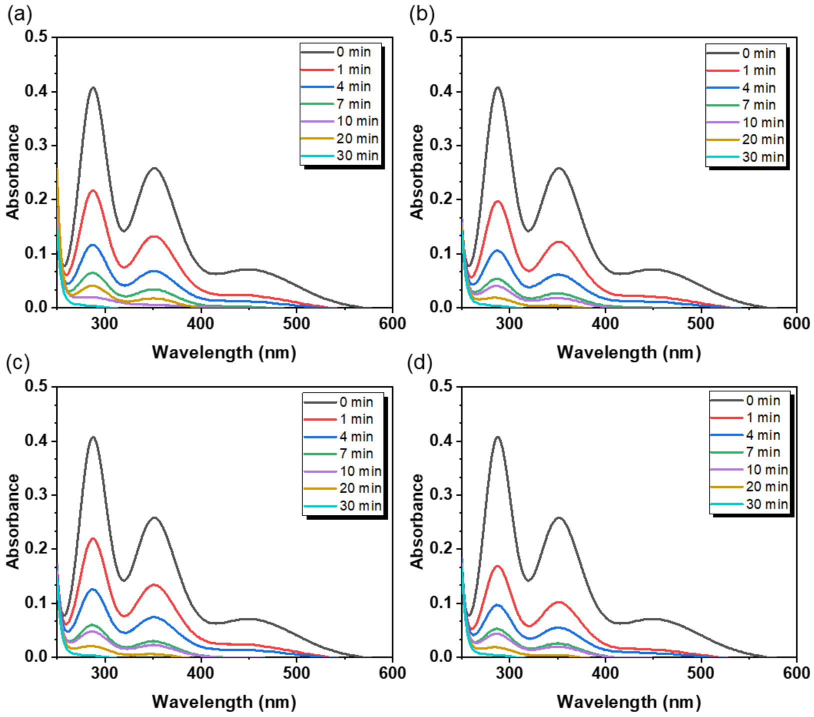

Supplementary Fig. 59 | UV/vis spectra of iodine adsorption from aqueous I_3^- solutions containing 1 equivalent of equal–molar competing anions, viz. Cl^-, Br^-, NO_3^-, and SO_4^{2-}, with **SUPE–py–Imine–Cage** at different pH: (a) pH=3, (b) pH=5, (c) pH=7 and (d) pH=10.

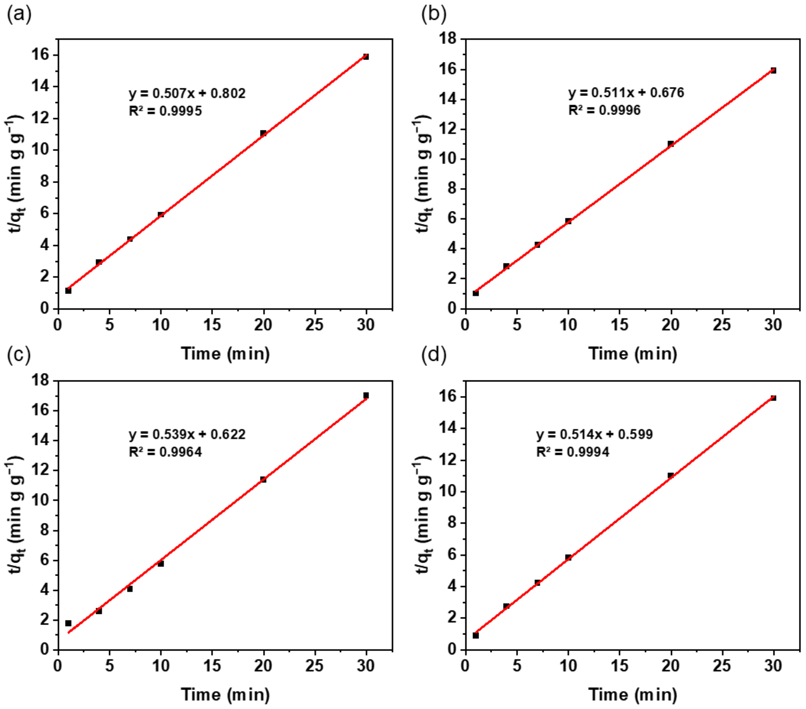

Supplementary Fig. 60 | Pseudo–second order kinetic model of iodine adsorption from different pH of aqueous I_3^- solutions containing 1 equivalent of equal–molar competing anions, viz. Cl^-, Br^-, NO_3^-, and SO_4^{2-}, for **SUPE–py–Imine–Cage**, (a) pH=3, (b) pH=5, (c) pH=7 and (d) pH=10. These data were derived from Supplementary Fig. 59.

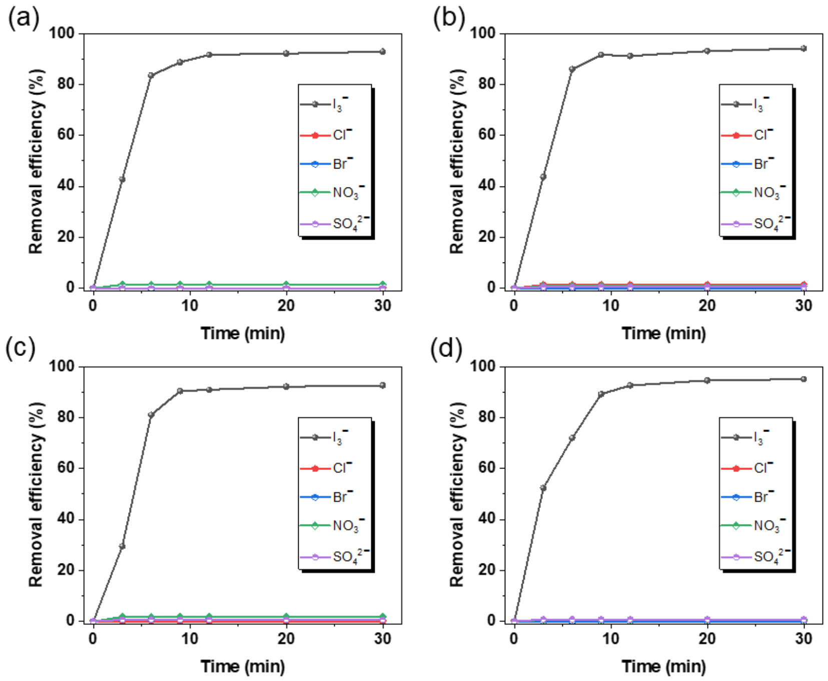

Supplementary Fig. 61 | Iodine adsorption from different pH of aqueous I_3^- solutions containing 1 equivalent of equal–molar competing anions, viz. Cl^-, Br^-, NO_3^-, and SO_4^{2-}, for **SUPE–py–Amine–Cage**, (a) pH=3, (b) pH=5, (c) pH=7 and (d) pH=10.

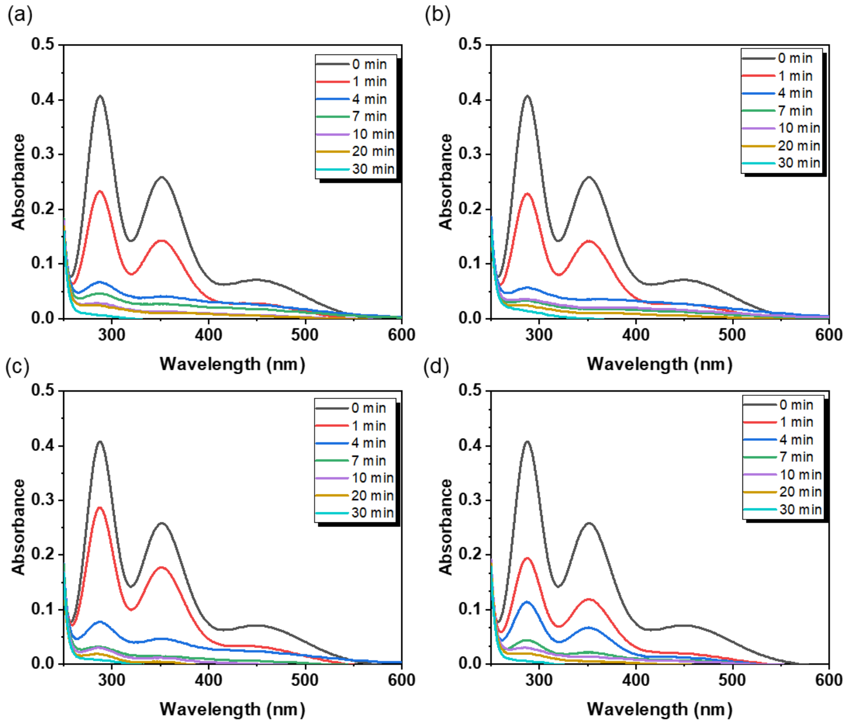

Supplementary Fig. 62 | UV/vis spectra of iodine adsorption from different pH of aqueous I_3^- solutions containing 1 equivalent of equal–molar competing anions, viz. Cl^-, Br^-, NO_3^-, and SO_4^{2-}, for **SUPE–py–Amine–Cage**, (a) pH=3, (b) pH=5, (c) pH=7 and (d) pH=10.

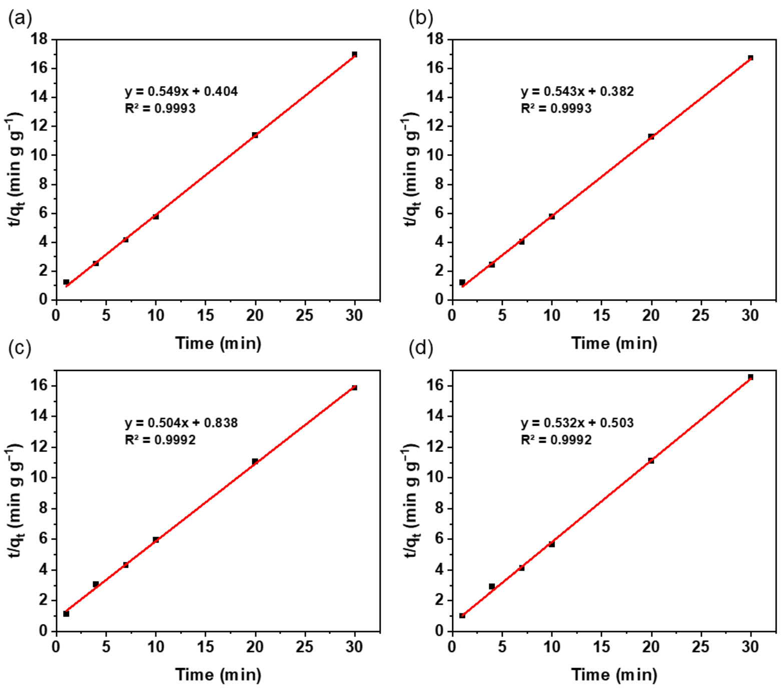

Supplementary Fig. 63 | Pseudo–second order kinetic model of iodine adsorption from different pH of aqueous I_3^- solutions containing 1 equivalent of equal–molar competing anions, viz. Cl^-, Br^-, NO_3^-, and SO_4^{2-}, for **SUPE–py–Imine–Cage**, (a) pH=3, (b) pH=5, (c) pH=7 and (d) pH=10. These data were derived from Supplementary Fig. 62.

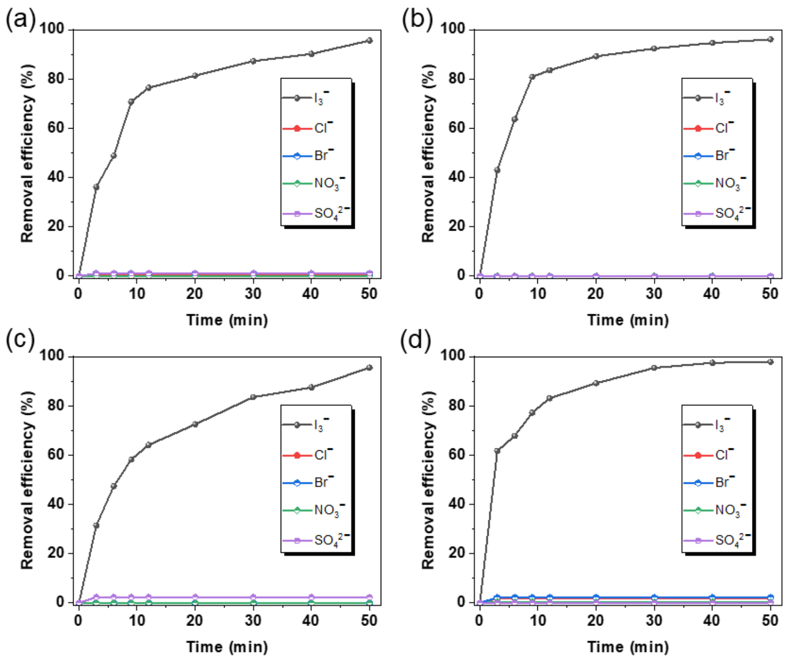

Supplementary Fig. 64 | Iodine adsorption from aqueous I_3^- solutions containing an excess (1–1000 equivalents) of equal–molar competing anions, viz. Cl^-, Br^-, NO_3^-, and SO_4^{2-}, for **SUPE–py–Imine–Cage**: (a) 1 equivalent, (b) 10 equivalents, (c) 100 equivalents, and (d) 1000 equivalents. Note: 30 mg of I_2 and 60 mg of KI in 10 mL of H_2O for the presence of 1–100 equivalents of competing anions and 15 mg of I_2 + 30 mg of KI in 10 mL of H_2O for the presence of 1000 equivalents of competing anions.

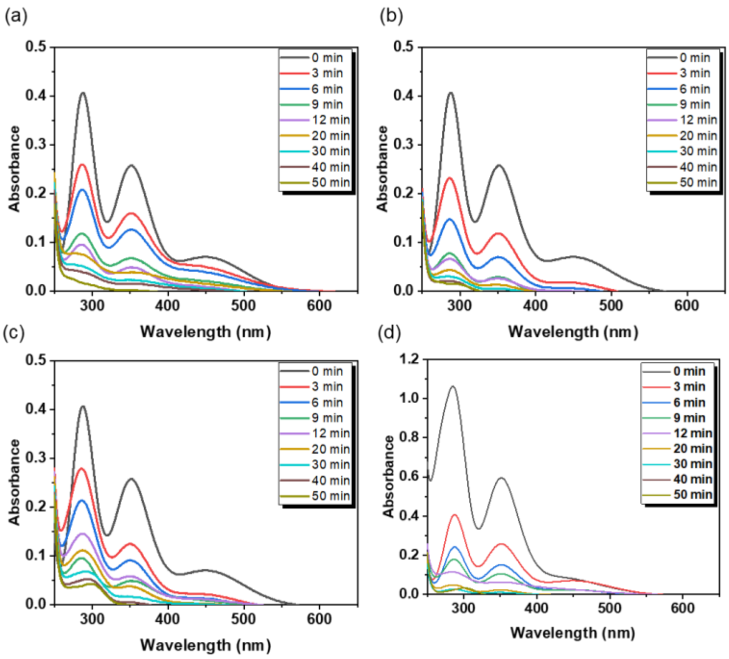

Supplementary Fig. 65 | UV/vis spectra of iodine adsorption from aqueous I_3^- solutions containing an excess (1–1000 equivalents) of equal–molar competing anions, viz. Cl^-, Br^-, NO_3^-, and SO_4^{2-}, for **SUPE–py–Imine–Cage**: (a) 1 equivalent, (b) 10 equivalents, (c) 100 equivalents, and (d) 1000 equivalents. Note: 30 mg of I_2 and 60 mg of KI in 10 mL of H_2O for the presence of 1–100 equivalents of competing anions and 15 mg of I_2 + 30 mg of KI in 10 mL of H_2O for the presence of 1000 equivalents of competing anions.

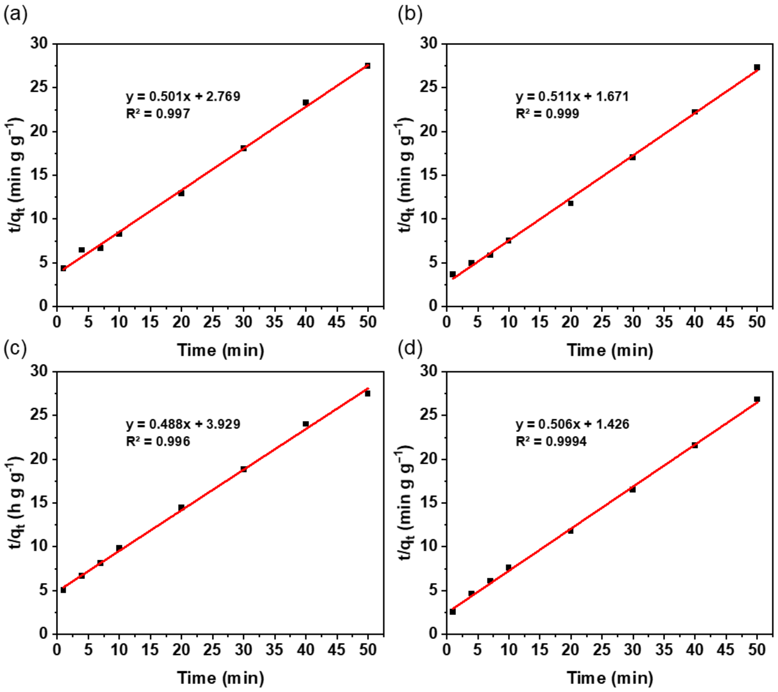

Supplementary Fig. 66 | Pseudo–second order kinetic model of iodine adsorption from aqueous I_3^- solutions containing an excess (1–1000 equivalents) of equal–molar competing anions, viz. Cl^-, Br^-, NO_3^-, and SO_4^{2-}, for **SUPE–py–Imine–Cage**: (a) 1 equivalent, (b) 10 equivalents, (c) 100 equivalents, and (d) 1000 equivalents. These data were derived from Supplementary Fig. 65. Note: 30 mg of I_2 and 60 mg of KI in 10 mL of H_2O for the presence of 1–100 equivalents of competing anions and 15 mg of I_2 + 30 mg of KI in 10 mL of H_2O for the presence of 1000 equivalents of competing anions.

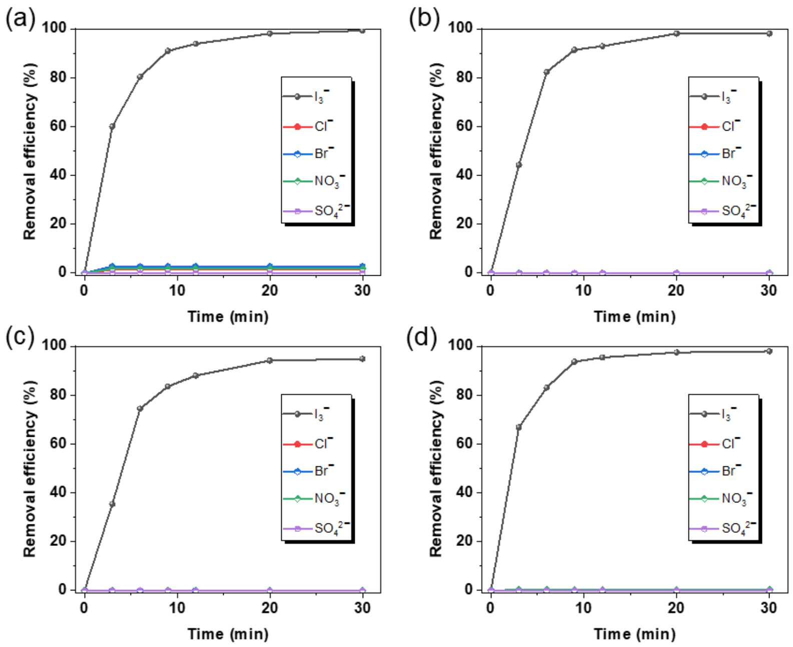

Supplementary Fig. 67 | Iodine adsorption from aqueous I_3^- solutions containing an excess (1–1000 equivalents) of equal–molar competing anions, viz. Cl^-, Br^-, NO_3^-, and SO_4^{2-}, for **SUPE–py–Amine–Cage**: (a) 1 equivalent, (b) 10 equivalents, (c) 100 equivalents, and (d) 1000 equivalents. Note: 30 mg of I_2 and 60 mg of KI in 10 mL of H_2O for the presence of 1–100 equivalents of competing anions and 15 mg of I_2 + 30 mg of KI in 10 mL of H_2O for the presence of 1000 equivalents of competing anions.

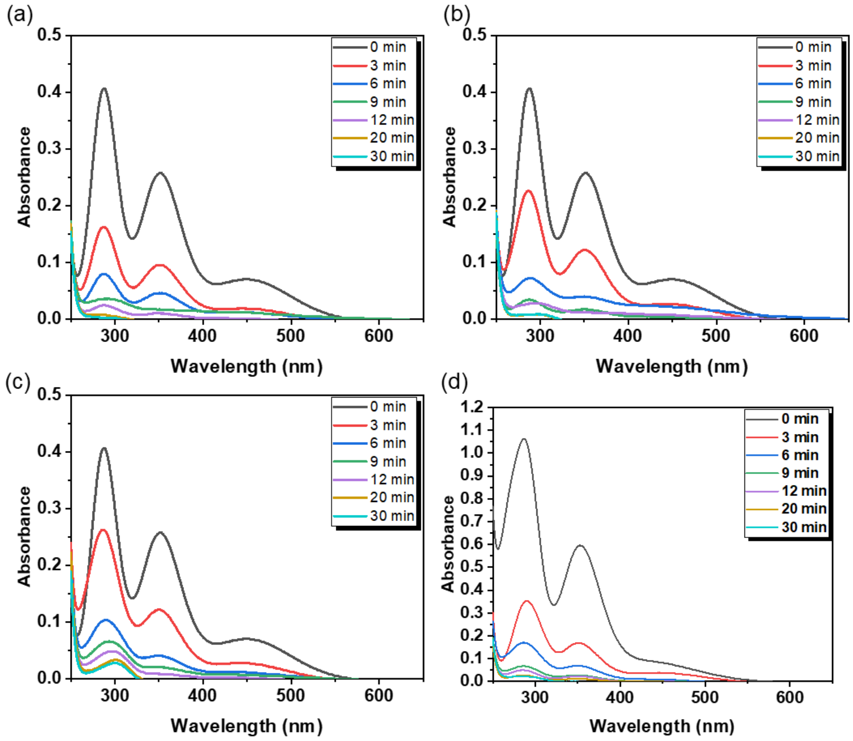

Supplementary Fig. 68 | UV/vis spectra of iodine adsorption from aqueous I_3^- solutions containing an excess (1–1000 equivalents) of equal–molar competing anions, viz. Cl^-, Br^-, NO_3^-, and SO_4^{2-}, for **SUPE–py–Amine–Cage**: (a) 1 equivalent, (b) 10 equivalents, (c) 100 equivalents, and (d) 1000 equivalents.

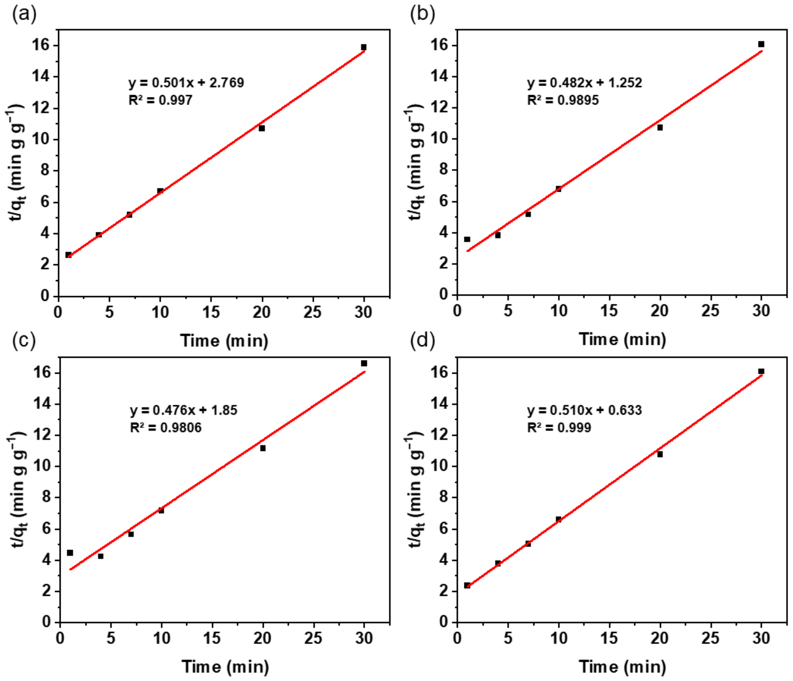

Supplementary Fig. 69 | Pseudo–second order kinetic model of iodine adsorption from aqueous I_3^- solutions containing an excess (1–1000 equivalents) of equal–molar competing anions, viz. Cl^-, Br^-, NO_3^-, and SO_4^{2-}, for **SUPE–py–Amine–Cage**: (a) 1 equivalent, (b) 10 equivalents, (c) 100 equivalents, and (d) 1000 equivalents. These data were derived from Supplementary Fig. 68.

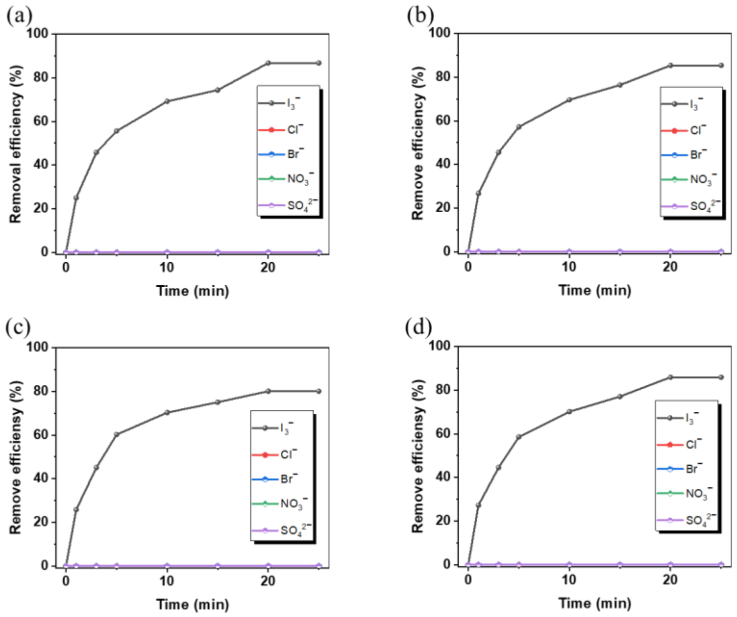

Supplementary Fig. 70 | Iodine adsorption from different pH of aqueous I_3^- solutions containing 1 equivalent of equal–molar competing anions, viz. Cl^-, Br^-, NO_3^-, and SO_4^{2-}, for ACs, (a) pH=3, (b) pH=5, (c) pH=7 and (d) pH=10.

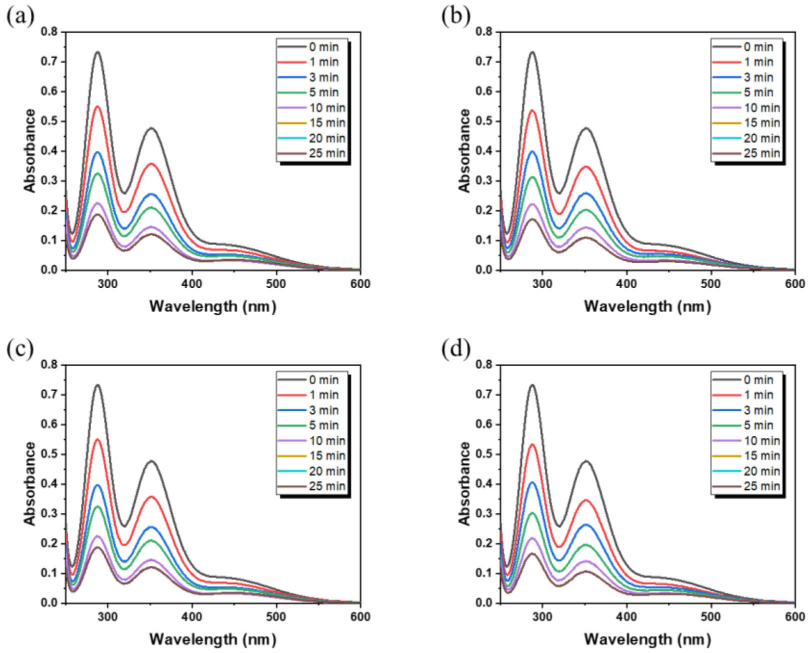

Supplementary Fig. 71 | UV/vis spectra of iodine adsorption from different pH of aqueous I_3^- solutions containing 1 equivalent of equal–molar competing anions, viz. Cl^-, Br^-, NO_3^-, and SO_4^{2-}, for ACs, (a) pH=3, (b) pH=5, (c) pH=7 and (d) pH=10.

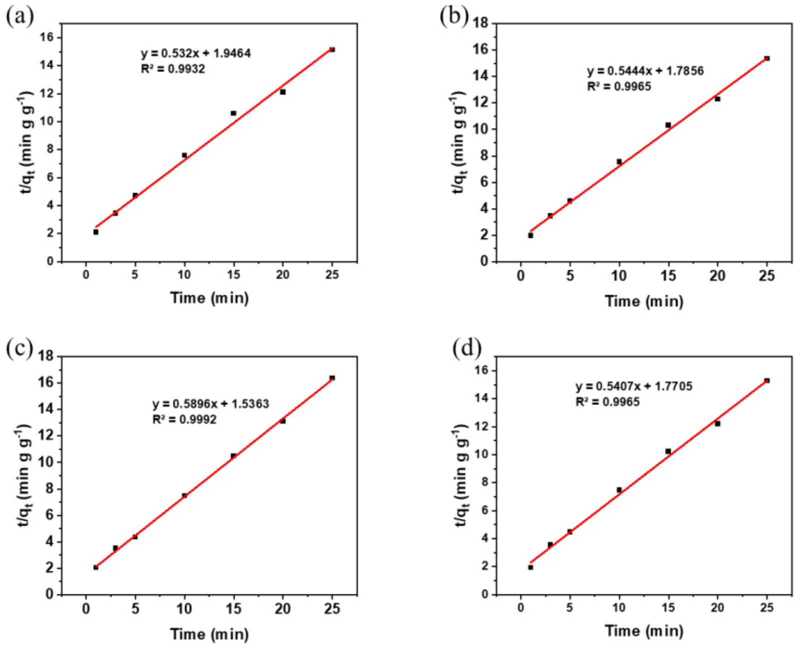

Supplementary Fig. 72 | Pseudo–second order kinetic model of iodine adsorption from different pH of aqueous I_3^- solutions containing 1 equivalent of equal–molar competing anions, viz. Cl^-, Br^-, NO_3^-, and SO_4^{2-}, for ACs, (a) pH=3, (b) pH=5, (c) pH=7 and (d) pH=10. These data were derived from Supplementary Fig. 71.

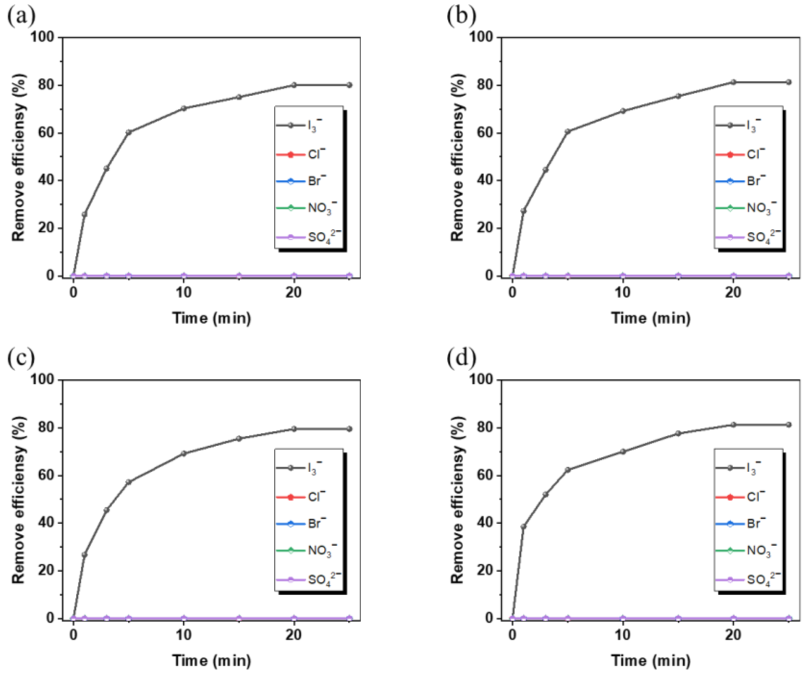

Supplementary Fig. 73 | Iodine adsorption from aqueous I_3^- solutions containing an excess (1–1000 equivalents) of equal–molar competing anions, viz. Cl^-, Br^-, NO_3^-, and SO_4^{2-}, for ACs: (a) 1 equivalent, (b) 10 equivalents, (c) 100 equivalents, and (d) 1000 equivalents. Note: 30 mg of I_2 and 60 mg of KI in 10 mL of H_2O for the presence of 1–100 equivalents of competing anions and 15 mg of I_2 + 30 mg of KI in 10 mL of H_2O for the presence of 1000 equivalents of competing anions.

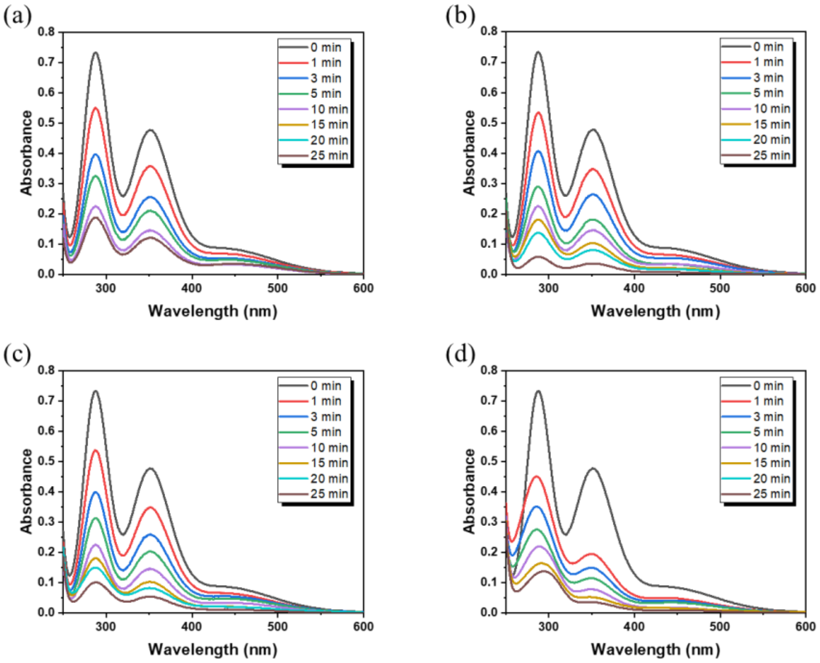

Supplementary Fig. 74 | UV/vis spectra of iodine adsorption from aqueous I_3^- solutions containing an excess (1–1000 equivalents) of equal–molar competing anions, viz. Cl^-, Br^-, NO_3^-, and SO_4^{2-}, for ACs: (a) 1 equivalent, (b) 10 equivalents, (c) 100 equivalents, and (d) 1000 equivalents.

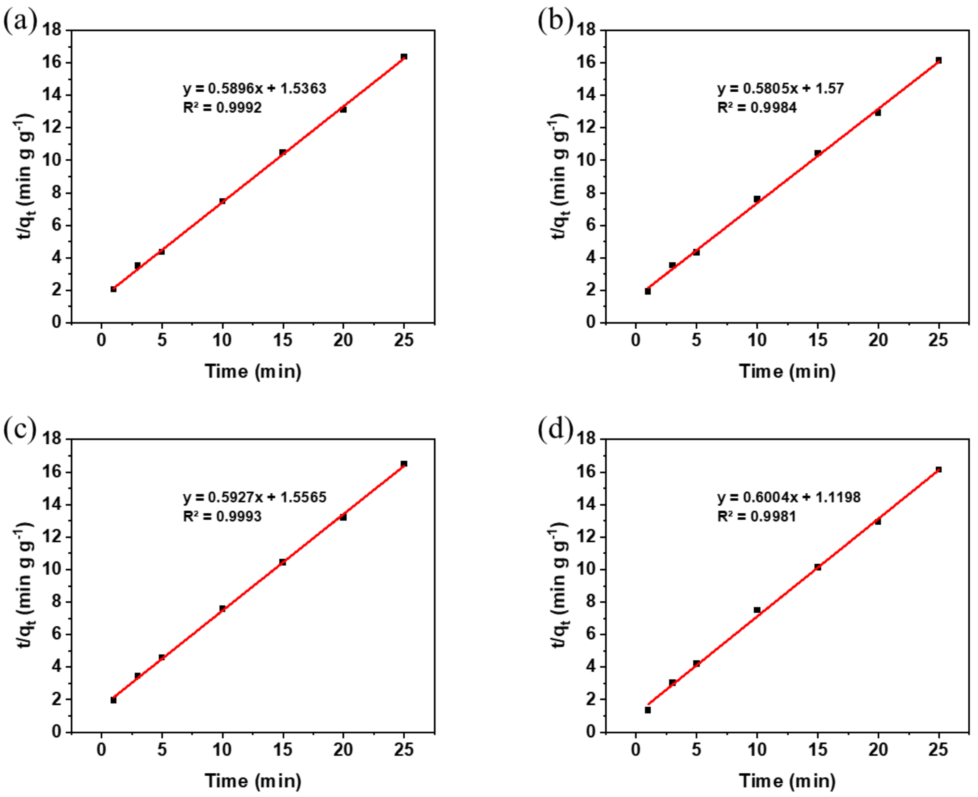

Supplementary Fig. 75 | Pseudo–second order kinetic model of iodine adsorption from aqueous I_3^- solutions containing an excess (1–1000 equivalents) of equal–molar competing anions, viz. Cl^-, Br^-, NO_3^-, and SO_4^{2-}, for ACs: (a) 1 equivalent, (b) 10 equivalents, (c) 100 equivalents, and (d) 1000 equivalents. These data were derived from Supplementary Fig. 74.

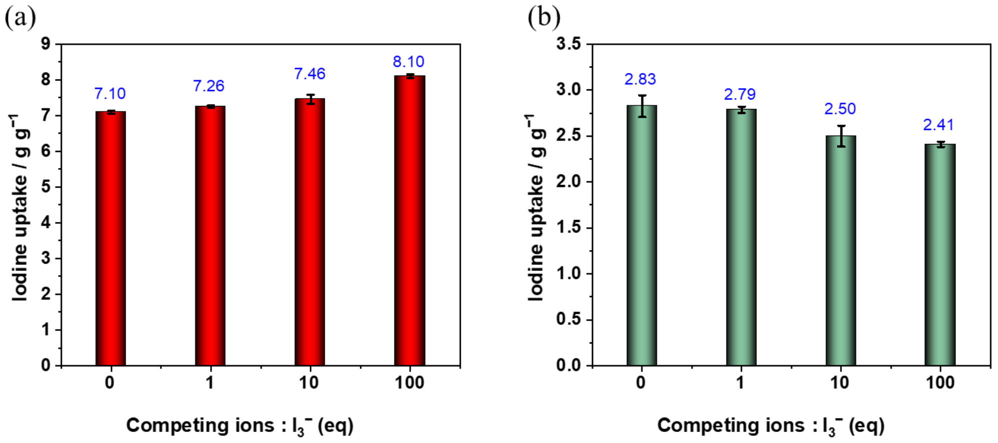

Supplementary Fig. 76 | Amount of iodine (I_3^-) adsorbed at equilibrium for (a) **SUPE–py–Imine–Cage** and (b) ACs in the presence of 1–100 equivalents of equal–molar Cl^-, Br^-, NO_3^-, and SO_4^{2-}. Error bars represent SD. n = 3 independent experiments.

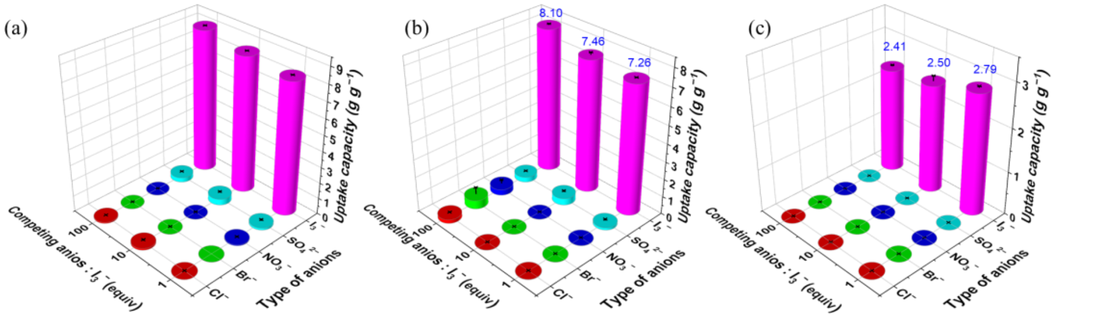

Supplementary Fig. 77 | Selective I_3^- adsorption capability from aqueous solutions containing an excess (1–100 equivalents) of equal–molar competing anions, viz. Cl^-, Br^-, NO_3^-, and SO_4^{2-}, with (a) **SUPE–py–Imine–Cage**, (b) **SUPE–py–Amine–Cage** and (c) ACs. Error bars represent SD. n = 3 independent experiments.

Supplementary Table 3 | The maximum uptake capacity of anions from I_3^-–containing aqueous solutions in the presence of various equivalents of equal–molar competing anions.

Ratio of competing anion and I_3^-	Uptake amount / g g^{-1}	I_3^-	Cl^-	Br^-	NO_3^-	SO_4^{2-}
1	7	8.25±0.04	0.002±0.002	< 0.001	0.06±0.02	0.13±0.08
	8	7.26 ±0.03	< 0.001	< 0.001	< 0.001	0.09±0.078
10	7	8.55±0.02	0.11±0.06	< 0.001	< 0.001	0.39±0.07
	8	7.46 ±0.14	< 0.001	< 0.0001	< 0.001	0.19±0.07
100	7	9.01 ±0.02	0.07±0.02	< 0.001	< 0.001	0.29±0.02
	8	8.10 ±0.05	0.02±0.02	0.49±0.40	0.33±0.22	0.09±0.02

Note: 7: SUPE–py–Imine–Cage, and 8: SUPE–py–Amine–Cage.

Supplementary Table 4 | Comparison of I_3^- maximum uptake capacity from liquid phases.

Adsorbents	Category	Hours (h)	Capacity (g g^{-1})	Ref.
SUPE–py–Imine–Cage	NAS	48	9.01	This work
SUPE–py–Amine–Cage	NAS	48	8.10	This work
COFA–1	COF	12	7.13	4
PP–3	POP	24	5.9	9
PP–2	POP	24	5.8	9

PP–4	POP	24	5.1	9
COFP–1	COF	12	4.81	4
PP–1	POP	24	4.2	9
PP–5	POP	24	4.0	9
HCOFs–4	COF	24	3.73	10
MOF nanosheets	MOF	72	3.704	11
N–MOF–PAN fibers	MOF		3.56	12
compound–1	POP	24	3.5	13
HCOFs–2	COF	24	3.42	10
compound–2	POP	24	3.4	13
C[4]P–BT	POP	48	3.24	5
C–poly–I_5	POP	24	3.2	14
HCOFs–3	COF	24	3.15	10
CaCOP3	COP	4	3.1	15
C[4]P–TPE	POP	48	2.99	5
HCP–91	POP	24	2.9	16
ACs	ACM	48	2.83	This work
CaCOP2	COP	6	2.81	15
C[4]P–TTP	POP	48	2.51	5
HCP–92	POP	24	2.49	16
C[4]P–BP	POP	48	2.37	5
C[4]P–BTP	POP	48	2.32	5
CalCOP1	COP	72	2.32	17

MeO–CTF600	COF	40	2.21	18
HCOFs–1	COF	24	2.1	19
C[4]P–DPP	POP	48	1.58	5
3D bulk MOF	MOF	72	1.333	11
BC@Dopa–ZIF	MOF	50	1.31	20
LFC–KN	ACM		1.295	21
SVAC	ACM	1.5	1.178	22
$[Mn_2(oxdz)_2(tpbn)(H_2O)_2]\cdot 2C_2H_5OH\}_n$	MOF	36	1.1	23

Note: ACM: active carbon materials, COF: covalent – organic framework, POP: porous organic polymers, MOF: metal organic framework and NAS: nonporous amorphous (superad)sorbents

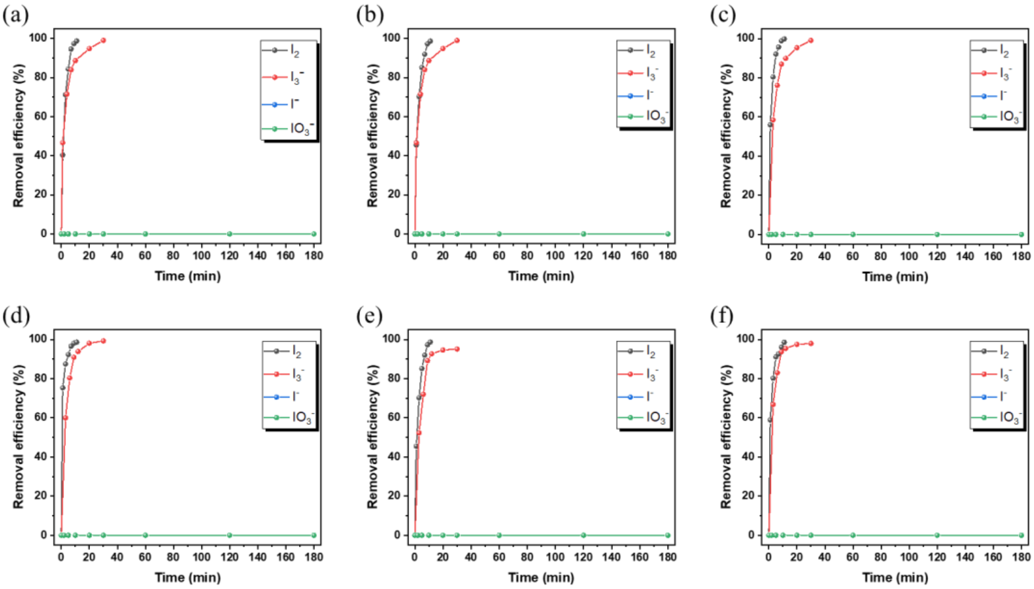

Supplementary Fig. 78 | The removal efficiency of I_2, I_3^-, I^- and IO_3^- in aqueous solutions at (a) pH=3, (b) pH=7, (c) pH=10 with **SUPE–py–Imine–Cage** and in aqueous solutions at (d) pH=3, (e) pH=7 and (f) pH=10 with **SUPE–py–Amine–Cage**.

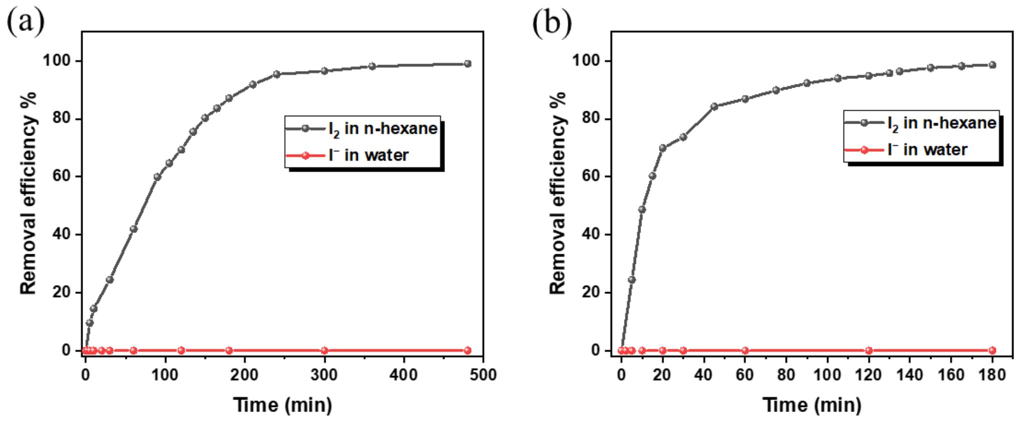

Supplementary Fig. 79 | I_2 and I^- removal efficiency in n–hexane and water, respectively. (a) **SUPE–py–Imine–Cage** and (b) **SUPE–py–Amine–Cage**.

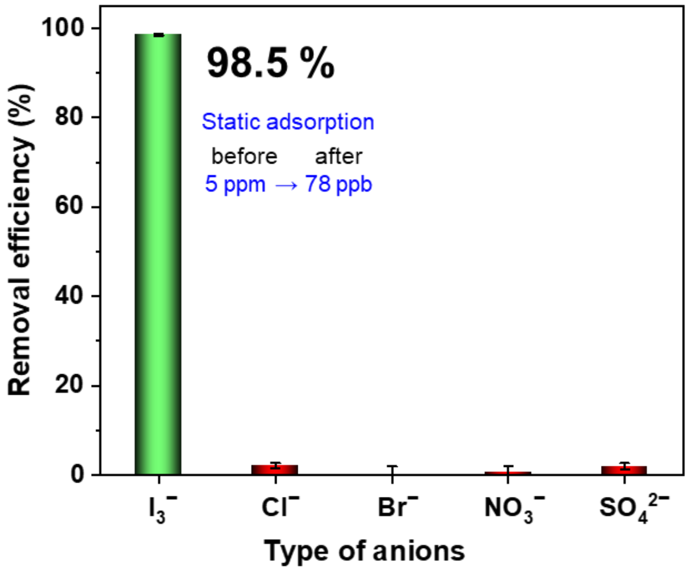

Supplementary Fig. 80 | Static adsorption of I_3^- from contaminated water with trace I_3^- (5.0 ppm) in the presence of 1 to 1000–fold excess of co–existing competing anions (equal–molar Cl^-, Br^-, NO_3^-, and SO_4^{2-}) with **SUPE–py–Amine–Cage**. Error bars represent SD. n = 3 independent experiments.

Supplementary Note 4. Dynamic flow–through adsorption from aqueous iodine solutions

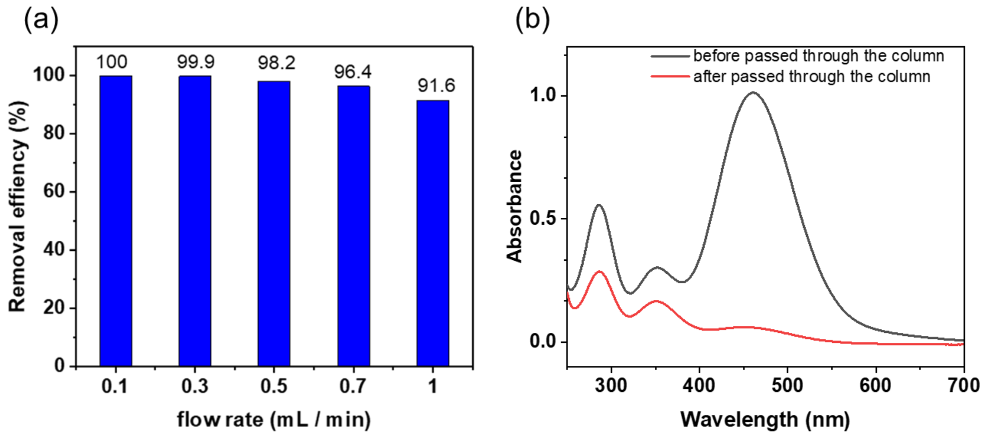

Supplementary Fig. 81 | (a)The removal efficiency of iodine from water via dynamic flow–through iodine adsorption with different flow rates and (b) UV–vis spectra of the I_2–bearing aqueous solutions before (in black) and after (in red) the dynamic flow–through iodine adsorption with **SUPE–py–Imine–Cage**.

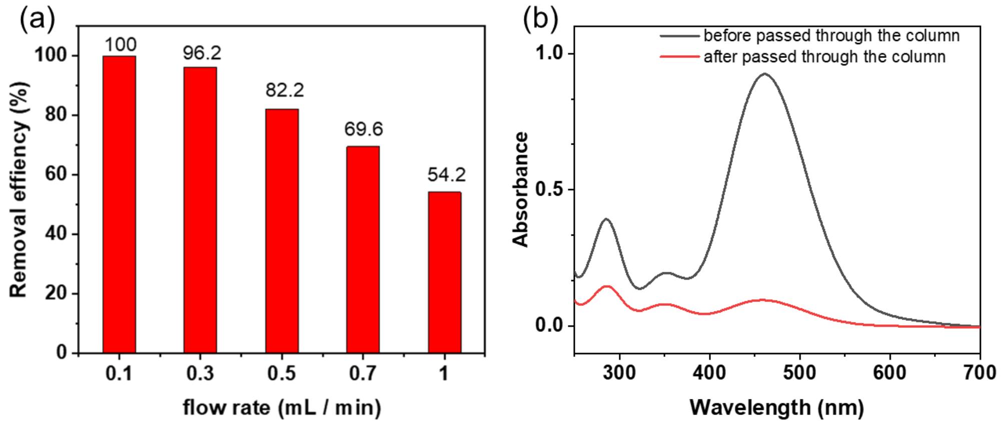

Supplementary Fig. 82 | (a)The removal efficiency of iodine from water via dynamic flow–through iodine adsorption with different flow rates and (b) UV–vis spectra of the I_2–bearing aqueous solutions before (in black) and after (in red) the dynamic flow–through iodine adsorption with **SUPE–py–Amine–Cage**.

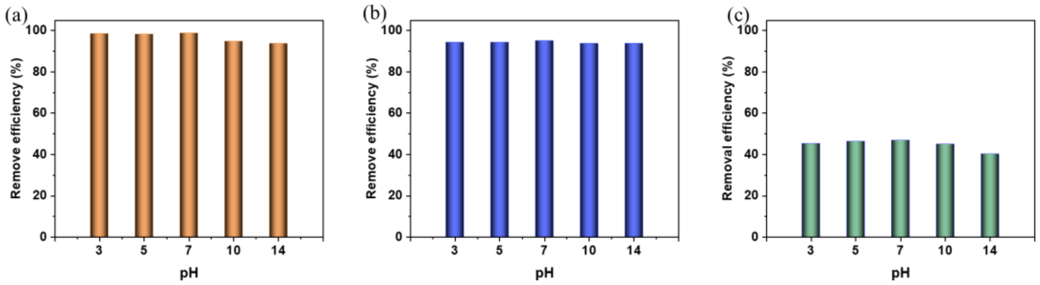

Supplementary Fig. 83 | The pH effect on dynamic flow–through adsorption of I_2 with (a) **SUPE–py–Imine–Cage** and (b) **SUPE–py–Amine–Cage** and (c) ACs.

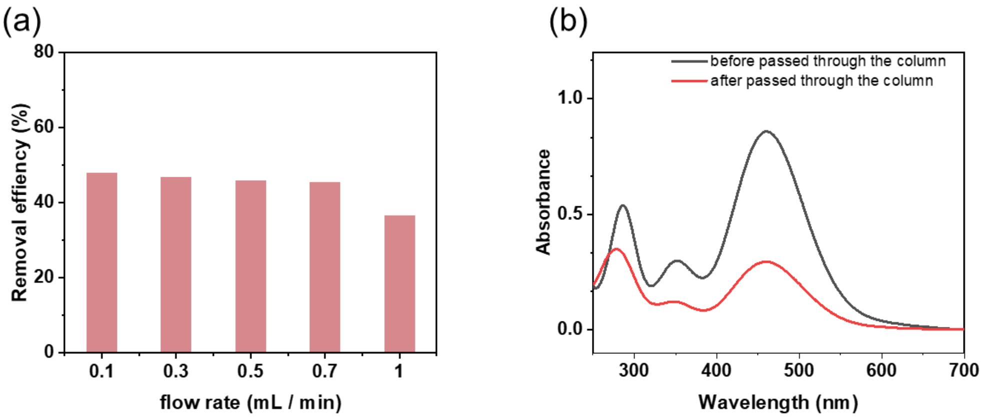

Supplementary Fig. 84 | (a)The removal efficiency of iodine from water via dynamic flow–through iodine adsorption with different flow rates and (b) UV–vis spectra of the I_2–bearing aqueous solutions before (in black) and after (in red) the dynamic flow–through iodine adsorption with ACs.

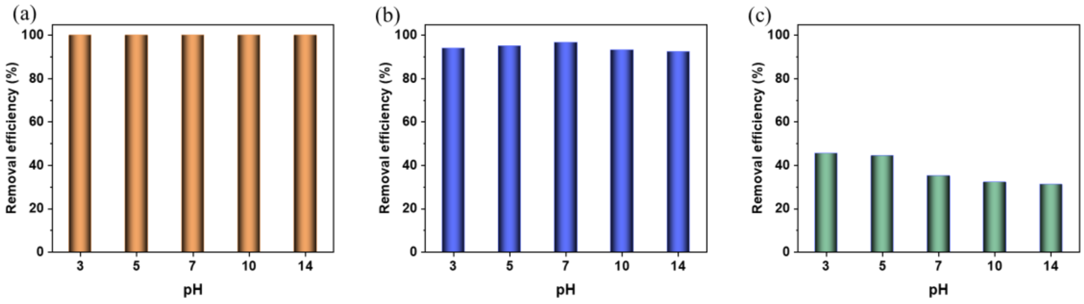

Supplementary Fig. 85 | The pH effect on dynamic flow–through adsorption of I_3^- (I_2/KI) with (a) **SUPE–py–Imine–Cage** , (b) **SUPE–py–Amine–Cage** and (c) ACs.

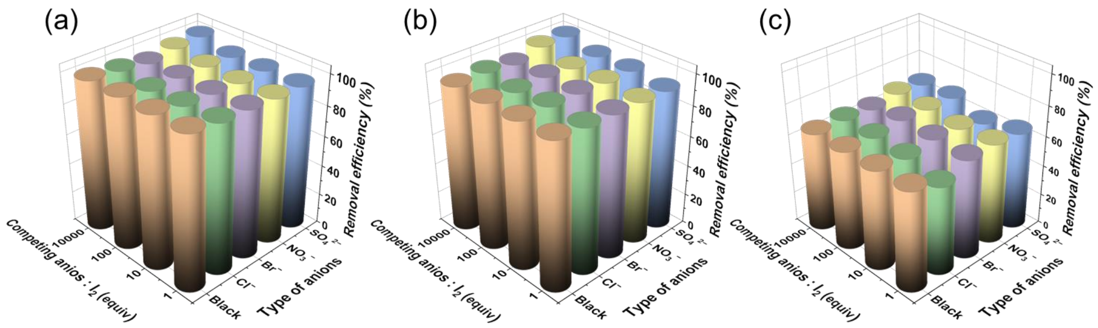

Supplementary Fig. 86 | Dynamic removal efficiency (relative uptake) of iodine from a binary aqueous mixture of I_2 and (1 to 1000 equivalents of) Cl^-, Br^-, NO_3^-, or SO_4^{2-} using (a) **SUPE–py–Imine–Cage**, (b) **SUPE–py–Amine–Cage** and (c) ACs. Note: the I_2 concentration is 1.0 mM for the presence of 1–100 equivalents of competing anions and 0.5 mM for the presence of 10000 equivalents of competing anions.

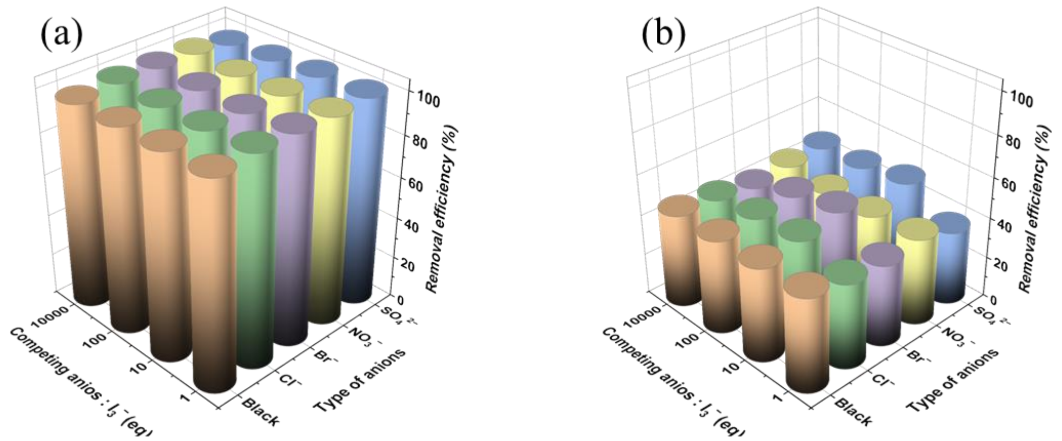

Supplementary Fig. 87 | Dynamic removal efficiency (relative uptake) of iodine from a binary aqueous mixture of I_3^- and (1 to 10000 equivalents of) Cl^-, Br^-, NO_3^-, or SO_4^{2-} using (a) **SUPE–py–Imine–Cage** and (b) ACs. Note: 30 mg of I_2 and 60 mg of KI in 10 mL of H_2O for the presence of 1–100 equivalents of competing anions and 15 mg of I_2 + 30 mg of KI in 10 mL of H_2O for the presence of 10000 equivalents of competing anions.

Supplementary Table 5 | The maximum uptake capacity of anions from I_2–containing aqueous solutions in the presence of various equivalents of equal–molar competing anions during the dynamic flow–through experiment.

Competing anions:I_2	Uptake amount / g g^{-1}	I_2	Cl^-	Br^-	NO_3^-	SO_4^{2-}
1	7	3.55 ± 0.43	< 0.001	< 0.001	< 0.001	< 0.001
	8	2.84 ± 0.47	< 0.001	< 0.001	< 0.001	< 0.001
10	7	3.59 ± 0.07	< 0.001	< 0.001	< 0.001	< 0.001
	8	2.81 ± 0.1	< 0.001	< 0.001	< 0.001	< 0.001
100	7	3.51 ± 0.01	< 0.001	< 0.001	< 0.001	< 0.001
	8	2.91 ± 0.07	< 0.001	< 0.001	< 0.001	< 0.001

Note: 7: SUPE–py–Imine–Cage, and 8: SUPE–py–Amine–Cage.

Supplementary Table 6 | The maximum uptake capacity of anions from I_3^-–containing aqueous solutions in the presence of various equivalents of equal–molar competing anions during the dynamic flow–through experiment.

Competing anions:I_3^- (equiv.)	Uptake amount / g g^{-1}	I_3^-	Cl^-	Br^-	NO_3^-	SO_4^{2-}
1	7	4.97 ±0.66	0.01 ±0.01	0.03 ± 0.03	0.03 ± 0.03	0.04 ± 0.03
	8	3.49 ± 0.41	0.01 ± 0.06	0.01 ± 0.09	0.01 ± 0.08	0.01 ± 0.08
10	7	5.3±0.51	< 0.001	< 0.001	< 0.001	< 0.001
	8	3.61±0.09	< 0.001	< 0.001	< 0.001	< 0.001
100	7	5.79 ±0.05	0.05 ± 0.03	0.007±0.004	0.11±0.12	0.04 ± 0.03
	8	4.52 ±0.22	< 0.001	< 0.001	0.09±0.11	0.01 ± 0.08

Note: 7: SUPE–py–Imine–Cage, and 8: SUPE–py–Amine–Cage.

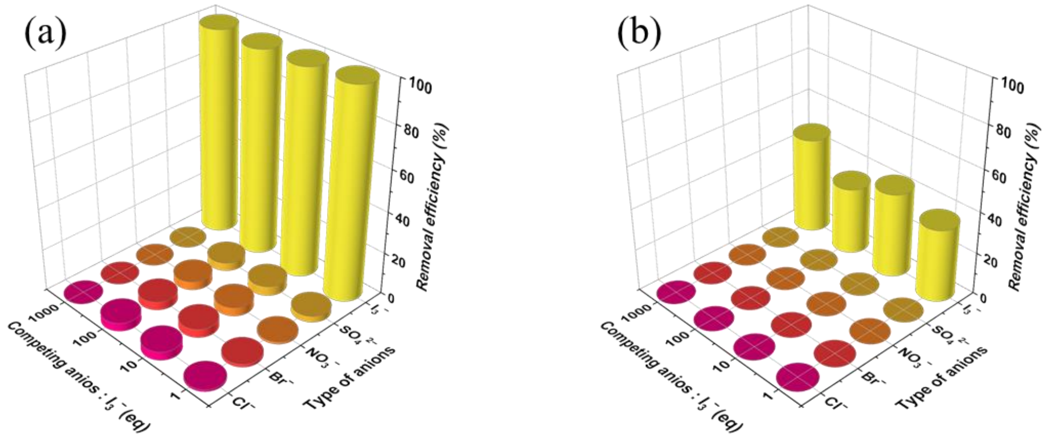

Supplementary Fig. 88 | Dynamic removal efficiency (relative uptake) of anions from aqueous I_3^- solutions containing an excess (1 to 1000 equivalents) of equal–molar competing anions, viz. Cl^-, Br^-, NO_3^-, and SO_4^{2-}, with (a) **SUPE–py–Amine–Cage** and (b) ACs. Note: 30 mg of I_2 and 60 mg of KI in 10 mL of H_2O for the presence of 1–100 equivalents of competing anions and 15 mg of I_2 + 30 mg of KI in 10 mL of H_2O for the presence of 1000 equivalents of competing anions.

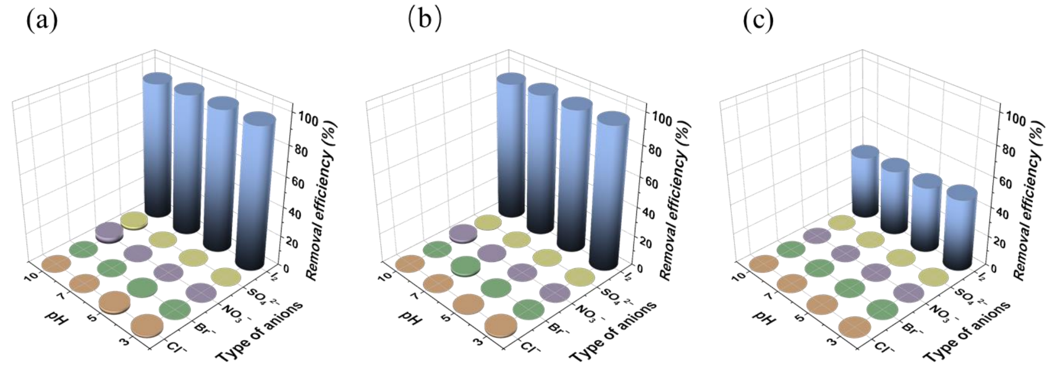

Supplementary Fig. 89 | Dynamic selective iodine adsorption from aqueous I_2–bearing solutions different pHs (3 to 10) in the presence of 1 equivalent of equal–molar competing anions, viz. Cl^-, Br^-, NO_3^-, and SO_4^{2-}, using (a) **SUPE–py–Imine–Cage** , (b) **SUPE–py–Amine–Cage** and (c) ACs.

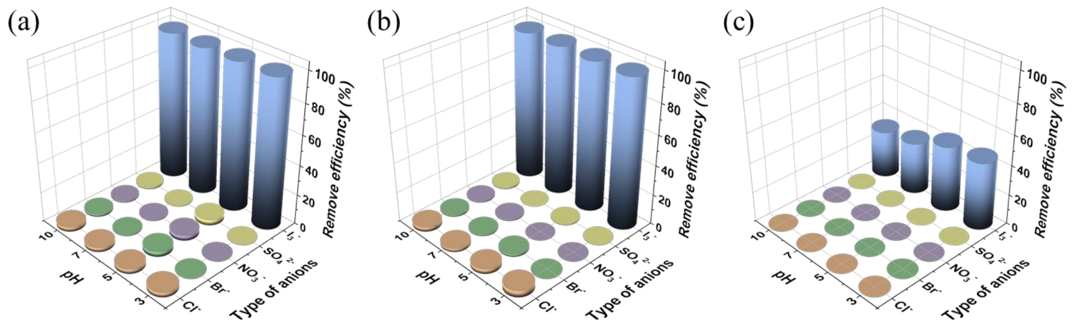

Supplementary Fig. 90 | Dynamic selective iodine adsorption from aqueous I_3^-–bearing solutions different pHs (3 to 10) in the presence of 1 equivalent of equal–molar competing anions, viz. Cl^-, Br^-, NO_3^-, and SO_4^{2-}, using (a) **SUPE–py–Imine–Cage**, (b) **SUPE–py–Amine–Cage** and (c) ACs.

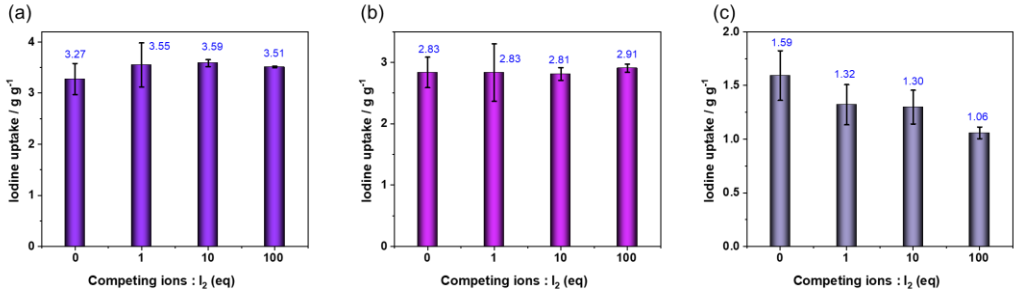

Supplementary Fig. 91 | Dynamically captured amounts of iodine adsorbed in the flow–through experiments using (a) **SUPE–py–Imine–Cage**,(b) **SUPE–py–Amine–Cage** and (c) Acs from I_2–containing aqueous mixtures with 0 to 100 equivalents of equal–molar Cl^-, Br^-, NO_3^-, and SO_4^{2-}. Error bars represent SD. n = 3 independent experiments.

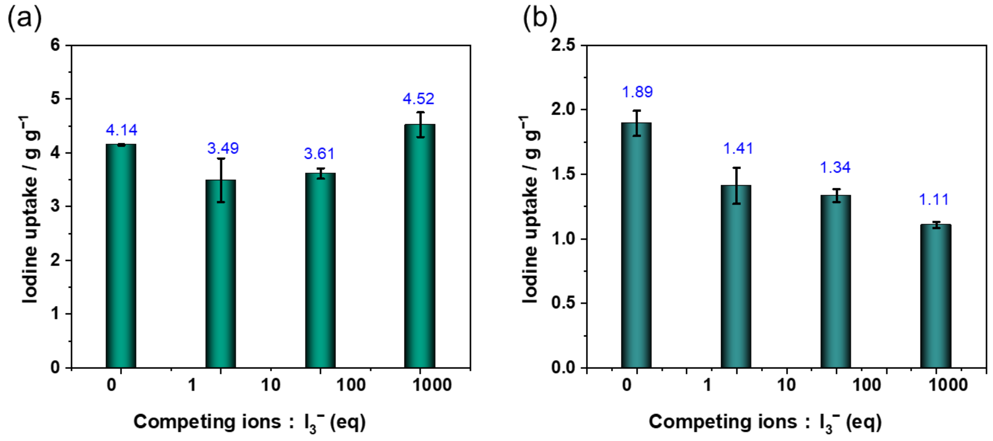

Supplementary Fig. 92 | Dynamically captured amounts of iodine in the flow–through experiments using (a) **SUPE–py–Amine–Cage** and (b) ACs from I_3^-–containing aqueous mixtures with 0 to 100 equivalents of equal–molar Cl^-, Br^-, NO_3^-, and SO_4^{2-}. Error bars represent SD. n = 3 independent experiments.

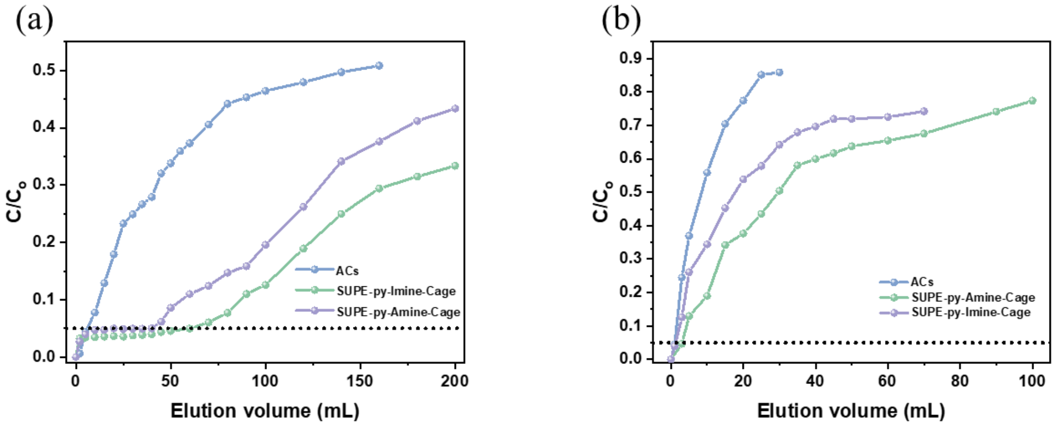

Supplementary Fig. 93 | Breakthrough curve of the ACs, **SUPE–py–Imine–Cage** and **SUPE–py–Amine–Cage** for Iodine removal. (a) saturated I_2 aqueous solution and (b) I_3^- aqueous solution (600 mg KI and 300 mg I_2 in 100 mL of H_2O).

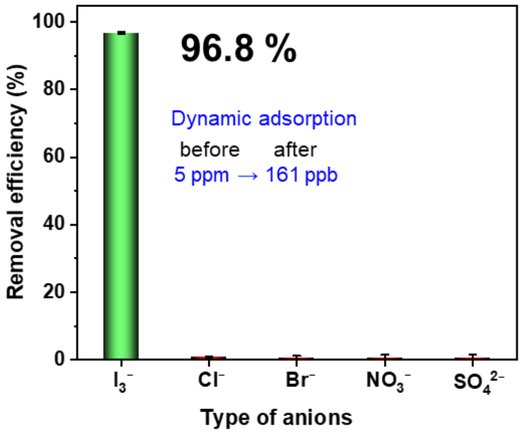

Supplementary Fig. 94 | Dynamic iodine adsorption of I_3^- from contaminated water with trace I_3^- (5.0 ppm) using **SUPE–py–Amine–Cage**. Error bars represent SD. n = 3 independent experiments.

Supplementary Note 5. Recyclability and reusability

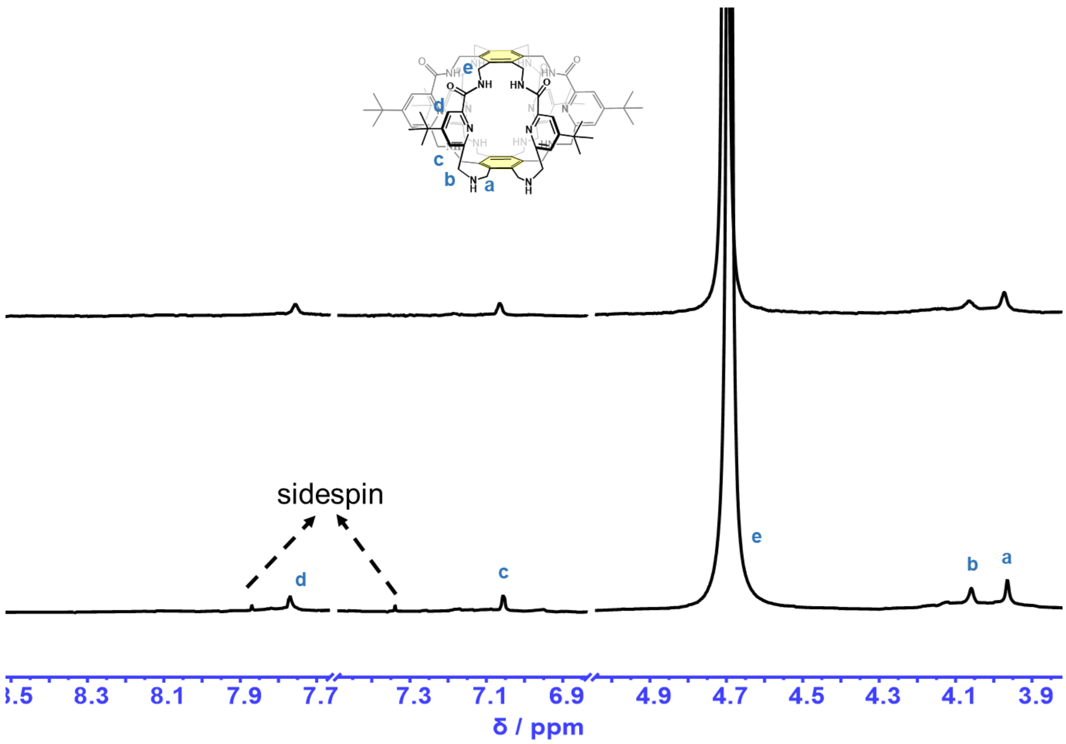

Supplementary Fig. 95 | Partial ^{1}H NMR spectra of fresh (bottom) and recycled (top) **SUPE–py–Imine–Cage** (2.0 mM) in $CDCl_3/CD_3OD$ (1/1, v/v) after flow–through iodine (I_3^-) adsorption.

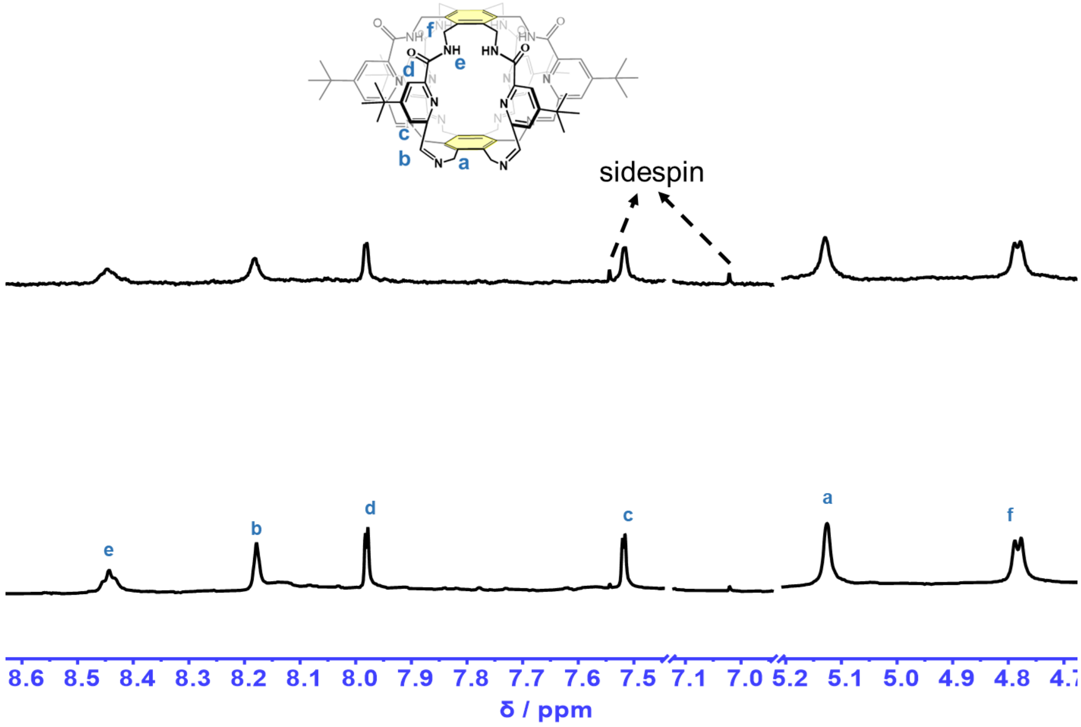

Supplementary Fig. 96 | Partial ^{1}H NMR spectra of a 2.0 mM solution of fresh **SUPE–py–Imine–Cage** (bottom) and recycled **SUPE–py–mine–Cage** from iodine vapour adsorption (top) in $CDCl_3$.

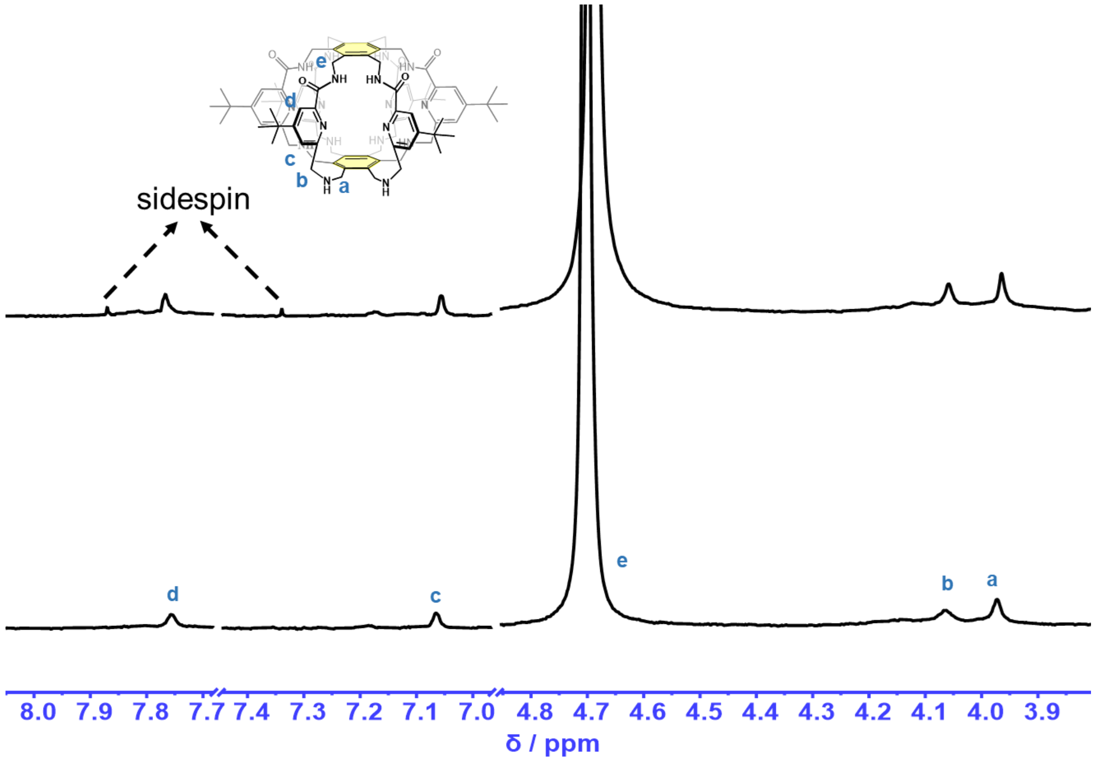

Supplementary Fig. 97 | Partial ^{1}H NMR spectra of a 2.0 mM solution of fresh **SUPE–py–Amine–Cage** (bottom) and recycled **SUPE–py–Amine–Cage** from iodine vapour adsorption (top) in $CDCl_3/CD_3OD$ (1/1, v/v).

.

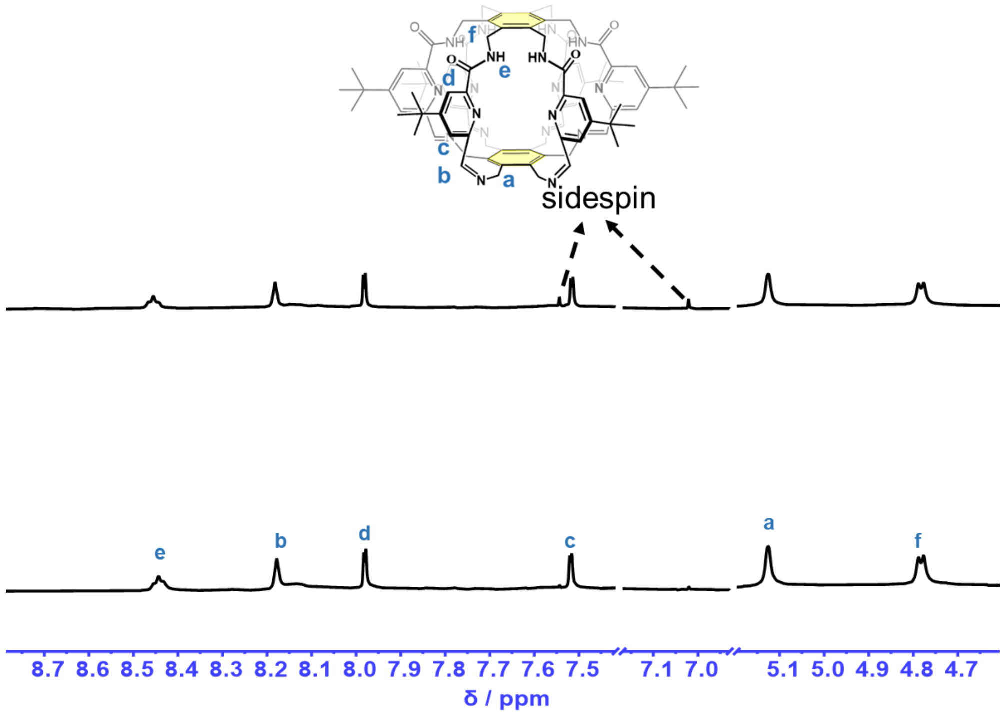

Supplementary Fig. 98 | Partial ^{1}H NMR spectra of fresh (bottom) and recycled (top) **SUPE–py–Imine–Cage** (2.0 mM) in $CDCl_3$ after static iodine (I_3^-) adsorption.

.

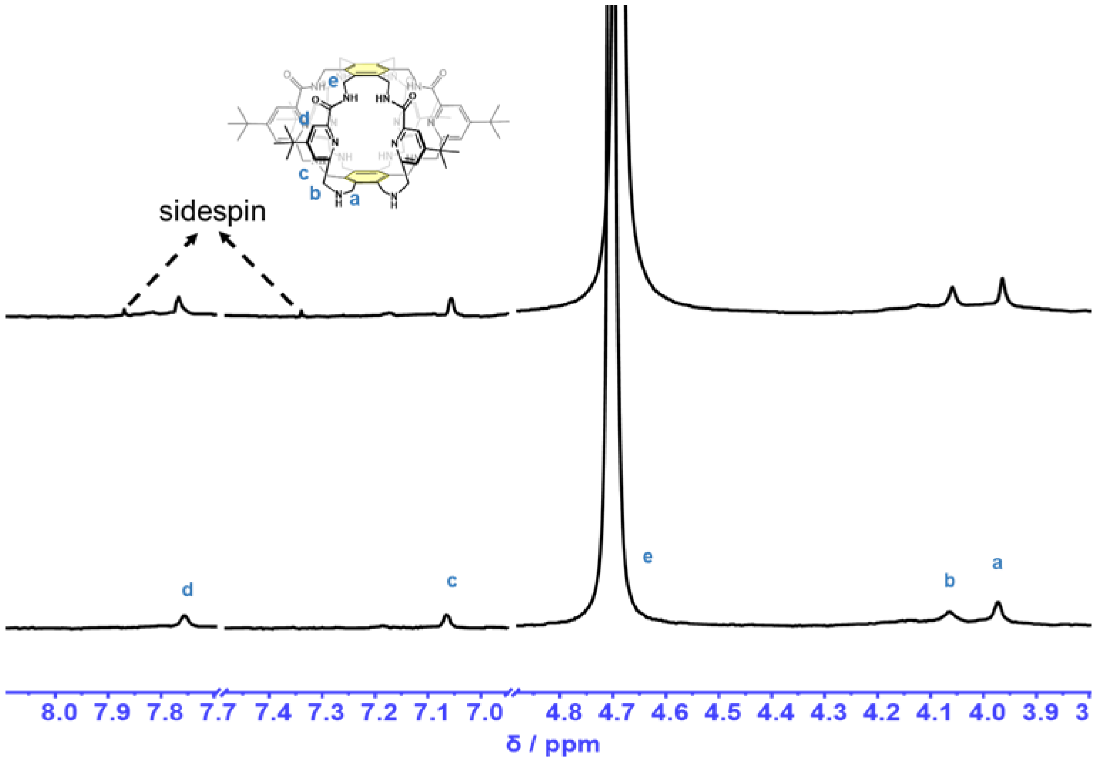

Supplementary Fig. 99 | Partial ^{1}H NMR spectra of fresh (bottom) and recycled (top) **SUPE–py–Amine–Cage** (2.0 mM) after static iodine (I_3^-) adsorption in $CDCl_3/CD_3OD$ (1/1, v/v).

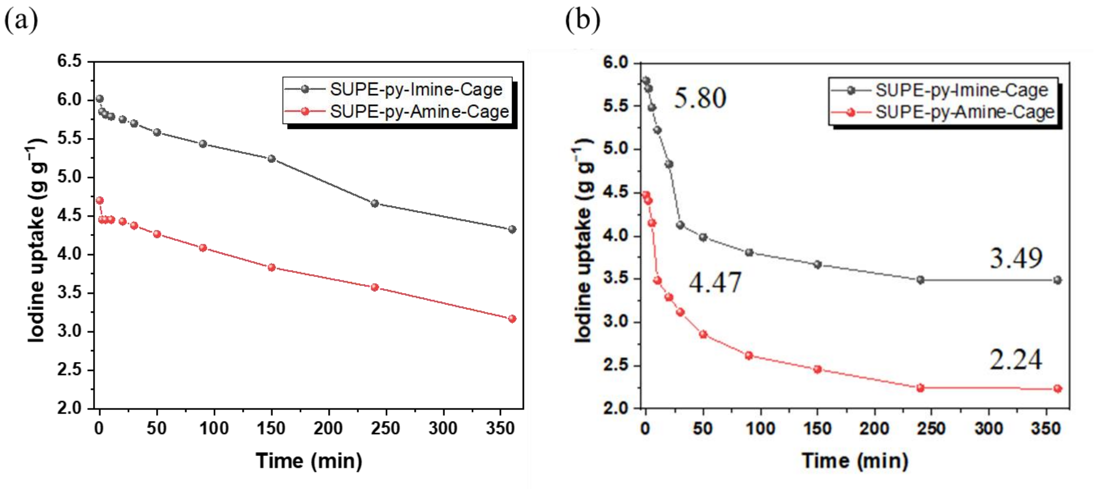

Supplementary Fig. 100 The iodine desorption profile of I_2@**SUPE–py–Imine–Cage** (in black) and I_2@**SUPE–py–Amine–Cage** (in red) (a) at 75 °C under ambient pressure and (b) at 75 °C under vacuum.

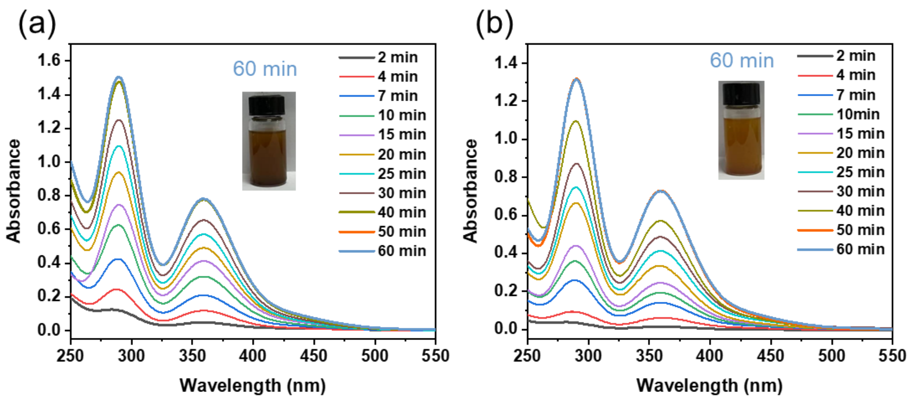

Supplementary Fig. 101 | UV/Vis spectra of I_3^- released in an isopropanol solution with sonication at different time from (a) **SUPE–py–Imine–Cage** and (b) **SUPE–py–Amine–Cage**.

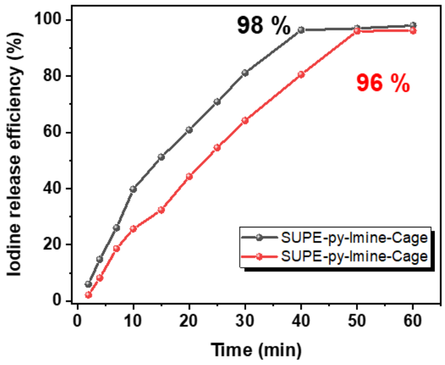

Supplementary Fig. 102 | Iodine release efficiency of I_3^- in an isopropanol solution from **SUPE–py–Imine–Cage** (black) and **SUPE–py–Amine–Cage** (red) under sonication for 60 min.

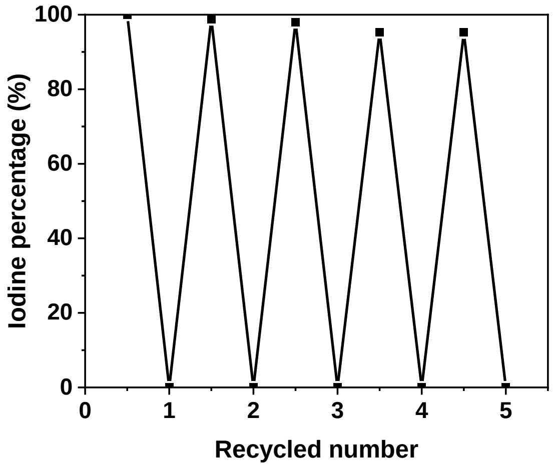

Supplementary Fig.103 | The iodine removal efficiency with recycled adsorbent **SUPE–py–Amine–Cage** during 5 cycles in the dynamic iodine (I_3^-) adsorption experiments.

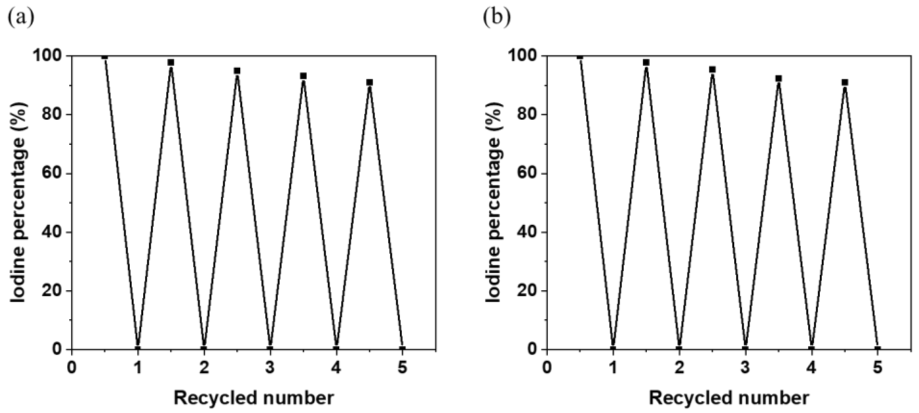

Supplementary Fig. 104 |The iodine removal efficiency of (a) **SUPE–py–Imine–Cage** and (b) **SUPE–py–Amine–Cage** during 5 cycles in the iodine vapor adsorption experiments.

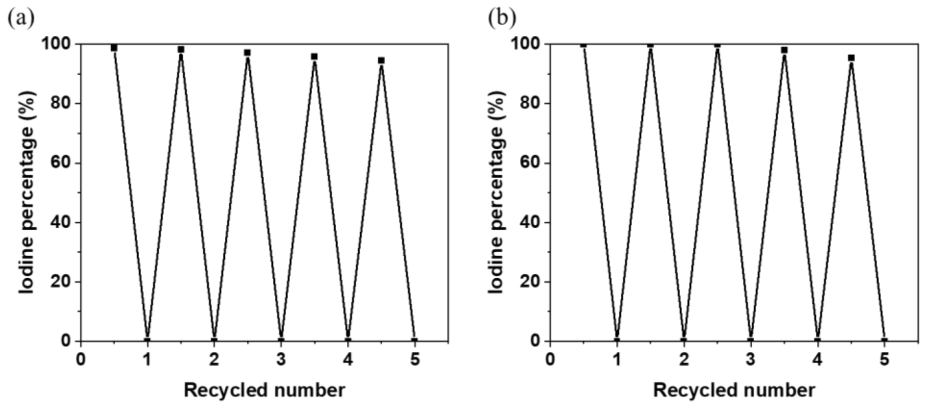

Supplementary Fig. 105 | The iodine removal efficiency of (a) **SUPE–py–Imine–Cage** and (b) **SUPE–py–Amine–Cage** during 5 cycles in the static iodine (I_3^-) adsorption experiments.

Supplementary Note 6. Mechanistic Study

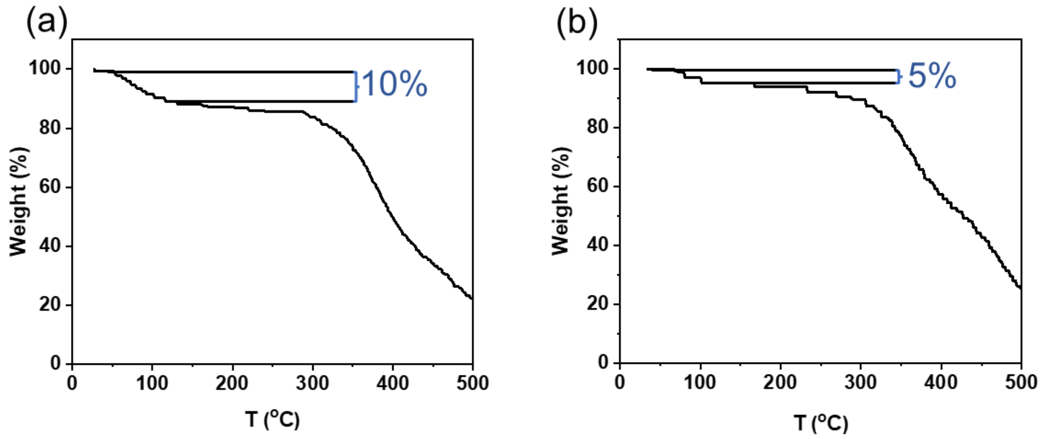

Supplementary Fig. 106 | Thermogravimetric analysis (TGA) plot from temperature–ramped measurements of (a) **SUPE–py–Imine–Cage** and (b) **SUPE–py–Amine–Cage**.

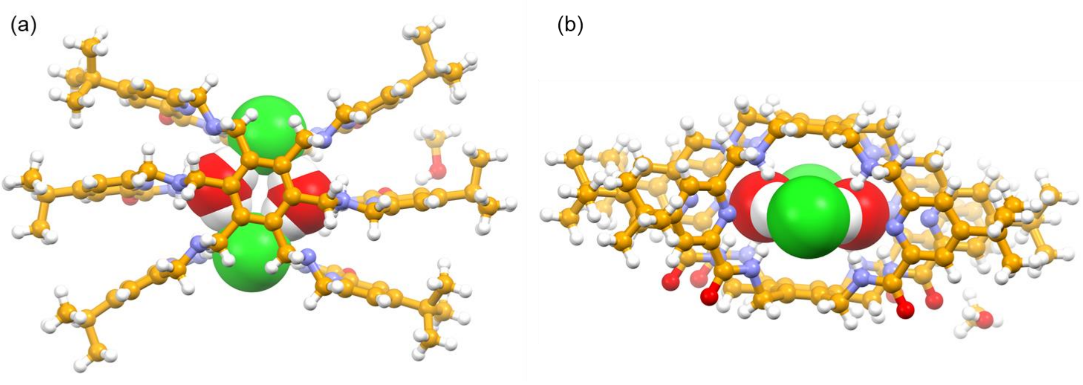

Supplementary Fig. 107 | Single–crystal structure of $2H_2O{\cdot}2Cl^{-}{\subset}\mathbf{8}{\cdot}2H^{+}$. (a) top view and (b) front view.

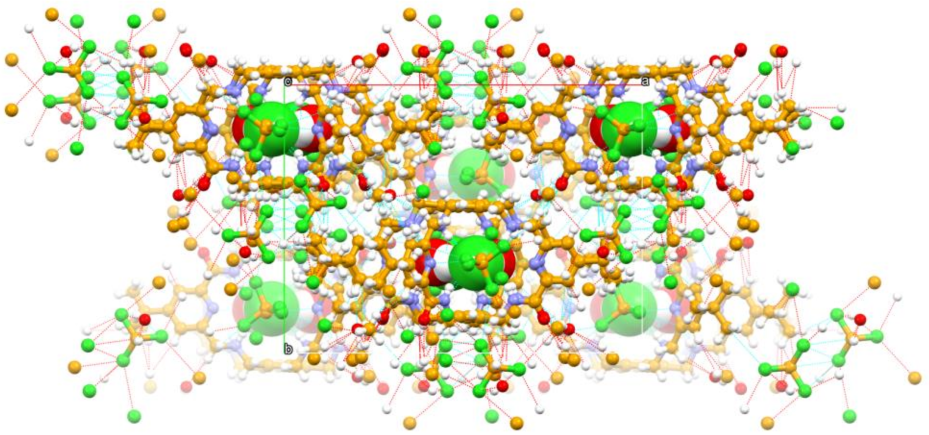

Supplementary Fig. 108 | The coordination network in the complex of $2H_2O{\cdot}2Cl^{-}{\subset}\mathbf{8}{\cdot}2H^{+}$ with chloroform filling in the lattice.

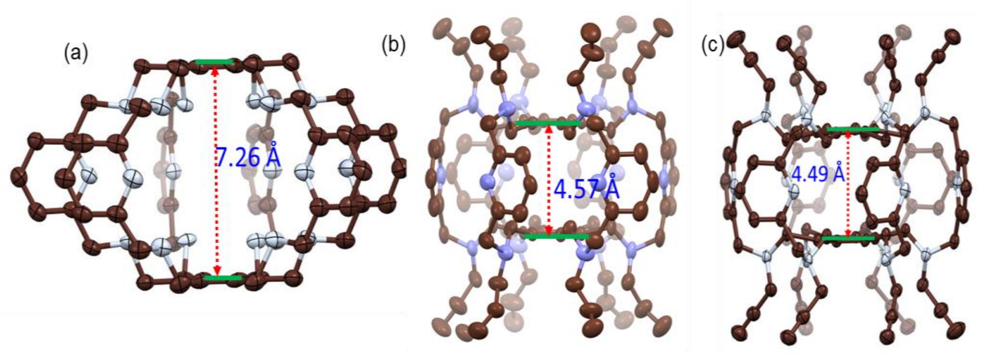

Supplementary Fig. 109 | Single–crystal structures of three reported superphane structures from The Cambridge Cambridge Structural Data Centre. (a) CCDC number: 2108725; (b) CCDC number: 2108722 and (c) CCDC number: 2108724.[24] In (a) the two benzene rings on the bottom and the top of the superphane cage reside outside the cavity with a distance of 7.26 Å. Instead, after functionalization with allyl group or propargyl, the two benzene rings in question self–fill the internal cavities, giving a distance of 4.57 Å and 4.49 Å for (b) and (c), resepctively. In both (b) and (c), the internal cavity disappeared via shrinking. This indicates the potential self–filling ability of the superphane cage.

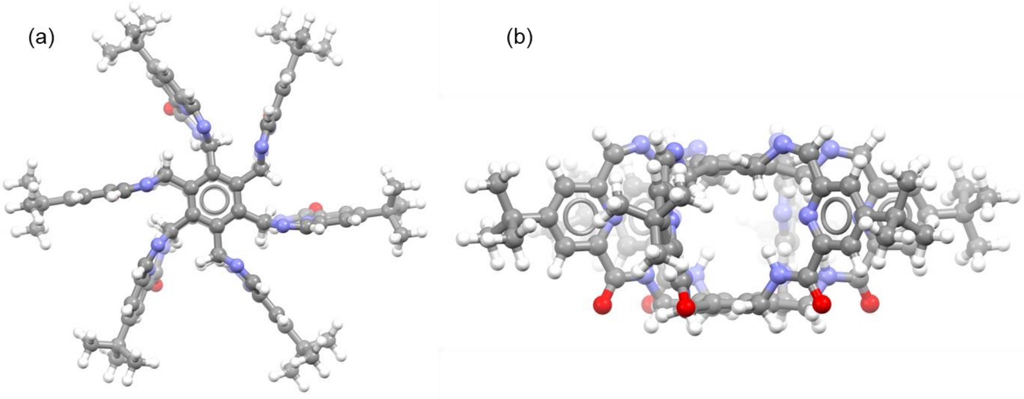

Supplementary Fig. 110 | (a) Top view and (b) front view of the DFT–optimized structure of **SUPE–py–Imine–Cage** in a shrinking conformation with one benzene ring self–filling the internal cavity. The distance between two benzene rings on the top and on the bottom is only 5.50 Å, revealing invalid void within the cage.

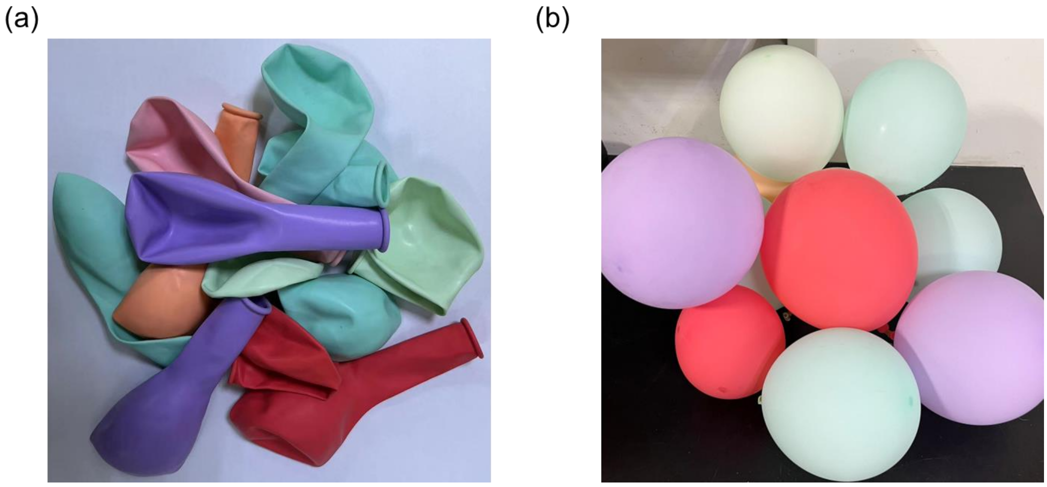

Supplementary Fig. 111 | Images of (a) the flat balloons and (b) the blowing balloons. The air blown into the balloon induces the swelling of the balloon, simultaneously generating more voids between the blowing balloons. This might be similar to the iodine uptake process with the nonporous amorphous superphane molecular materials.

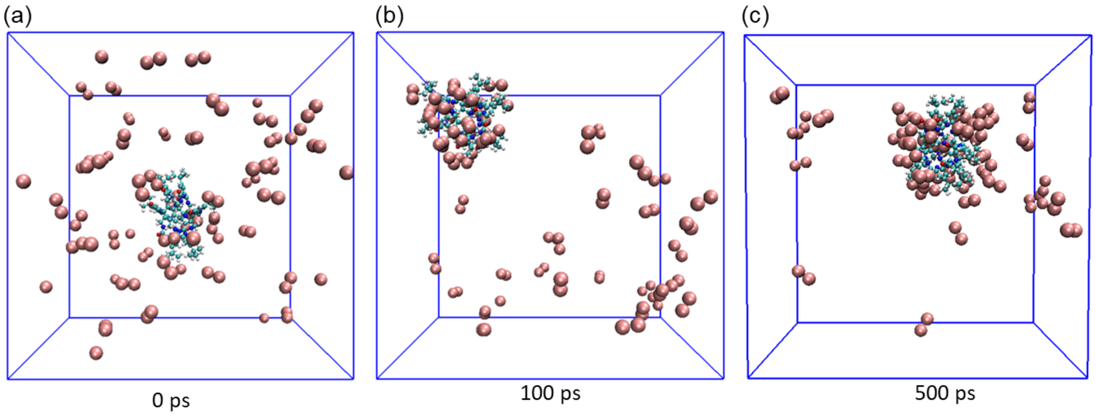

Supplementary Fig. 112. Snapshots of molecular dynamics run on the superphane **SUPE–py–Imine–Cage** and I_2 in the gas phase using periodic boundary conditions at 298.15 K at (a) 0 ps, (b) 100 ps and (c) 500 ps. Note: I_2 : **SUPE–py–Imine–Cage** = 50 : 1.

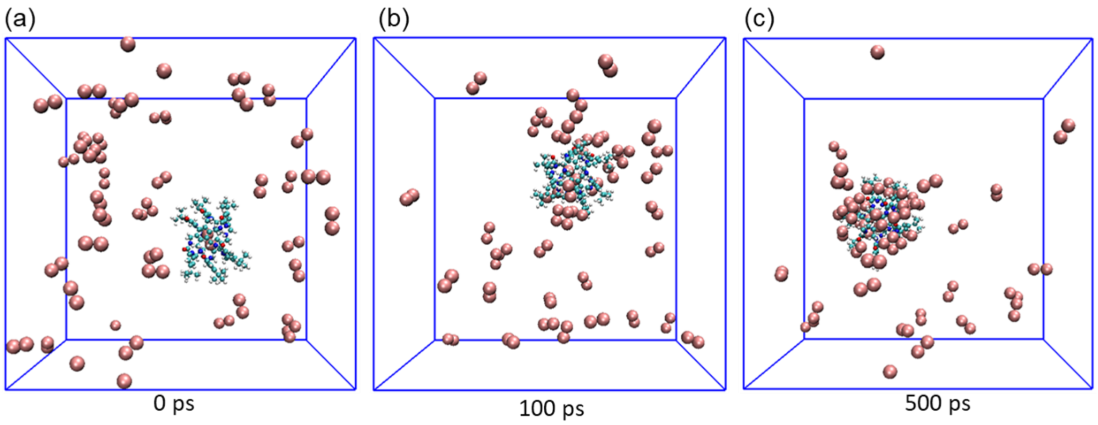

Supplementary Fig. 113. Snapshots of molecular dynamics run on the superphane **SUPE–py–Amine–Cage** and I_2 in the gas phase using periodic boundary conditions at 298.15 K at (a) 0 ps, (b) 100 ps and (c) 500 ps. Note: I_2 : **SUPE–py–Amine–Cage** = 42 : 1.

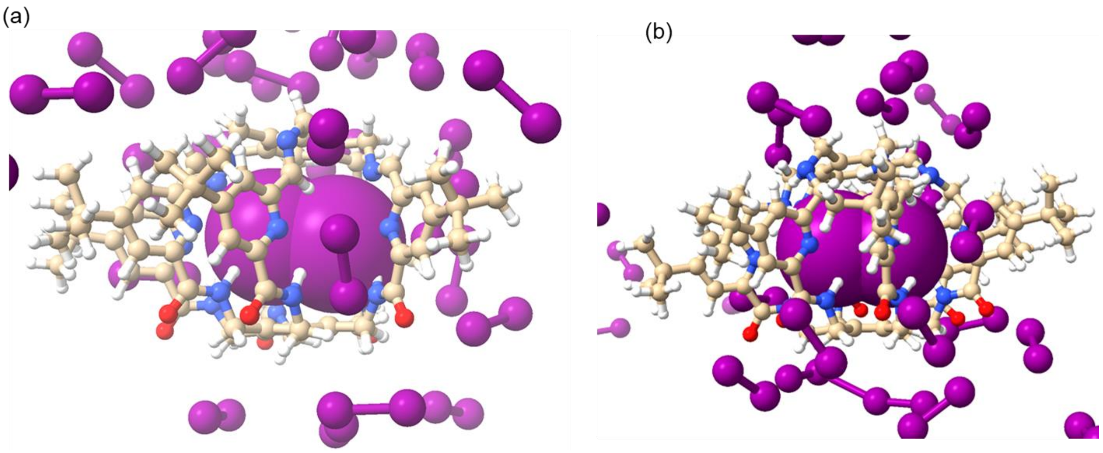

Supplementary Fig. 114. Snapshots of molecular dynamics run (at 500 ps) on the superphane (a) **SUPE–py–Imine–Cage** and (b) **SUPE–py–Amine–Cage** with iodine in the gas phase using periodic boundary conditions at 298.15 K. These results indicated the hydrogen binding between the trapped iodine and the cages.

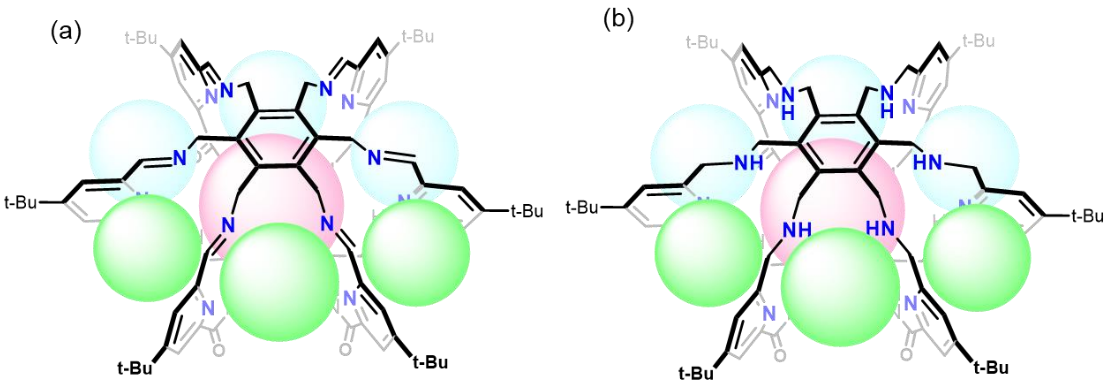

Supplementary Fig. 115 | Possible iodine binding domains of the central cavities (in pink) and outer apertures (in green) for (a) **SUPE–py–Imine–Cage** and (b) **SUPE–py–Amine–Cage**.

Supplementary Note 7. X–ray experimental details

X–ray experimental for SUPE–py–Imine–Cage

Single crystals of **SUPE–py–Imine–Cage** were obtained as yellowish blocks via vapor diffusion of tetrahydrofuran (THF) into a $CHCl_3$ solution of **SUPE–py–Imine–Cage**. A suitable crystal was selected and the data were collected on Agilent SuperNova system equipped with a mirror monochromator and a Cu–Kα INCOATEC IμS micro focus source (λ = 1.54184 Å). The crystal was kept at 293(2) K during data collection. Using Olex2,[25] the structure was solved with the ShelXT[26] structure solution program using direct methods and refined with the ShelXL[27] refinement package using least squares minimization. Tables of positional and thermal parameters, bond lengths and angles, torsion angles and figures are in the CIF file. CCDC number: 2245537.

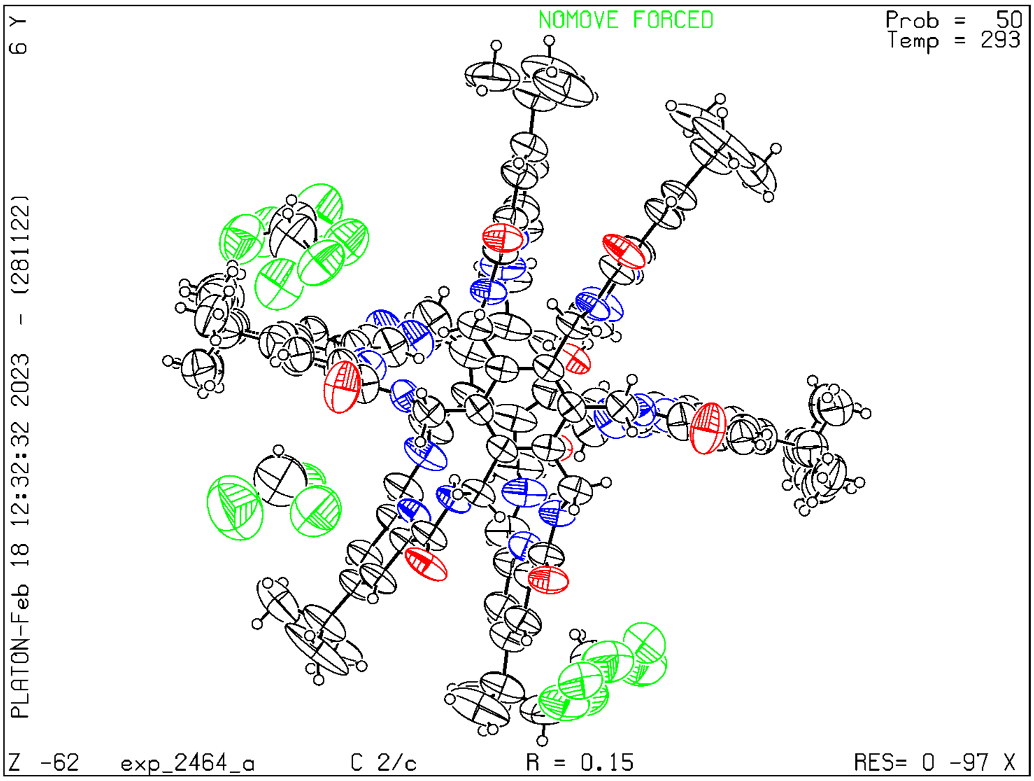

Supplementary Fig. 116 | View of the complex **SUPE–py–Imine–Cage**. Displacement ellipsoids are scaled to the 50% probability level

Supplementary Table 7 | Crystal data and structure refinement for SUPE–py–imine–Cage.

Identification code	**SUPE–py–Imine–Cage**
Empirical formula	$C_{185} H_{217} Cl_{15} N_{36} O_{20}$
Formula weight	3796.70
Temperature/K	293(2)
Crystal system	monoclinic
Space group	C2/c
a/Å	22.321(5)
b/Å	16.471(3)
c/Å	29.802(6)
α/°	90
β/°	91.85(3)
γ/°	90
Volume/Å^3	10951(4)
Z	2
ρcalcg/cm^3	1.151
μ/mm-1	0.252
F(000)	3988.0
Crystal size/mm^3	0.24 × 0.23 × 0.2
Radiation	MoKα (λ = 0.71073 Å)
2Θ range for data collection/°	3.074 to 48.814
Index ranges	$-24 \leq h \leq 25$, $-13 \leq k \leq 19$, $-32 \leq l \leq 34$
Reflections collected	17953
Independent reflections	8983 [R_{int} = 0.0364, R_{sigma} = 0.0602]
Data/restraints/parameters	8983/1251/791
Goodness–of–fit on F^2	0.906
Final R indexes [I>=2σ (I)]	R_1 = 0.1501, wR_2 = 0.3225
Final R indexes [all data]	R_1 = 0.2167, wR_2 = 0.3640
Largest diff. peak/hole / e Å^{-3}	0.49/–0.34
CCDC number	2245537

X–ray experimental for SUPE–py–Amine–Cage

Single crystals of **SUPE–py–Amine–Cage** were obtained as colourless needle via slowly evaporating of the chloroform solution of **SUPE–py–Amine–Cage**. A suitable crystal was selected and the data were collected on a Bruker APX system equipped with a mirror monochromator and a Cu–Kα INCOATEC IμS micro focus source (λ = 1.54178 Å). The crystal was kept at 186 K during data collection. Using Olex2,[25] the structure was solved with the ShelXT[26] structure solution program using direct methods and refined with the ShelXL[27] refinement package using least squares minimization. Tables of positional and thermal parameters, bond lengths and angles, torsion angles and figures are in the CIF file. CCDC number: 2245538.

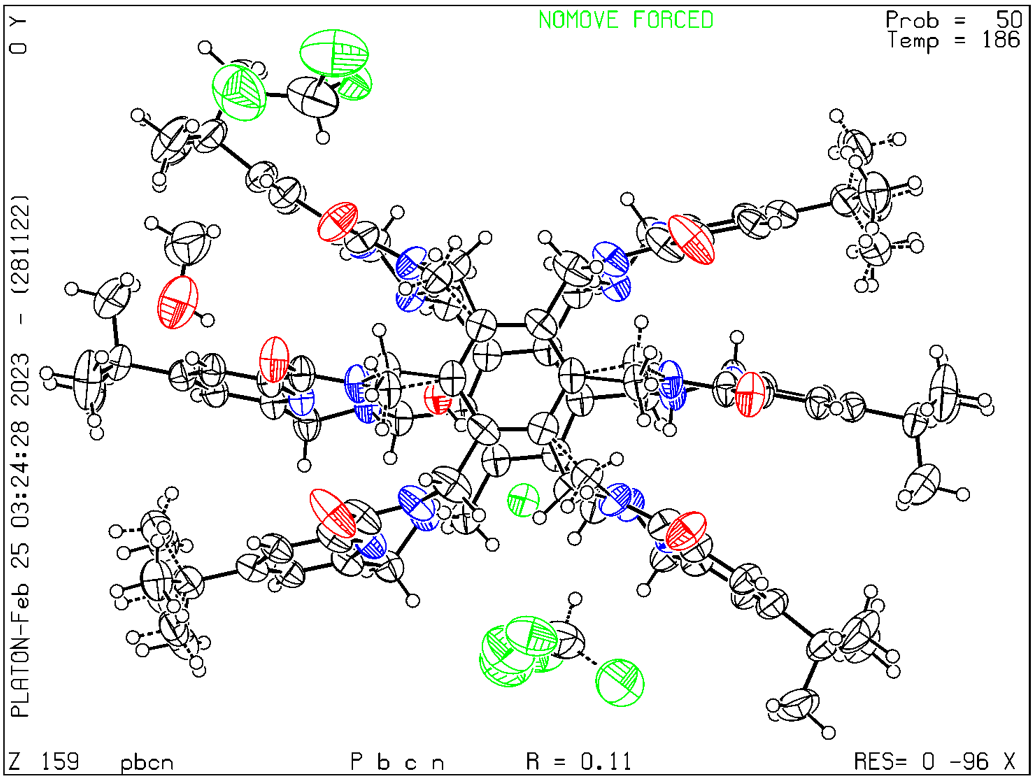

Supplementary Fig. 117 | View of the complex **SUPE–py–Amine–Cage**. Displacement ellipsoids are scaled to the 50% probability level

Supplementary Table 8 | Crystal data and structure refinement for SUPE–py–Amine–Cage.

Identification code	**SUPE–py–Amine–Cage**
Empirical formula	$C_{96}H_{132}Cl_{14}N_{18}O_{10}$
Formula weight	2194.49
Temperature/K	186.0
Crystal system	orthorhombic
Space group	Pbcn
a/Å	22.469(6)
b/Å	16.829(4)
c/Å	28.316(7)
α/°	90
β/°	90
γ/°	90
Volume/Å^3	10707(5)
Z	4
ρcalcg/cm^3	1.361
μ/mm–1	3.819
F(000)	4608.0
Crystal size/mm^3	0.31 × 0.2 × 0.17
Radiation	CuKα (λ = 1.54178 Å)
2Θ range for data collection/°	7.268 to 132.516
Index ranges	$-22 \leq h \leq 26$, $-11 \leq k \leq 19$, $-28 \leq l \leq 33$
Reflections collected	41825
Independent reflections	9256 [R_{int} = 0.1102, R_{sigma} = 0.0714]
Data/restraints/parameters	9256/707/731
Goodness–of–fit on F^2	1.068
Final R indexes [I>=2σ (I)]	R_1 = 0.1054, wR_2 = 0.2537
Final R indexes [all data]	R_1 = 0.1685, wR_2 = 0.2888
Largest diff. peak/hole / e Å^{-3}	0.67/–1.02
CCDC number	2245538

Supplementary Note 8. HRMS spectra and NMR spectra

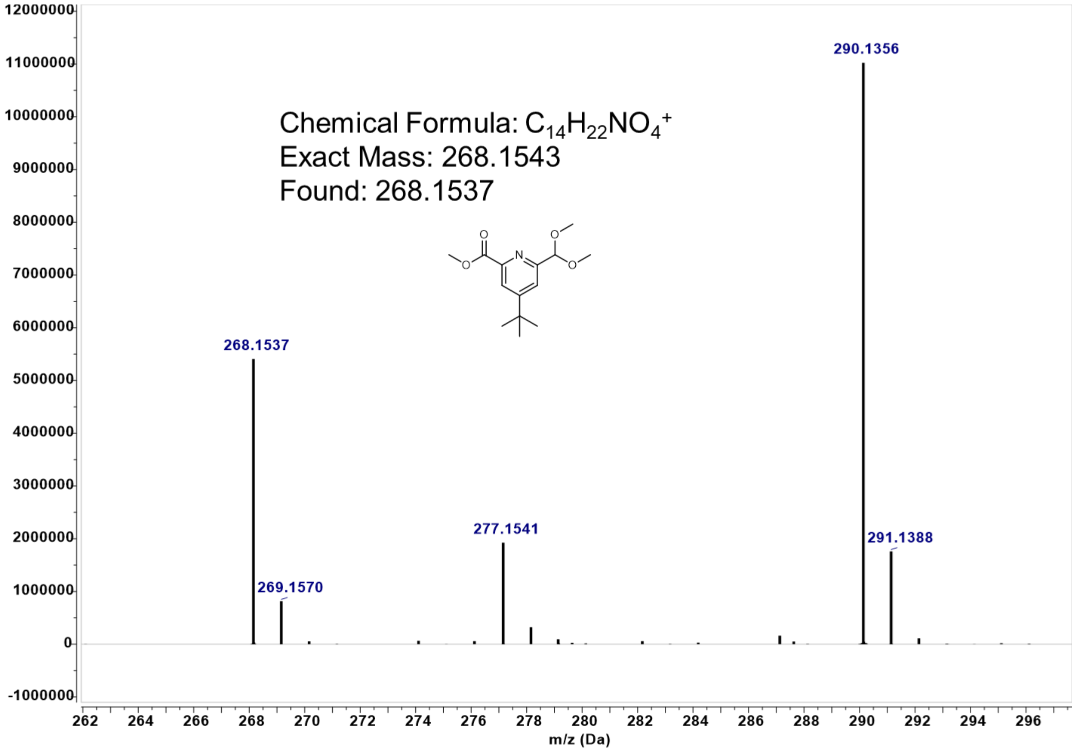

Supplementary Fig. 118 | HRMS spectrum of compound **2**.

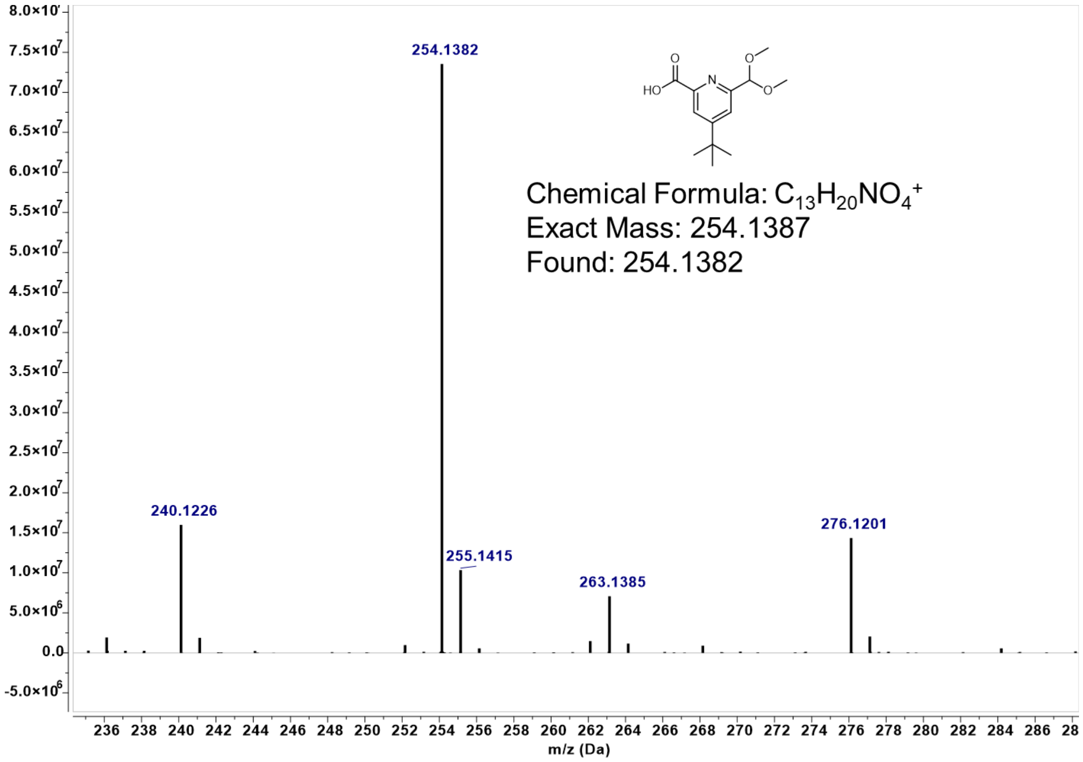

Supplementary Fig. 119 | HRMS spectrum of compound **3**.

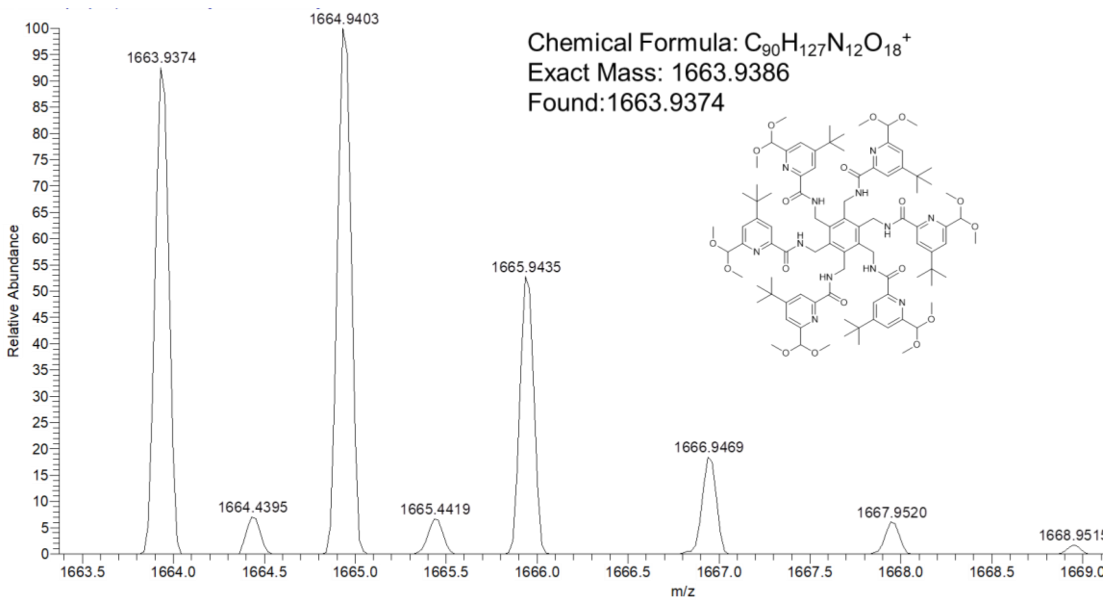

Supplementary Fig. 120 | HRMS spectrum of compound **4**.

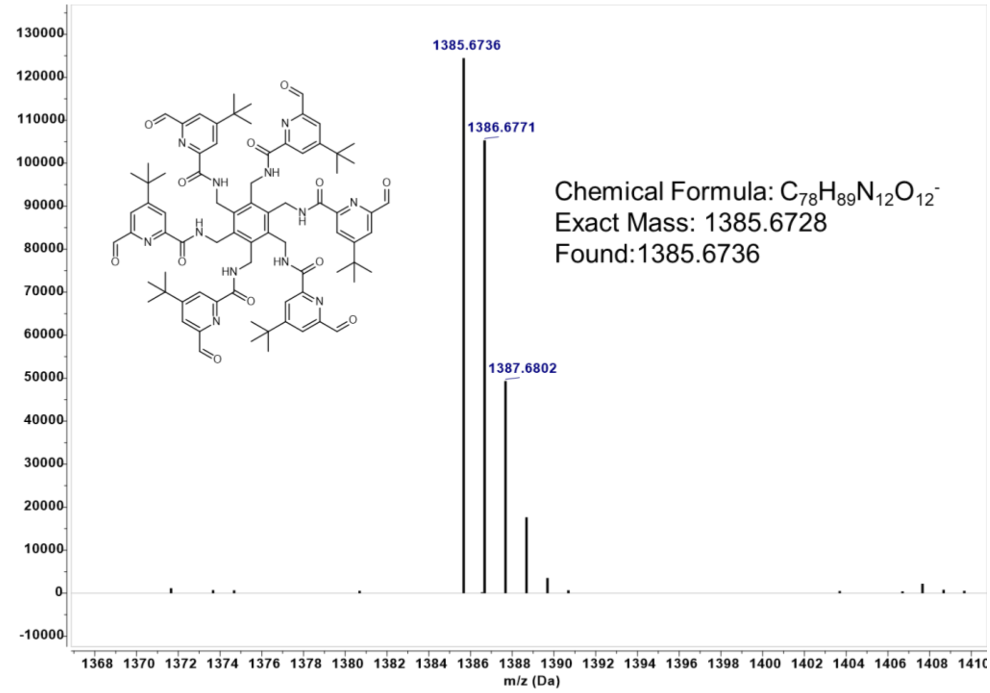

Supplementary Fig. 121 | HRMS spectrum of compound **5**.

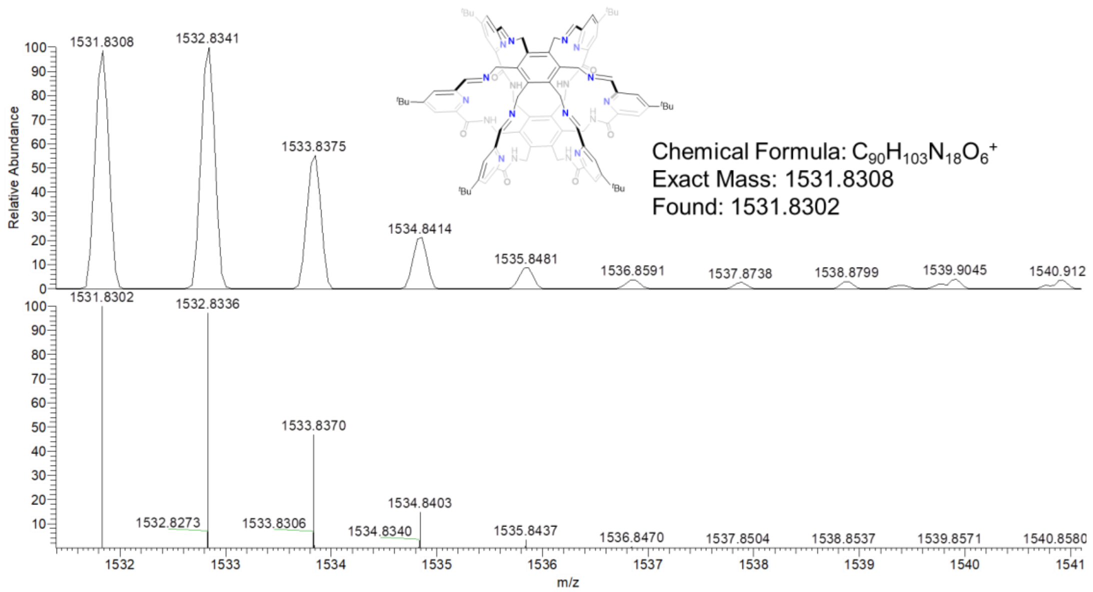

Supplementary Fig. 122 | HRMS spectrum of compound **SUPE–py–Imine–Cage (7)**.

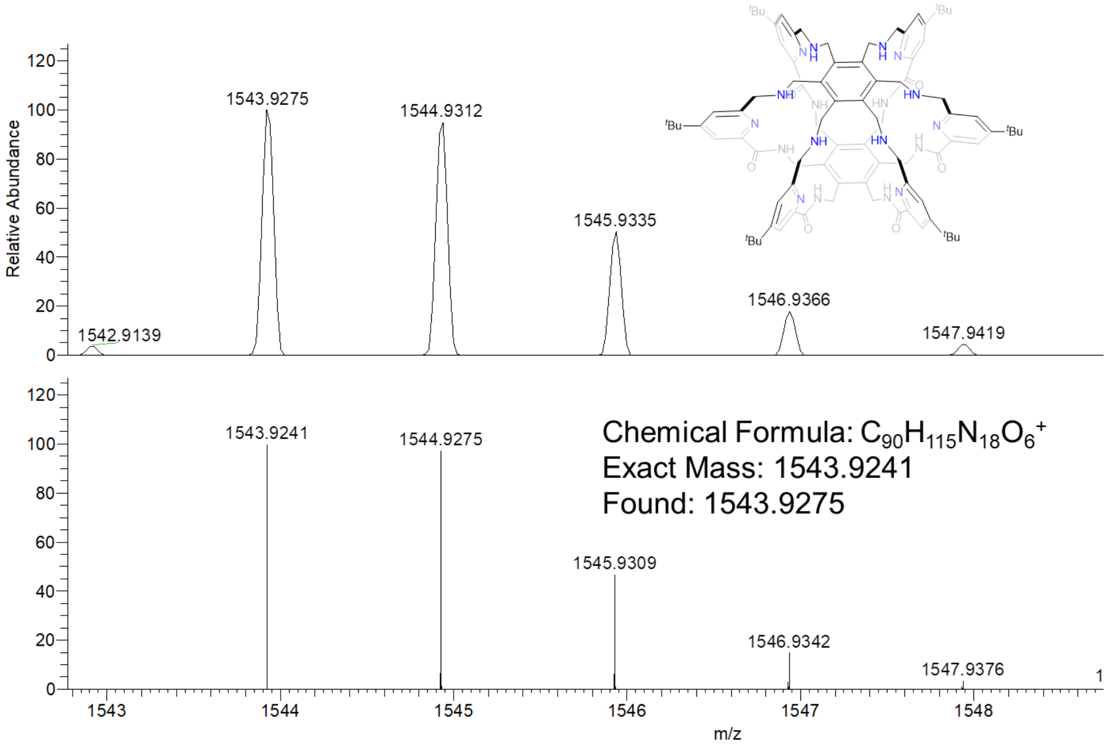

Supplementary Fig. 123 | HRMS spectrum of compound **SUPE–py–Amine–Cage (8)**.

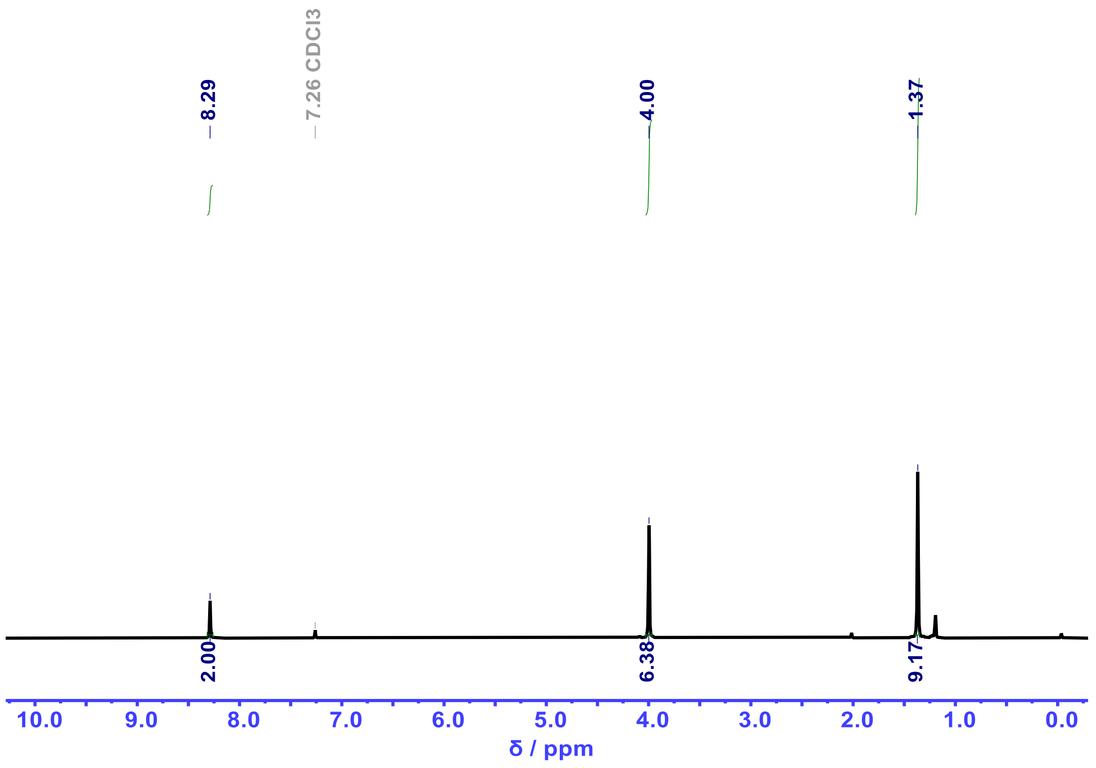

Supplementary Fig. 124 | ^{1}H NMR spectrum of **S1** recorded in $CDCl_3$.

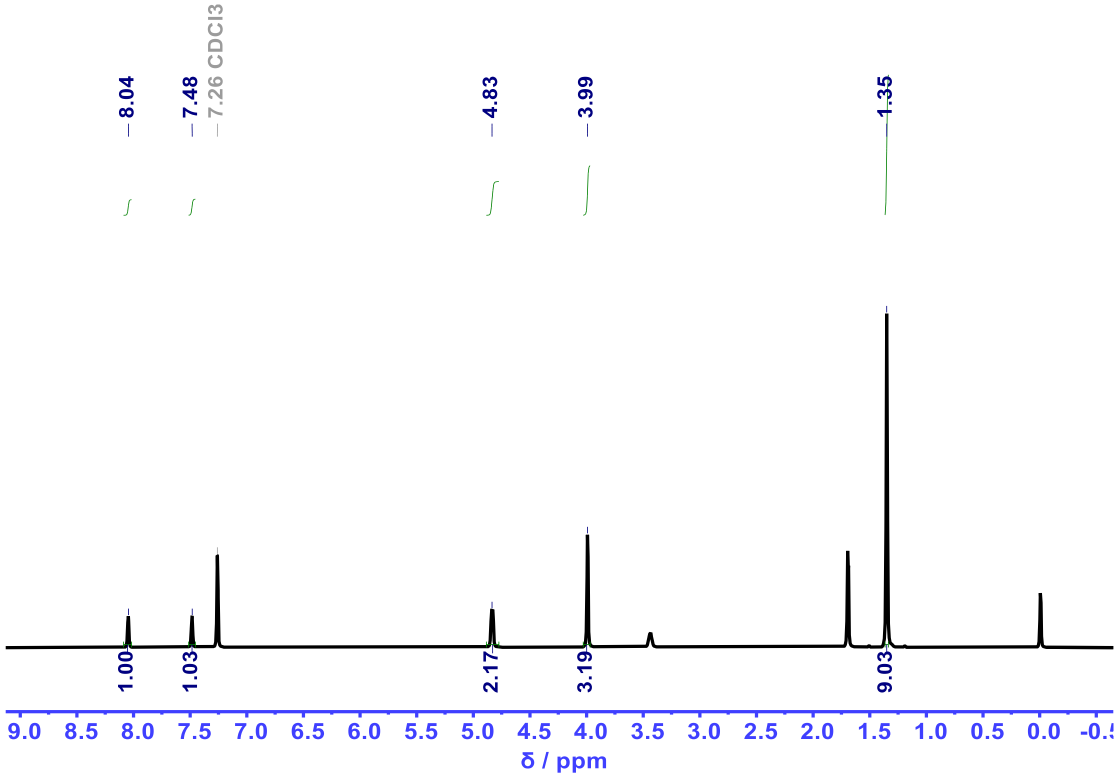

Supplementary Fig. 125 | ^{1}H NMR spectrum of **S2** recorded in $CDCl_3$.

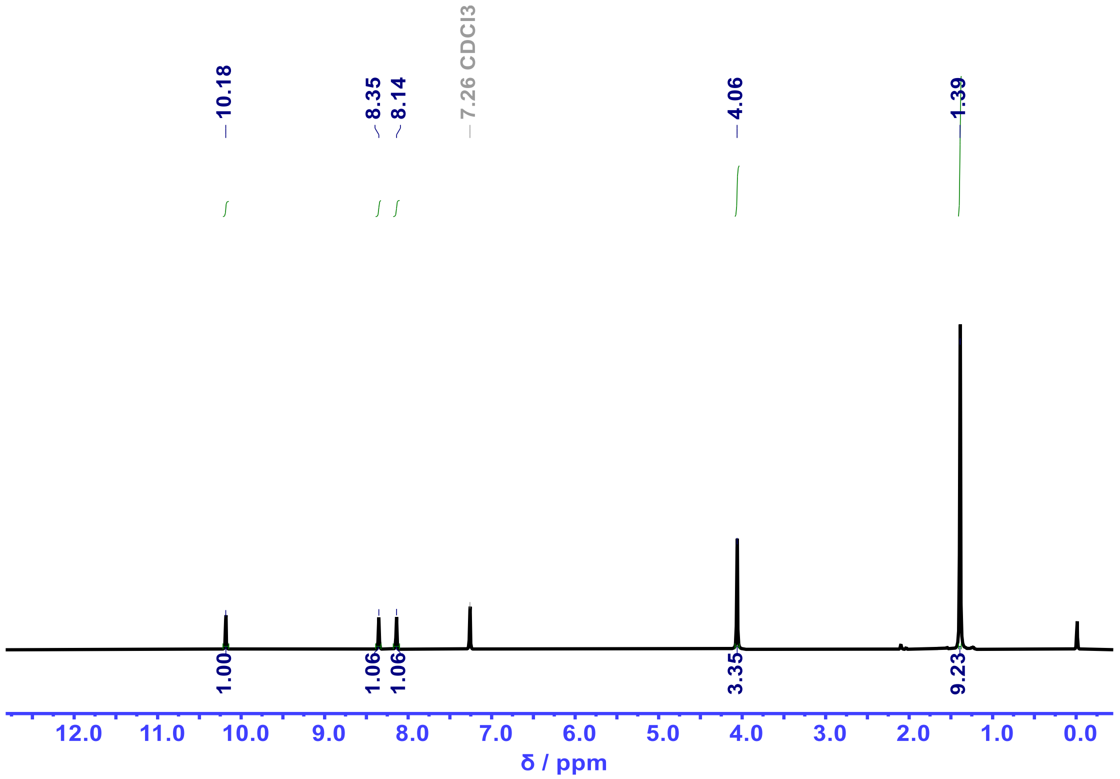

Supplementary Fig. 126 | ^{1}H NMR spectrum of **1** recorded in $CDCl_3$.

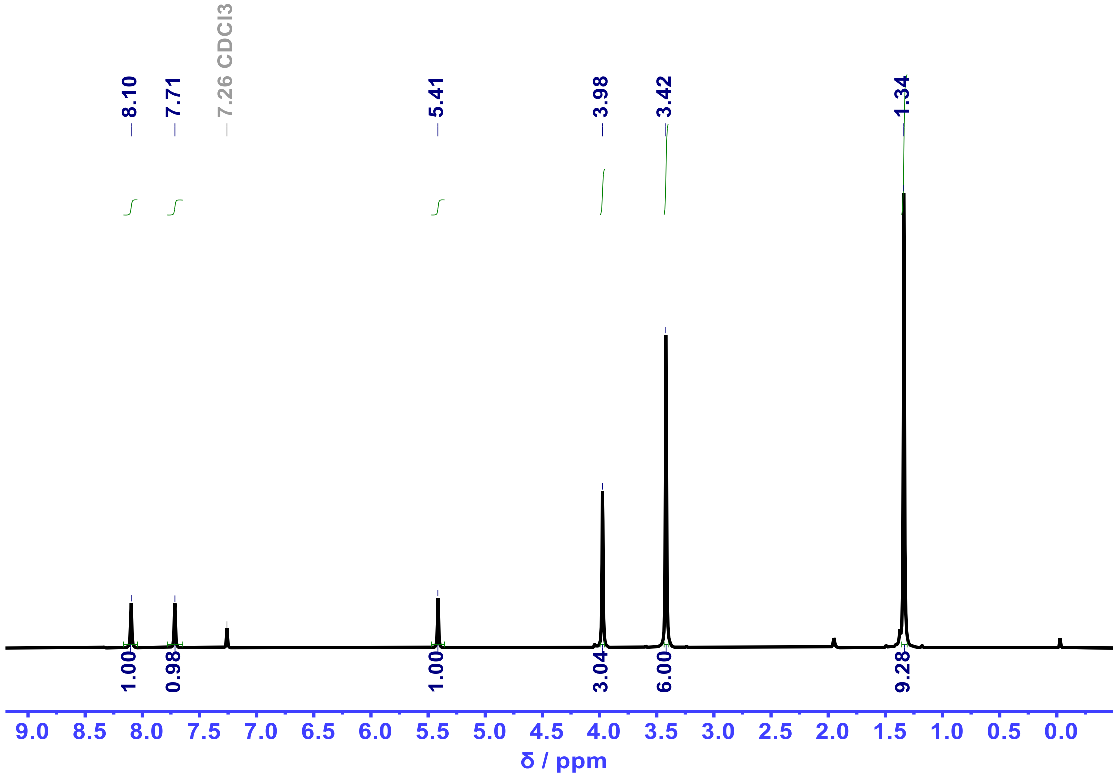

Supplementary Fig. 127 | ^{1}H NMR spectrum of **2** recorded in $CDCl_3$.

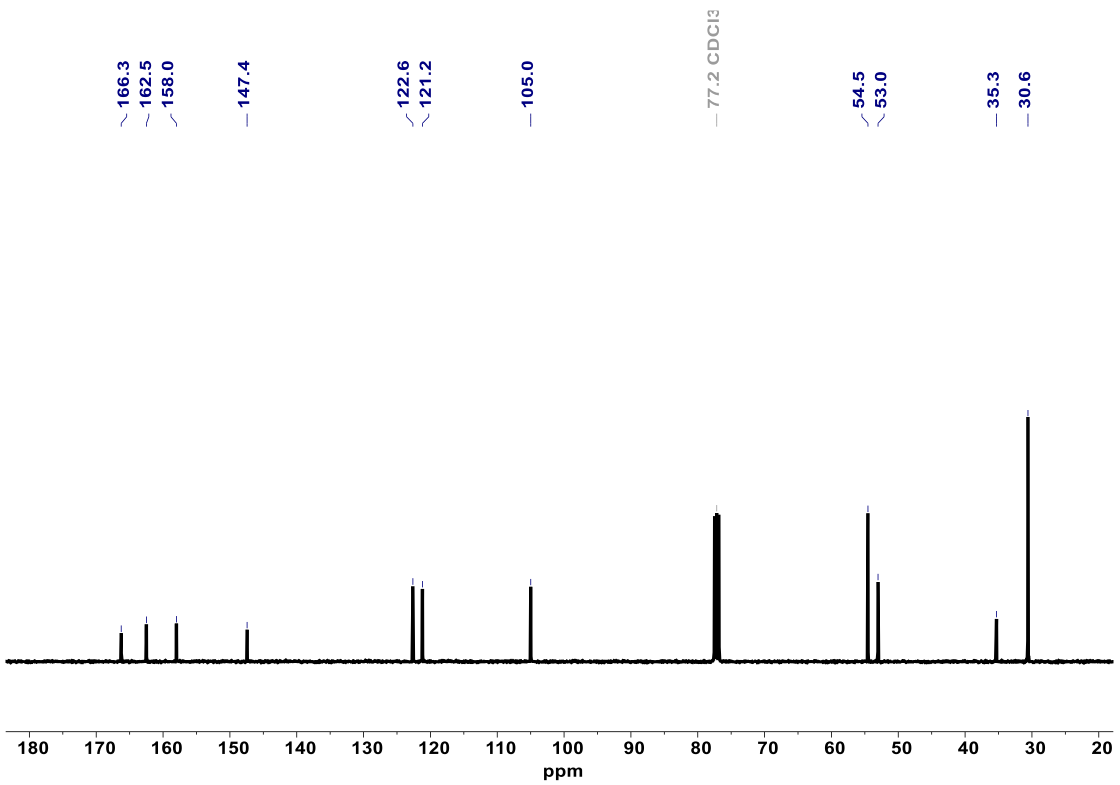

Supplementary Fig. 128 | ^{13}C NMR spectrum of **2** recorded in $CDCl_3$.

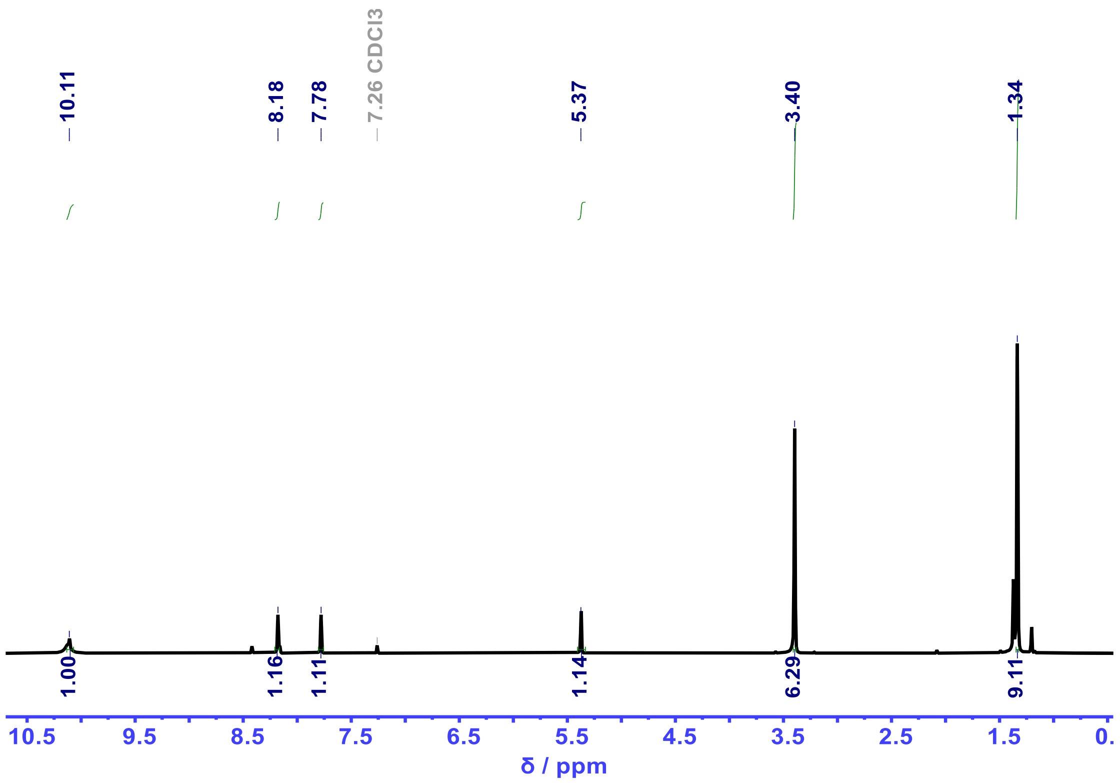

Supplementary Fig. 129| ^{1}H NMR spectrum of **3** recorded in $CDCl_3$.

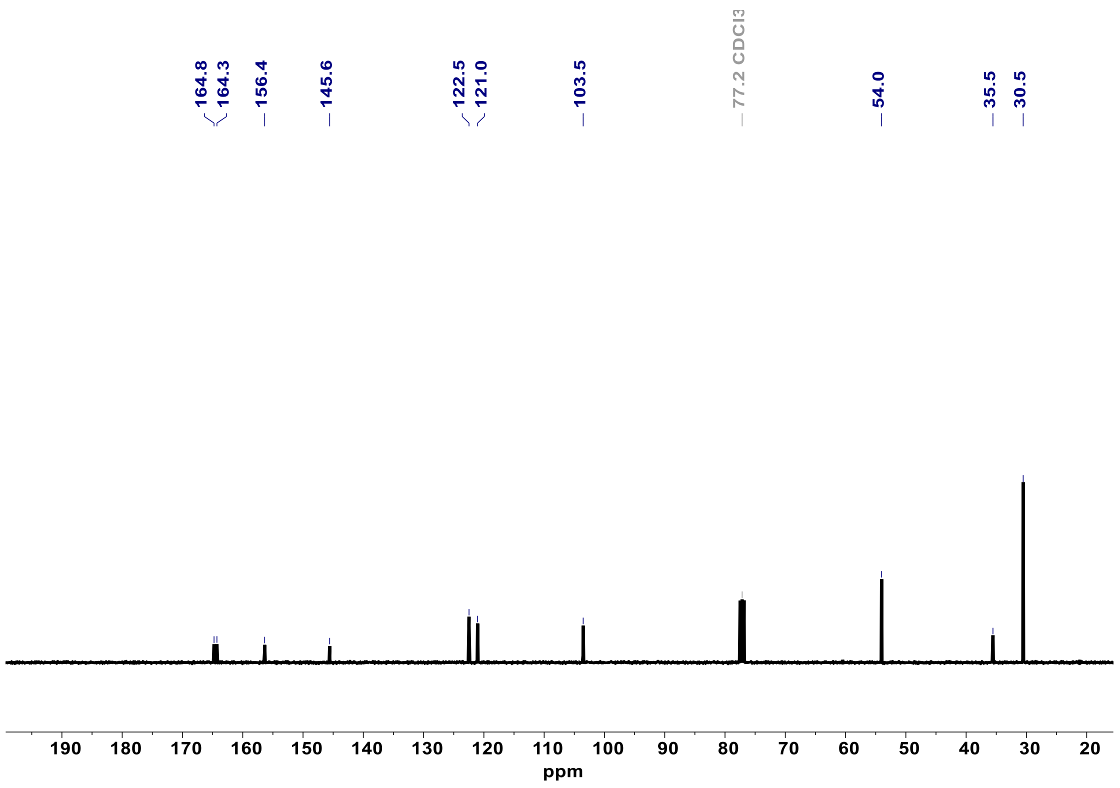

Supplementary Fig. 130 | ^{13}C NMR spectrum of **3** recorded in $CDCl_3$.

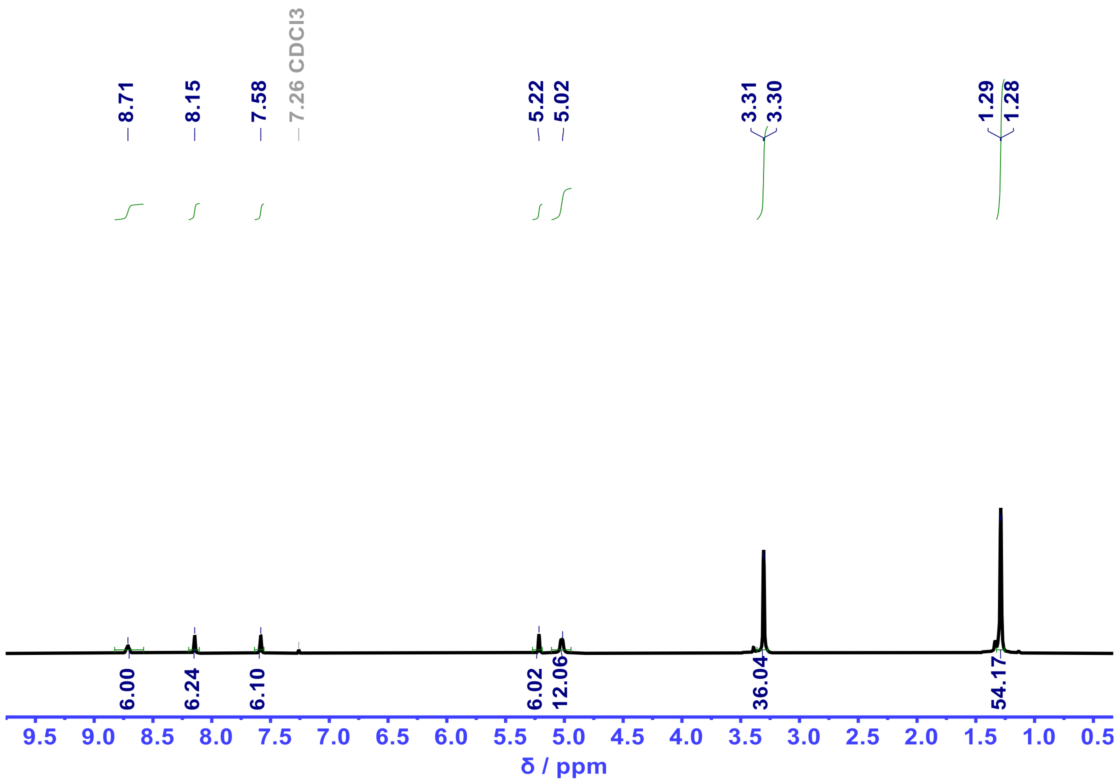

Supplementary Fig. 131 | ^{1}H NMR spectrum of **5** recorded in $CDCl_3$.

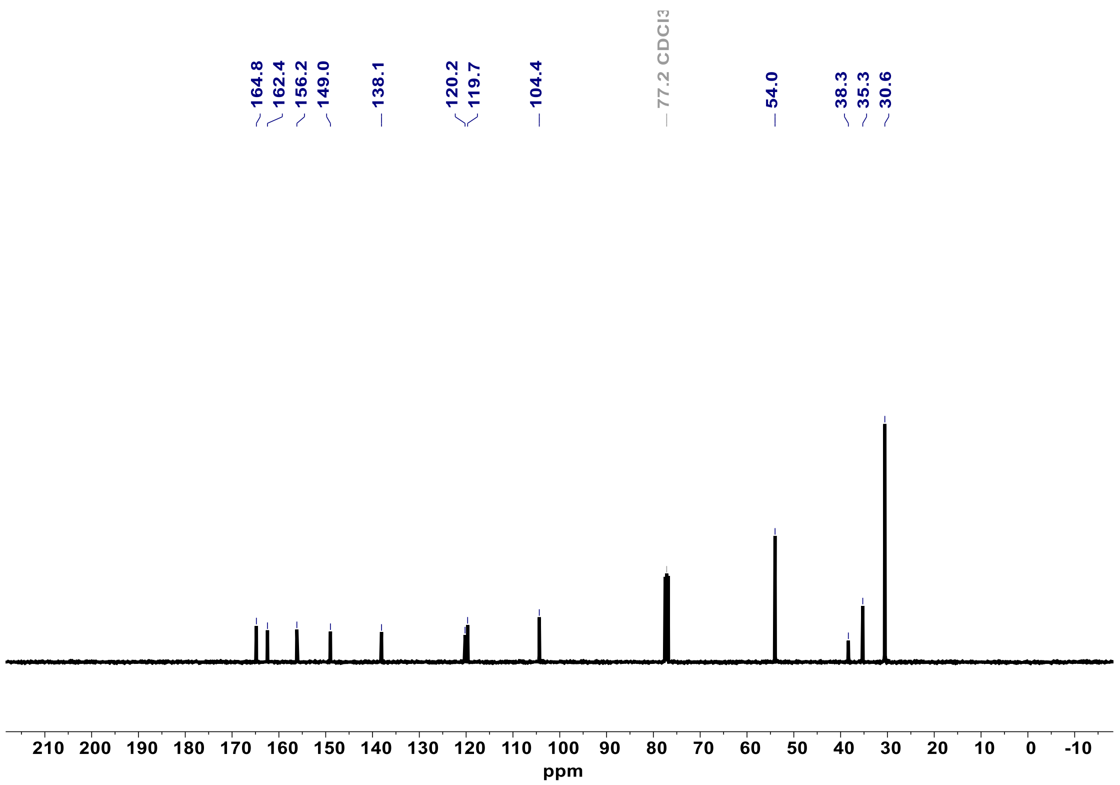

Supplementary Fig. 132 | ^{13}C NMR spectrum of **5** recorded in $CDCl_3$.

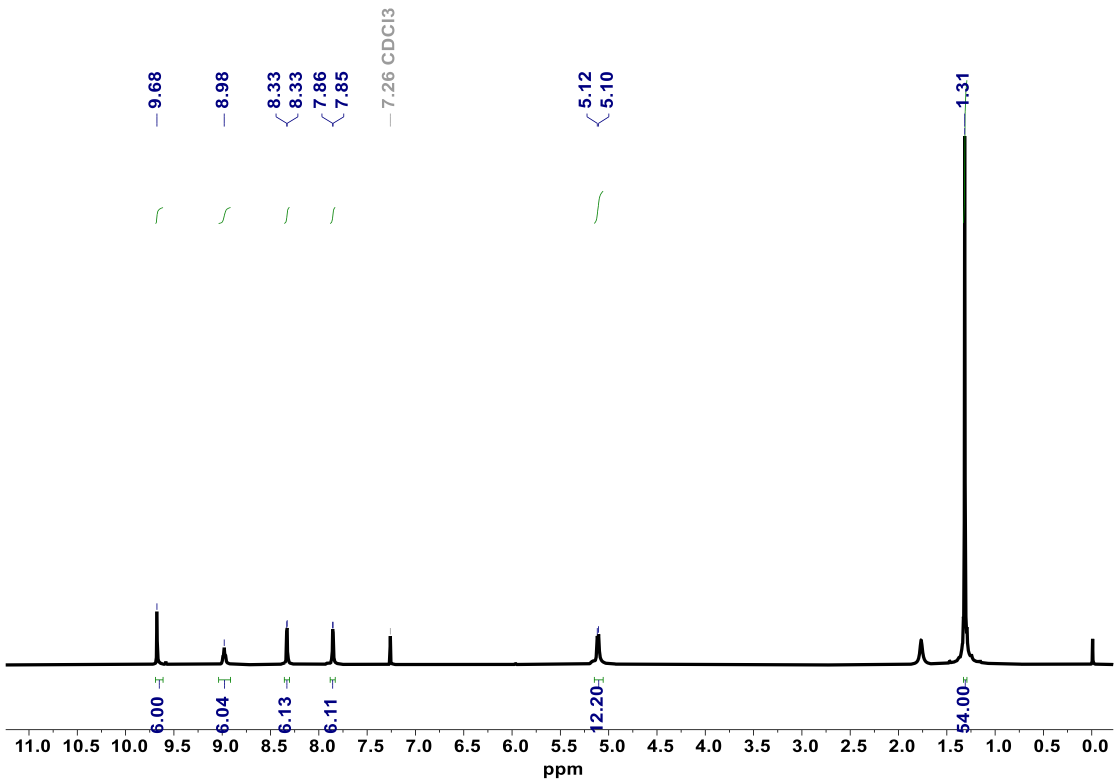

Supplementary Fig. 133 | ^{1}H NMR spectrum of **6** recorded in $CDCl_3$.

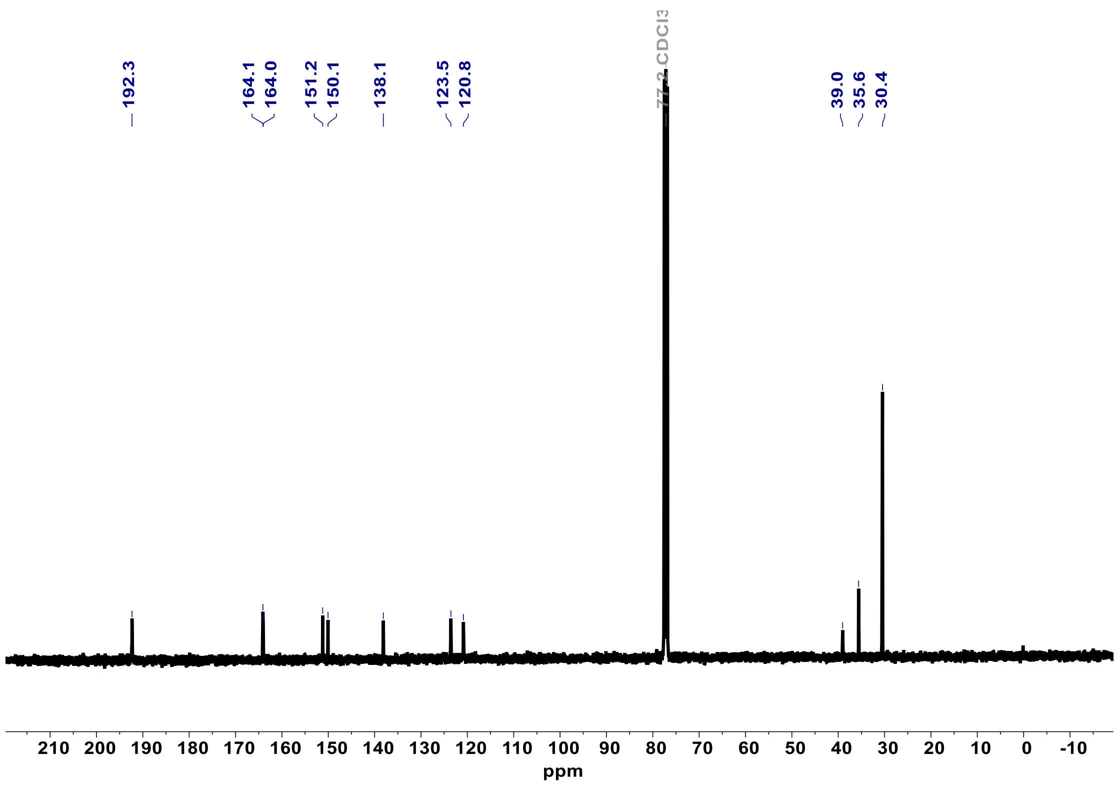

Supplementary Fig. 134 | ^{13}C NMR spectrum of **6** recorded in $CDCl_3$.

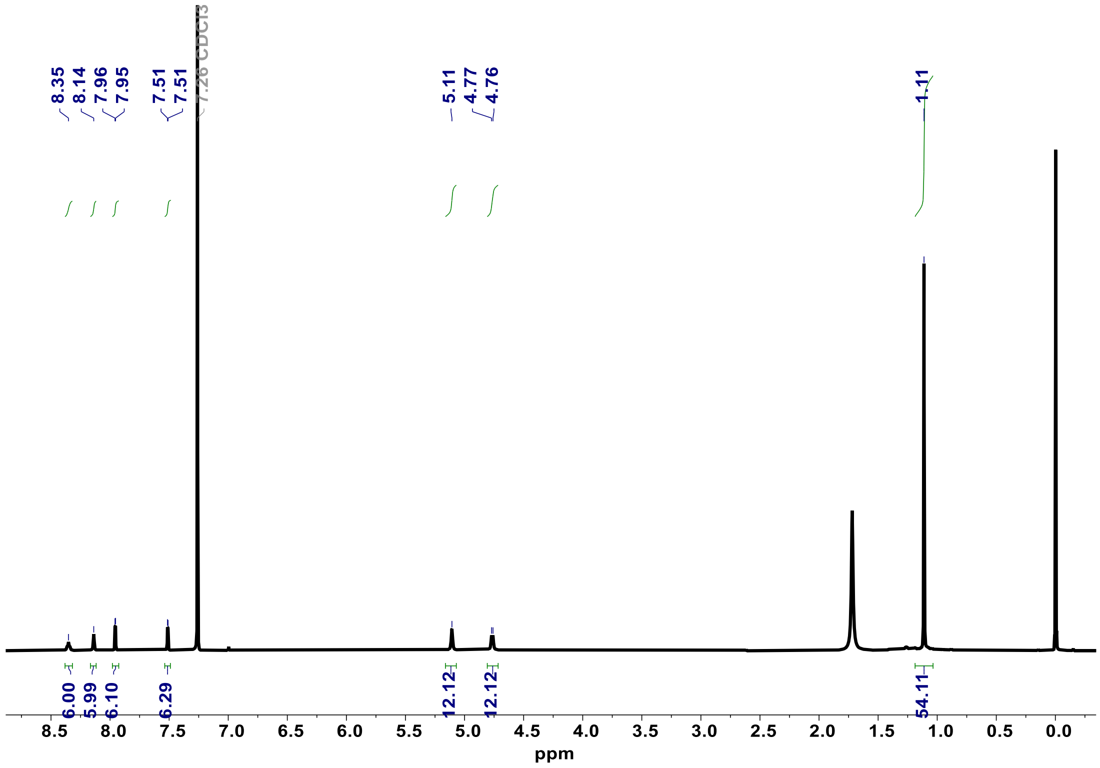

Supplementary Fig. 135 | ^{1}H NMR spectrum of **SUPE–py–Imine–Cage (7)** recorded in $CDCl_3$.

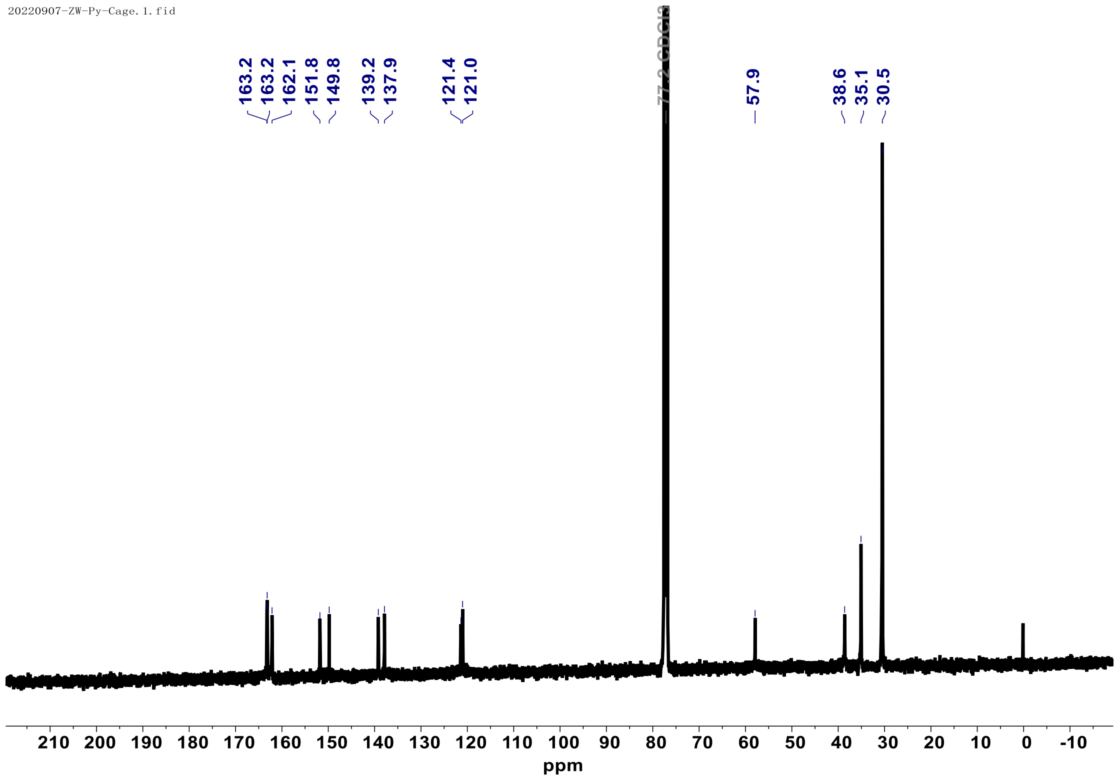

Supplementary Fig. 136 | ^{13}C NMR spectrum of **SUPE–py–Imine–Cage (7)** recorded in $CDCl_3$.

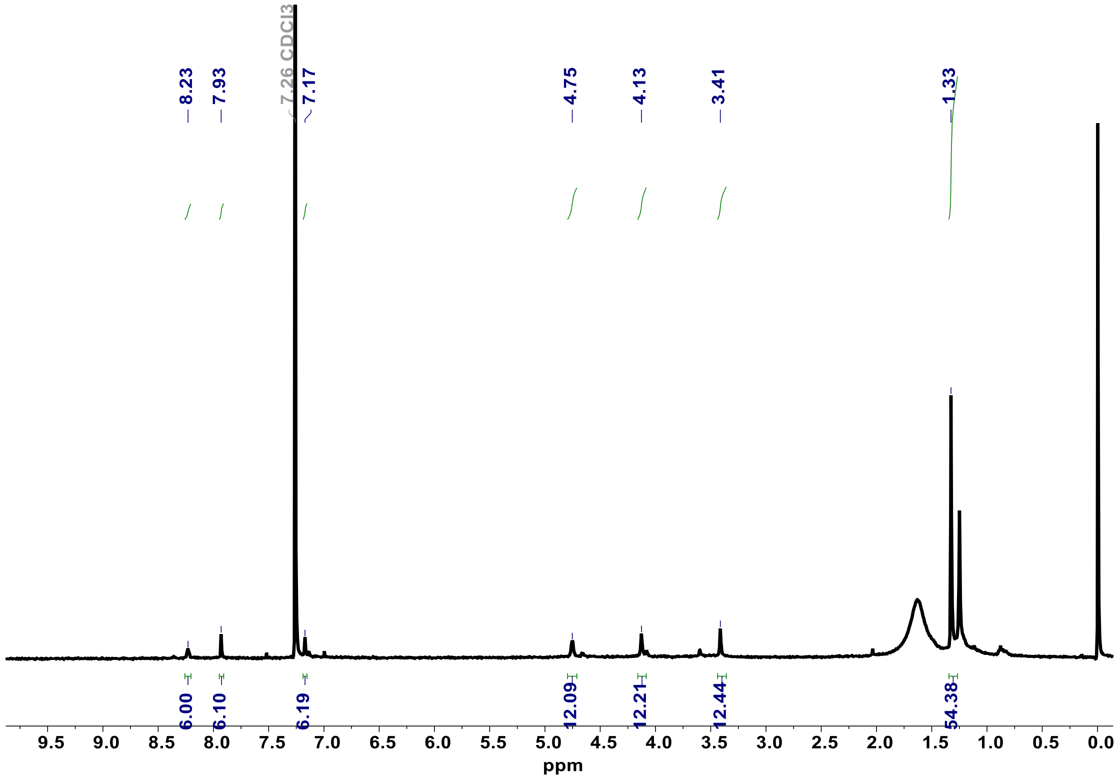

Supplementary Fig. 137 | ^{1}H NMR spectrum of **SUPE–py–Amine–Cage (8)** contain I_2 (10 eq) recorded in $CDCl_3$.

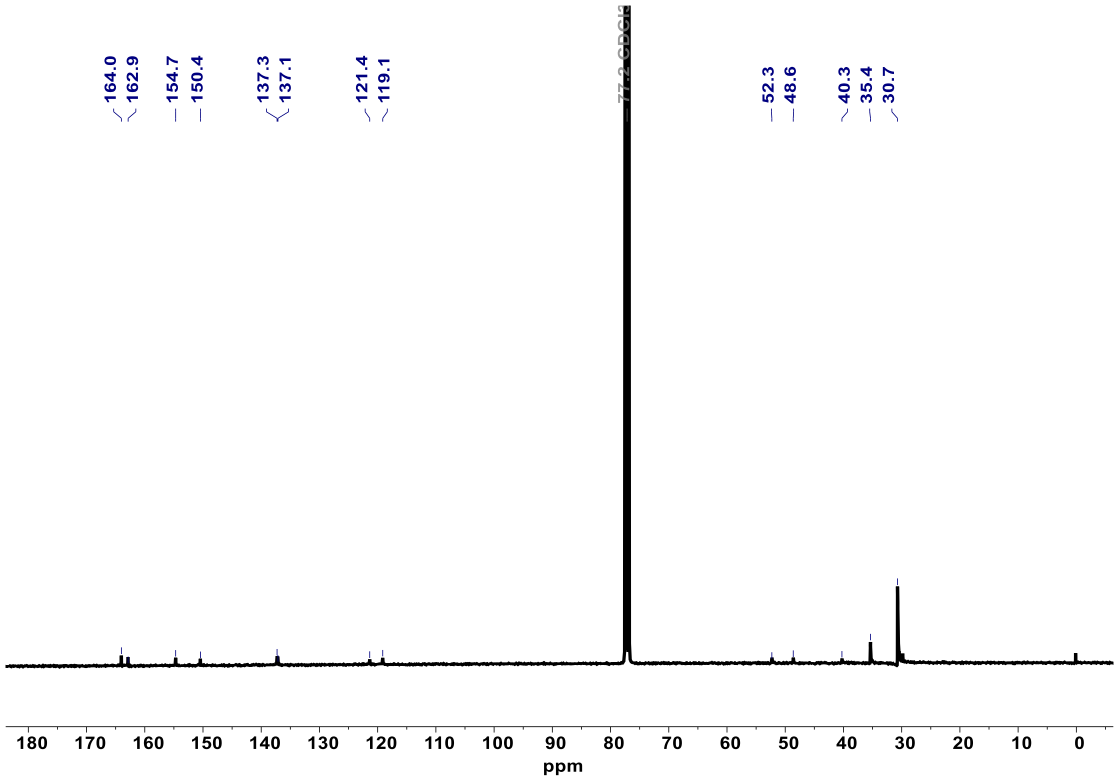

Supplementary Fig. 138 | ^{13}C NMR spectrum of **SUPE–py–Amine–Cage (8)** contain I_2 (10 eq) recorded in $CDCl_3$.

Supplementary Note 9. Energies and geometrical coordinates of the optimized models in the gas phase

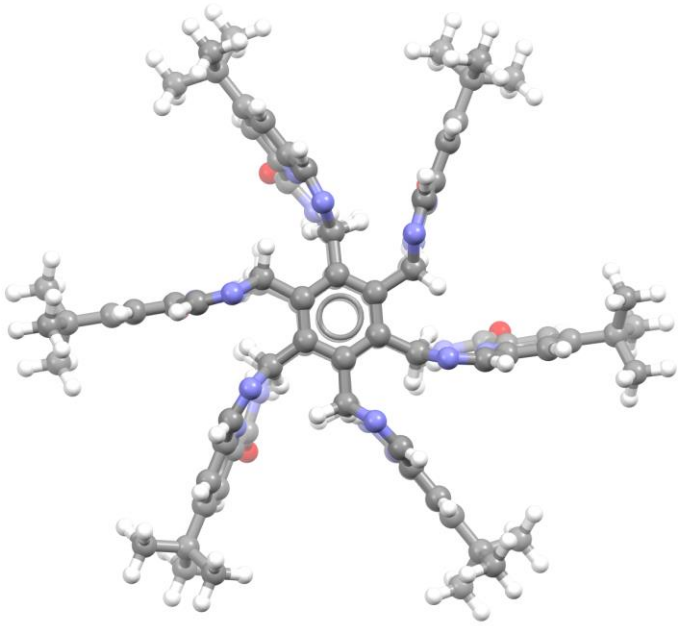

Supplementary Fig. 139 | Top view of DFT-optimized **SUPE–py–Imine–Cage**.

Cartesian coordinates

Symbol	X	Y	Z
C	1.316495	0.513574	–2.389098
C	–1.310018	–0.498858	–2.426702
N	–2.819509	2.656119	–2.982898
N	–3.677829	–0.467141	–3.24526
N	–0.879711	–3.764972	–2.994514
N	–1.074148	–3.529197	2.054723
N	–2.517914	2.685306	2.067579
C	2.686845	1.004059	–1.941592
H	3.020529	0.348863	–1.132741
H	2.582236	1.982932	–1.466671
C	0.224651	1.393478	–2.42042
C	1.096314	–0.882005	–2.424636
N	–1.783005	–4.742878	–0.266407
N	3.711501	1.125758	–2.977603
N	–3.215259	3.915933	–0.246811
C	–0.21191	–1.387741	–2.39535
C	–0.472935	–2.821695	–1.954332
H	0.426805	–3.222978	–1.480804
H	–1.20763	–2.786973	–1.145275
C	–1.09417	0.88684	–2.391385
C	–0.51299	–2.898411	3.248912
H	–1.230054	–3.090524	4.04976
H	0.397849	–3.427608	3.53265
C	–2.250906	1.878955	3.258046
H	–2.059097	2.592456	4.062199
H	–3.164088	1.352812	3.539488
C	–2.716335	–1.095847	–2.347089
H	–3.05718	–1.143739	–1.307397
H	–2.659579	–2.121956	–2.71988
N	5.002367	0.82574	–0.24283
C	–2.205794	1.82751	–1.946164
H	–3.003031	1.246024	–1.475959
H	–1.80869	2.442554	–1.134149
C	–4.040151	6.62192	–0.307283
C	–2.411643	–5.151335	0.841752
C	7.757333	0.184728	–0.307646
C	–3.533962	4.505422	–1.414778
C	–3.390114	3.762802	–2.697902
H	–3.815912	4.303934	–3.551114
C	–3.656529	5.997528	0.88409
C	–1.554709	–4.81268	–2.713953

C	–2.826913	4.007407	2.158962
C	–3.721675	–6.802953	–0.342196
C	–3.25798	4.657945	0.861523
C	–2.131426	–5.308981	–1.433355
C	–2.066771	–4.456079	2.141001
C	7.040778	0.514072	–1.467239
H	7.534696	0.530911	–2.434251
C	–3.961483	5.840107	–1.468736
C	–3.079913	–6.346693	–1.497068
C	7.025465	0.159813	0.883986
C	5.671369	0.812172	–1.411328
C	–4.836241	–0.077622	–2.885796
C	5.666508	0.485935	0.863483
C	–3.373158	–6.159389	0.854939
H	–3.824328	–6.414007	1.806912
C	4.955509	1.064919	–2.69307
H	5.636765	1.166148	–3.546173
C	–1.329249	–0.48719	3.10258
C	1.326878	0.472269	3.118895
N	2.292105	–2.764897	1.948366
N	3.588513	0.827775	2.071028
N	1.24339	3.361214	1.961012
N	1.437323	3.430863	–3.229523
N	2.256729	–2.946567	–3.243827
C	–2.765603	–1.006274	3.124654
H	–2.793066	–2.092158	3.214729
H	–3.316206	–0.6185	3.985025
C	–0.251122	–1.398301	3.112327
C	–1.08211	0.903411	3.116602
N	2.296181	4.303455	–0.388537
N	–3.538532	–0.611399	1.945568
N	2.583205	–4.138226	–0.408366
C	0.246129	1.381601	3.108771
C	0.514515	2.88485	3.137535
H	1.125277	3.164293	3.999365
H	–0.412181	3.451151	3.22942
C	1.076702	–0.917069	3.104897
C	0.411351	2.909403	–2.33403
H	0.540454	3.223753	–1.293021
H	–0.505585	3.375423	–2.704763
C	2.316593	–1.801495	–2.342981
H	2.524932	–2.075202	–1.30329
H	3.177953	–1.238154	–2.711497
C	2.75671	0.99521	3.262441

H	2.758362	2.047916	3.548706
H	3.278488	0.468364	4.06407
N	–4.873176	–0.165971	–0.408475
C	2.243766	–1.901721	3.129486
H	2.179589	–2.574592	3.987914
H	3.19765	–1.383352	3.224644
C	3.367688	–6.86204	–0.413052
C	2.781297	4.789672	–1.54498
C	–7.623543	0.519869	–0.410623
C	2.773073	–4.816759	0.73113
C	2.561781	–4.094794	2.045325
C	3.152184	–6.148578	–1.594921
C	2.259629	4.259624	2.064569
C	2.497914	–4.14529	–2.886072
H	2.487928	–4.876795	–3.702341
C	4.264867	6.342834	–0.382693
C	2.763244	–4.796603	–1.567286
C	2.784817	4.805416	0.753518
C	2.354316	4.237169	–2.866409
H	2.996379	4.595184	–3.67945
C	–6.90685	0.342165	0.780696
H	–7.360036	0.458685	1.758073
C	3.157646	–6.155286	0.778825
C	3.752309	5.806916	0.806469
C	–6.897734	0.354036	–1.593085
C	–5.555749	0.004568	0.731643
C	4.88784	0.432698	2.160714
C	–5.532877	0.013224	–1.566657
C	3.758991	5.801389	–1.567281
H	4.114283	6.146907	–2.531303
C	–4.824842	–0.179913	2.045114
H	–0.568842	–3.478435	1.178097
H	–3.209355	–0.872446	1.024394
H	–2.725741	2.22571	1.18921
H	0.853171	3.209665	1.039186
H	3.294879	1.241671	1.194241
H	2.356645	–2.345987	1.028927
H	–7.371396	0.490445	–2.558508
H	–3.309518	–6.774444	–2.466437
H	3.28007	–6.607933	1.755759
H	3.275125	–6.624797	–2.560853
H	7.464105	–0.102819	1.837711
H	4.079457	6.137756	1.785158
H	–4.219838	6.261903	–2.43558

H	–3.65295	6.506035	1.839225
C	–4.512544	8.082395	–0.378677
C	–3.559385	8.88152	–1.299256
H	–3.886784	9.92584	–1.363197
H	–2.535171	8.870272	–0.909966
H	–3.536595	8.477219	–2.316793
C	–5.945751	8.123768	–0.9605
H	–6.644843	7.568395	–0.325125
H	–6.295878	9.160665	–1.024371
H	–5.992085	7.694612	–1.967056
C	–4.528757	8.753427	1.007401
H	–3.53142	8.780595	1.459912
H	–4.875943	9.787825	0.909855
H	–5.204495	8.241324	1.701294
C	5.32594	7.45254	–0.342878
C	4.746606	8.67641	0.40644
H	4.456264	8.426806	1.431943
H	5.495363	9.47571	0.455847
H	3.862558	9.070964	–0.107275
C	6.57374	6.932189	0.409785
H	7.009	6.065804	–0.101105
H	7.33738	7.717331	0.457952
H	6.336603	6.63317	1.435797
C	5.75599	7.900393	–1.751993
H	6.508128	8.692651	–1.671498
H	6.20326	7.079256	–2.323813
H	4.914859	8.302464	–2.328276
C	9.257772	–0.138295	–0.380615
C	9.846168	–0.467558	1.004024
H	9.369508	–1.346314	1.452151
H	10.915262	–0.685404	0.905808
H	9.74178	0.370435	1.702091
C	9.471127	–1.358988	–1.307276
H	10.53875	–1.599375	–1.372261
H	8.947451	–2.241316	–0.922588
H	9.110121	–1.170671	–2.323896
C	10.012619	1.083791	–0.956103
H	9.882809	1.963849	–0.316166
H	11.085276	0.866767	–1.020789
H	9.664941	1.344445	–1.961445
C	3.7998	–8.335454	–0.378502
C	2.724792	–9.16017	0.368961
H	1.75769	–9.103096	–0.143398
H	3.024024	–10.213949	0.413588

H	2.58209	–8.809514	1.39598
C	3.975821	–8.926114	–1.789694
H	4.287128	–9.973504	–1.712649
H	3.042188	–8.902016	–2.363326
H	4.74497	–8.395433	–2.362482
C	5.148126	–8.447204	0.372681
H	5.466909	–9.495128	0.418468
H	5.932069	–7.876028	–0.137502
H	5.074998	–8.075072	1.39964
C	–4.763892	–7.931595	–0.343291
C	–6.106086	–7.380877	0.195075
H	–6.482655	–6.571066	–0.440065
H	–6.859128	–8.177493	0.211933
H	–6.008495	–6.991753	1.21362
C	–5.001079	–8.506377	–1.751964
H	–5.744887	–9.308855	–1.700329
H	–5.383422	–7.748101	–2.444903
H	–4.086081	–8.932647	–2.179078
C	–4.270965	–9.074233	0.576536
H	–4.119188	–8.733164	1.605561
H	–5.009484	–9.884158	0.598015
H	–3.322698	–9.489189	0.216264
C	–9.115164	0.883979	–0.375614
C	–9.291161	2.224353	0.3774
H	–8.75671	3.034909	–0.130971
H	– 10.353136	2.493131	0.422011
H	–8.917309	2.1678	1.404648
C	–9.8879	–0.23009	0.370367
H	– 10.954641	0.018714	0.416159
H	–9.78573	–1.19268	–0.143513
H	–9.530002	–0.357235	1.397118
C	–9.713114	1.033459	–1.786796
H	– 10.775563	1.288557	–1.709965
H	–9.22367	1.831717	–2.356472
H	–9.638657	0.104305	–2.363313
O	–2.737607	4.664829	3.196061
O	2.753307	4.611851	3.135759
O	5.412284	0.021543	3.195857
O	2.619405	–4.703567	3.113665
O	–2.684466	–4.708408	3.175746
O	–5.380374	0.070214	3.114708
H	–5.464645	0.300351	–3.700472
H	–1.808246	–5.449966	–3.569241

Supplementary References

1. Thierer LM, *et al.* Pyridyldiimine macrocyclic ligands: Influences of template ion, linker length and imine substitution on ligand synthesis, structure and redox properties. *Polyhedron* **198**, 115044 (2021).
2. Zhang L, Tang Y, Han Z, Ding K. Lutidine-Based Chiral Pincer Manganese Catalysts for Enantioselective Hydrogenation of Ketones. *Angew. Chem. Int. Ed.* **58**, 4973-4977 (2019).
3. Liu C, *et al.* Transformation of Porous Organic Cages and Covalent Organic Frameworks with Efficient Iodine Vapor Capture Performance. *J. Am. Chem. Soc.* **144**, 12390-12399 (2022).
4. Li X, *et al.* Moderate and Universal Synthesis of Undoped Covalent Organic Framework Aerogels for Enhanced Iodine Uptake. *Chem. Mater.* **34**, 11062-11071 (2022).
5. Xie L, *et al.* Calix[4]pyrrole-based Crosslinked Polymer Networks for Highly Effective Iodine Adsorption from Water. *Angew. Chem. Int. Ed.* **61**, e202113724 (2022).
6. Yazdankish E, Foroughi M, Azqhandi MHA. Capture of I(131) from medical-based wastewater using the highly effective and recyclable adsorbent of g-C(3)N(4) assembled with Mg-Co-Al-layered double hydroxide. *J. Hazard. Mater.* **389**, 122151 (2020).
7. Harijan DKL, Chandra V, Yoon T, Kim KS. Radioactive iodine capture and storage from water using magnetite nanoparticles encapsulated in polypyrrole. *J. Hazard. Mater.* **344**, 576-584 (2018).
8. Yin Y, *et al.* Ultrafast solid-phase synthesis of 2D pyrene-alkadiyne frameworks towards efficient capture of radioactive iodine. *Chem. Eng. J.* **441**, 135996 (2022).
9. Avais M, Chattopadhyay S. Porous polyaminoamides via an exotemplate synthesis approach for ultrahigh multimedia iodine adsorption. *J. Mater. Chem. A* **10**, 20090-20100 (2022).
10. Jiang X, *et al.* Topochemical Synthesis of Single-Crystalline Hydrogen-Bonded Cross-Linked Organic Frameworks and Their Guest-Induced Elastic Expansion. *J. Am. Chem. Soc.* **141**, 10915-10923 (2019).
11. Yu CX, *et al.* Fabrication of Protonated Two-Dimensional Metal-Organic Framework Nanosheets for Highly Efficient Iodine Capture from Water. *Inorg. Chem.* **61**, 13883-13892 (2022).
12. Chen D, Ma T, Zhao X, Jing X, Zhao R, Zhu G. Multi-Functionalization Integration into the Electrospun Nanofibers Exhibiting Effective Iodine Capture from Water. *ACS Appl. Mater. Interfaces* **14**, 47126-47135 (2022).
13. Sen A, *et al.* Functionalized Ionic Porous Organic Polymers Exhibiting High Iodine Uptake from Both the Vapor and Aqueous Medium. *ACS Appl. Mater. Interfaces* **13**, 34188-34196 (2021).
14. Xu XH, Li YX, Zhou L, Liu N, Wu ZQ. Precise fabrication of porous polymer frameworks using rigid polyisocyanides as building blocks: from structural regulation to efficient iodine capture. *Chem. Sci.* **13**, 1111-1118 (2022).
15. An D, Li L, Zhang Z, Asiri AM, Alamry KA, Zhang X. Amino-bridged covalent organic Polycalix[4]arenes for ultra efficient adsorption of iodine in water. *Mater. Chem. Phys.* **239**, 122328 (2020).
16. Samanta P, Dutta S, Let S, Sen A, Shirolkar MM, Ghosh SK. Hydroxy-Functionalized Hypercrosslinked Polymers (HCPs) as Dual Phase Radioactive Iodine Scavengers: Synergy of Porosity and Functionality. *Chempluschem* **87**, e202200212 (2022).
17. Zhang Z, Li L, An D, Li H, Zhang X. Triazine-based covalent organic polycalix[4]arenes for highly efficient and reversible iodine capture in water. *J. Mater. Sci.* **55**, 1854-1864 (2019).
18. Das M, Sarkar SK, Patra YS, Manna A, Mukherjee S, Das S. Soft Self-Templating Approach-Derived Covalent Triazine Framework with Bimodal Nanoporosity for Efficient Radioactive Iodine Capture for Safe Nuclear Energy. *ACS Appl. Nano Mater.* **5**, 8783-8793 (2022).
19. Lin Y, *et al.* An Elastic Hydrogen-Bonded Cross-Linked Organic Framework for Effective Iodine Capture

in Water. *J. Am. Chem. Soc.* **139**, 7172-7175 (2017).
20. Au-Duong A-N, Lee C-K. Flexible Metal–Organic Framework-Bacterial Cellulose Nanocomposite for Iodine Capture. *Cryst. Growth Des.* **18**, 356-363 (2017).
21. Ma Z, Han Y, Qi J, Qu Z, Wang X. High iodine adsorption by lignin-based hierarchically porous flower-like carbon nanosheets. *Ind. Crop. Prod.* **169**, 113649 (2021).
22. Bestani B, Benderdouche N, Benstaali B, Belhakem M, Addou A. Methylene blue and iodine adsorption onto an activated desert plant. *Bioresour. Technol.* **99**, 8441-8444 (2008).
23. Gogia A, Das P, Mandal SK. Tunable Strategies Involving Flexibility and Angularity of Dual Linkers for a 3D Metal-Organic Framework Capable of Multimedia Iodine Capture. *ACS Appl. Mater. Interfaces* **12**, 46107-46118 (2020).
24. Li A, Liu Y, Zhou W, Jiang Y, He Q. Superphanes: Facile and efficient preparation, functionalization and unique properties. *Tetrahedron Chem* **1**, 100006 (2022).
25. Dolomanov OV, Bourhis LJ, Gildea RJ, Howard JAK, Puschmann H. OLEX2: a complete structure solution, refinement and analysis program. *J. Appl. Crystallogr.* **42**, 339-341 (2009).
26. Sheldrick GM. SHELXT - Integrated space-group and crystal-structure determination. *Acta Crystallogr. A* **71**, 3-8 (2015).
27. Sheldrick GM. Crystal structure refinement with SHELXL. *Acta Crystallogr. C* **71**, 3-8 (2015).
